# Predicting potential adverse events using safety data from marketed drugs

**DOI:** 10.1186/s12859-020-3509-7

**Published:** 2020-04-29

**Authors:** Chathuri Daluwatte, Peter Schotland, David G. Strauss, Keith K. Burkhart, Rebecca Racz

**Affiliations:** 1 Division of Applied Regulatory Science, Food and Drug Administration, 10903 New Hampshire Ave, Silver Spring, MD 20993 USA; 2 Office of New Drugs, Food and Drug Administration, Silver Spring, MD USA

**Keywords:** Adverse reaction, Pharmacovigilance, Classifier, Computational biology

## Abstract

**Background:**

While clinical trials are considered the gold standard for detecting adverse events, often these trials are not sufficiently powered to detect difficult to observe adverse events. We developed a preliminary approach to predict 135 adverse events using post-market safety data from marketed drugs. Adverse event information available from FDA product labels and scientific literature for drugs that have the same activity at one or more of the same targets, structural and target similarities, and the duration of post market experience were used as features for a classifier algorithm. The proposed method was studied using 54 drugs and a probabilistic approach of performance evaluation using bootstrapping with 10,000 iterations.

**Results:**

Out of 135 adverse events, 53 had high probability of having high positive predictive value. Cross validation showed that 32% of the model-predicted safety label changes occurred within four to nine years of approval (median: six years).

**Conclusions:**

This approach predicts 53 serious adverse events with high positive predictive values where well-characterized target-event relationships exist. Adverse events with well-defined target-event associations were better predicted compared to adverse events that may be idiosyncratic or related to secondary target effects that were poorly captured. Further enhancement of this model with additional features, such as target prediction and drug binding data, may increase accuracy.

## Background

The Food and Drug Administration’s (FDA) proposed process modernization to support new drug development involves establishing a unified post-market safety surveillance framework to monitor the benefits and risks of drugs across their lifecycles [1]. While clinical trials are considered the gold standard for detecting and labeling adverse events, these trials are not sufficiently powered to detect less common adverse events. Additionally, some adverse events emerge when a drug is used in clinical practice outside of the specified inclusion/exclusion criteria. Some adverse events may have high prevalence in specific subpopulations who were not enrolled in the clinical trials or subgroups who cannot be identified based on information collected from patients in the trials. For example, a substantially increased risk of Stevens-Johnson syndrome in patients positive for the HLA-B*1502 allele taking carbamazepine was not identified until decades after approval [2]. In addition, concomitant medications (drug-drug interactions) and comorbidities may also contribute to adverse events, and these interactions are not always adequately present or captured in clinical trials. Therefore, post-market safety surveillance is crucial.

FDA uses the FDA Adverse Event Reporting System (FAERS) [3] and the Sentinel Initiative [4] to obtain information about adverse events occurring after drug approval. In 2017, over 1.8 million adverse event cases were reported to the FDA, including nearly 907,000 serious reports and over 164,000 fatal cases [5]. While traditional pharmacovigilance relies on data mining systems, these methods have reporting biases and require manual review of cases to determine reporting accuracy. Recently, there has been a strong interest in developing prediction algorithms to assist in post-market surveillance to overcome such weaknesses and make post-market pharmacovigilance more efficient.

Adverse event information from a variety of sources such as FAERS, literature, genomic data, and social media has been used to both evaluate adverse events and make predictions. For example, FAERS and similar post-market databases have demonstrated utility in adverse event prediction; Xu and Wang showed FAERS, combined with literature, had great utility in detecting safety signals [6]. Others have used chemical structure as the basis for adverse event predictions. Vilar and colleagues used molecular fingerprint similarity to drugs with a known association with rhabdomyolysis to further support and prioritize rhabdomyolysis signals found in FAERS [7]. Another unique option has been to use social media reports to identify new adverse events for drugs before they are reported to regulatory agencies or in peer-reviewed literature; Yang and colleagues used a partially supervised classification method to identify reports of adverse events on the discussion forum for Medhelp [3]. Other sources of information for adverse event prediction and detection include electronic health records, drug labels and even bioassay data [8–10]. Additionally, a wide variety of algorithms have been used to make adverse event predictions, including logistic regression models, support vector machine, and ensemble methods [8, 11, 12]. Many of these models have experienced varying degrees of success but overall demonstrate the great potential of developing an adverse event prediction model using a classifier.

However, many of these methodologies have focused on predicting a specific adverse event (e.g. cardiovascular events) or drug class (e.g. oncology drugs) [12–14]. Algorithms that can predict a wide variety of adverse events for multiple drug classes are important to enhance post-market safety surveillance. We have previously developed a genetic algorithm to predict approximately 900 adverse events using FDA product labels and FAERS data [15]. In this study, we build on this algorithm to predict 135 adverse events of high priority to regulatory review using safety data from marketed drugs with one or more shared molecular targets. We hypothesize that drugs that have similar modes of action at the same targets will have a similar adverse event profile because of shared structural features and likely target binding characteristics. We additionally expect adverse events that are more closely associated with drug targets (such as serotonin syndrome) to be well-predicted via this methodology. Some idiosyncratic reactions may also be captured well because the shared structural features likely play a role in these reactions where the targets and actions have not yet been fully characterized.

## Results

**Fig. 1:**
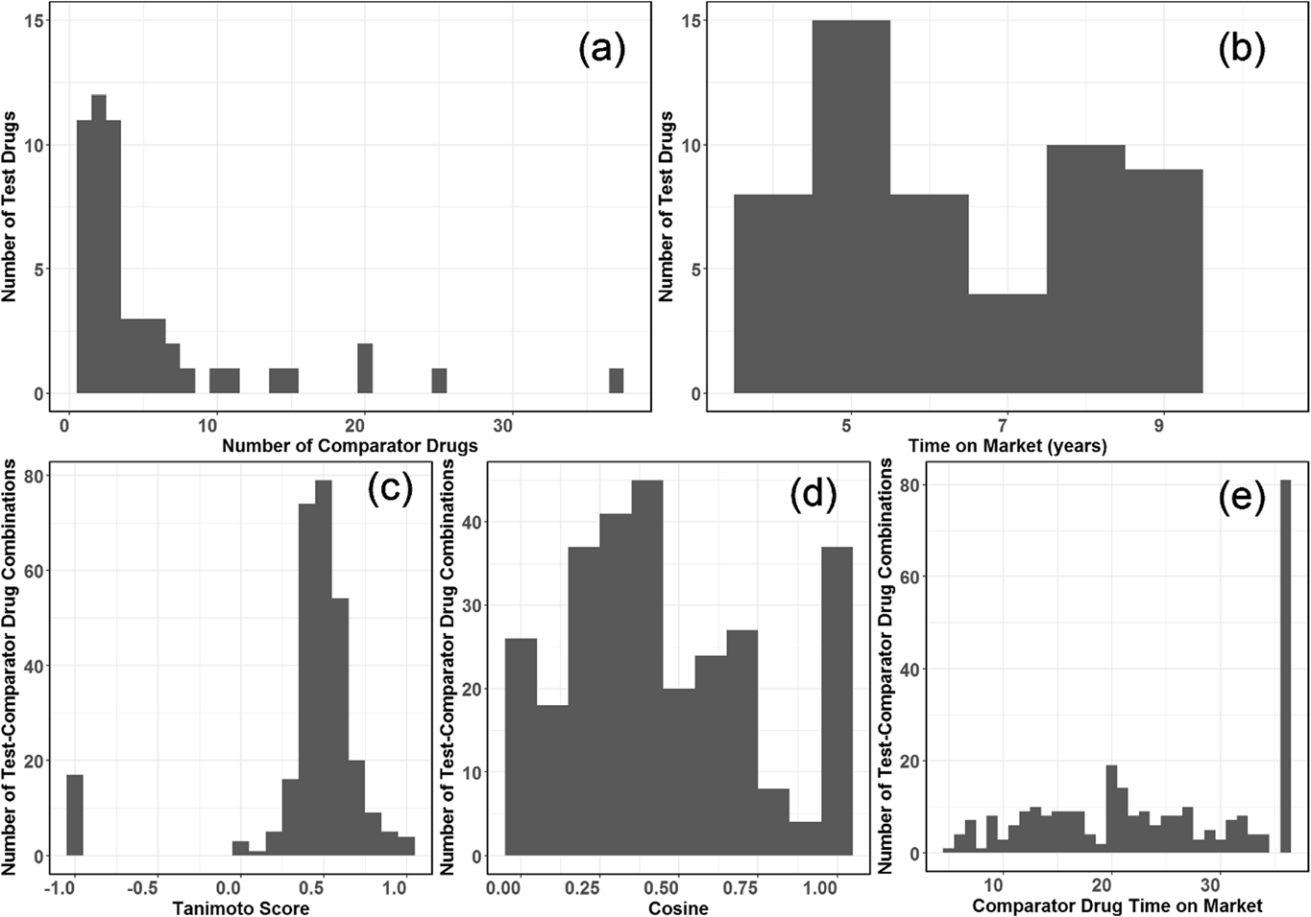
Characteristics of test drugs, comparator drugs and test-comparator drug combinations. **a**) Distribution of number of comparator drugs for test drug. **b**) Distribution of time on market for test drugs. **c**) Tanimoto score distribution for test-comparator drug combinations. **d**) Target similarity score distribution for test-comparator drug combinations. **e**) Distribution of time on market for comparator drugs

Inclusion and exclusion criteria resulted in 54 test drugs and 213 unique comparator drugs, leading to 287 test-comparator drug combinations. The 54 test drugs used in this study had one to 37 comparator drugs, with one and two comparators being most frequent, as identified by DrugBank (Fig. 1a), and were on the market four to nine years (Fig. 1b). Tanimoto similarity scores between test drugs and comparator drugs ranged between 0.02 and 1, with 0.51 being the mean and 0.5 being the mode. Eighteen test drug-comparator associations included a biologic, as defined by a − 1 Tanimoto score (Fig. 1c). Target cosine similarity scores between test drugs and comparator drugs ranged between 0 and 1, with 0.45 being the mean and 1 being the mode (Fig. 1d). Seventy-nine comparator drugs were approved before 1982, while the most recently approved comparator drug had five years of time in market (Fig. 1e). The 54 test drugs are known to bind to 126 targets based on DrugBank data (summarized in Supplemental Table 1).

**Fig. 2:**
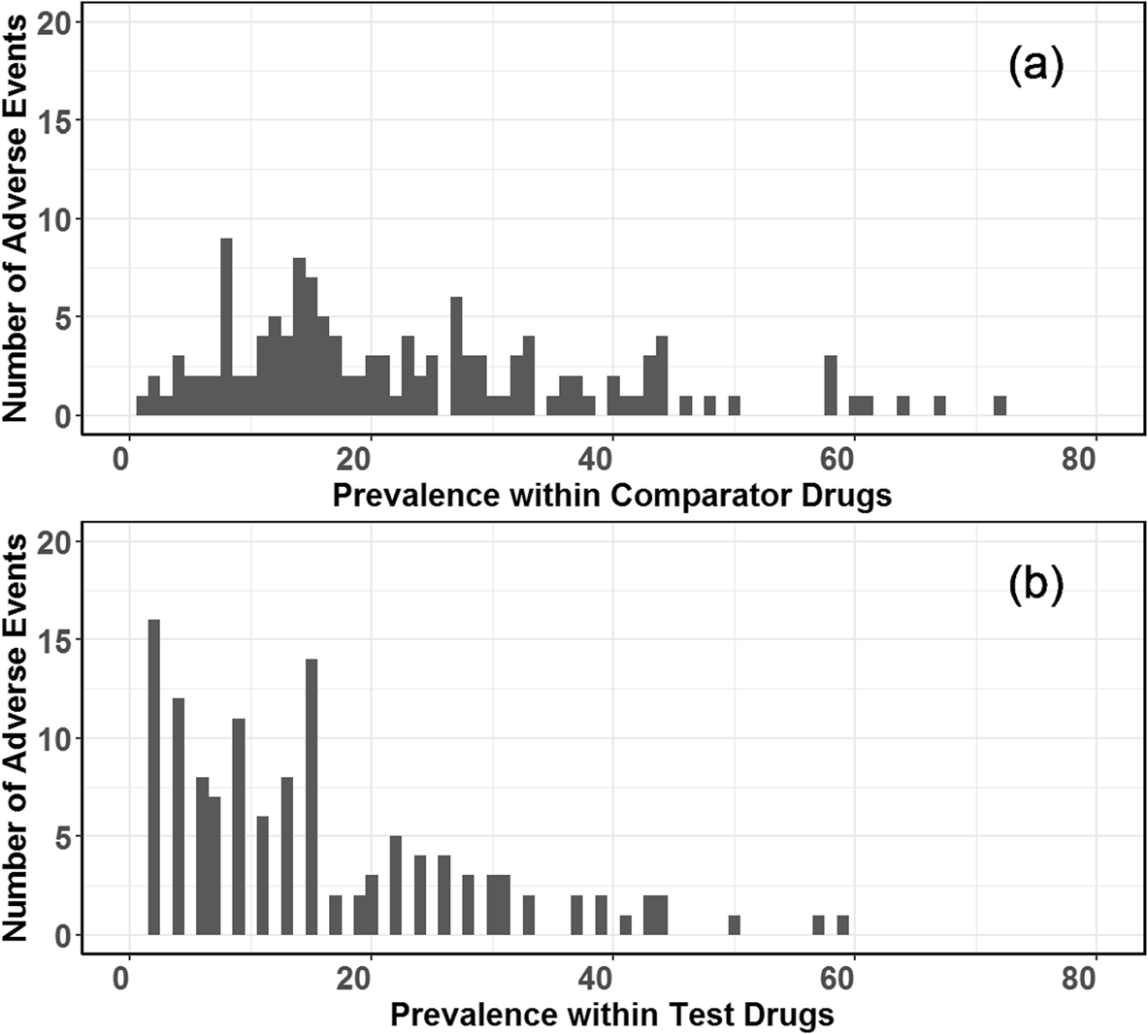
Prevalence of adverse events within comparator drugs and test drugs

The prevalence of the 135 adverse events considered in this study is summarized in Fig. 2. The overall prevalence of adverse events was higher in the comparator drugs.

Prediction models were not made for 26 adverse events that were not observed or observed only in one test drug label (accident, anaphylactoid reaction, aplastic anaemia, apnoea, atrioventricular block, azotaemia, cardiomyopathy, cerebral infarction, coagulopathy, colitis, colitis ulcerative, Crohn’s disease, dermatitis bullous, dermatitis exfoliative, gastric ulcer, granulocytopenia, hepatic necrosis, hypokinesia, injury, myopathy, oliguria, respiratory depression, road traffic accident, skin ulcer, thrombosis, and ulcer).

**Fig. 3:**
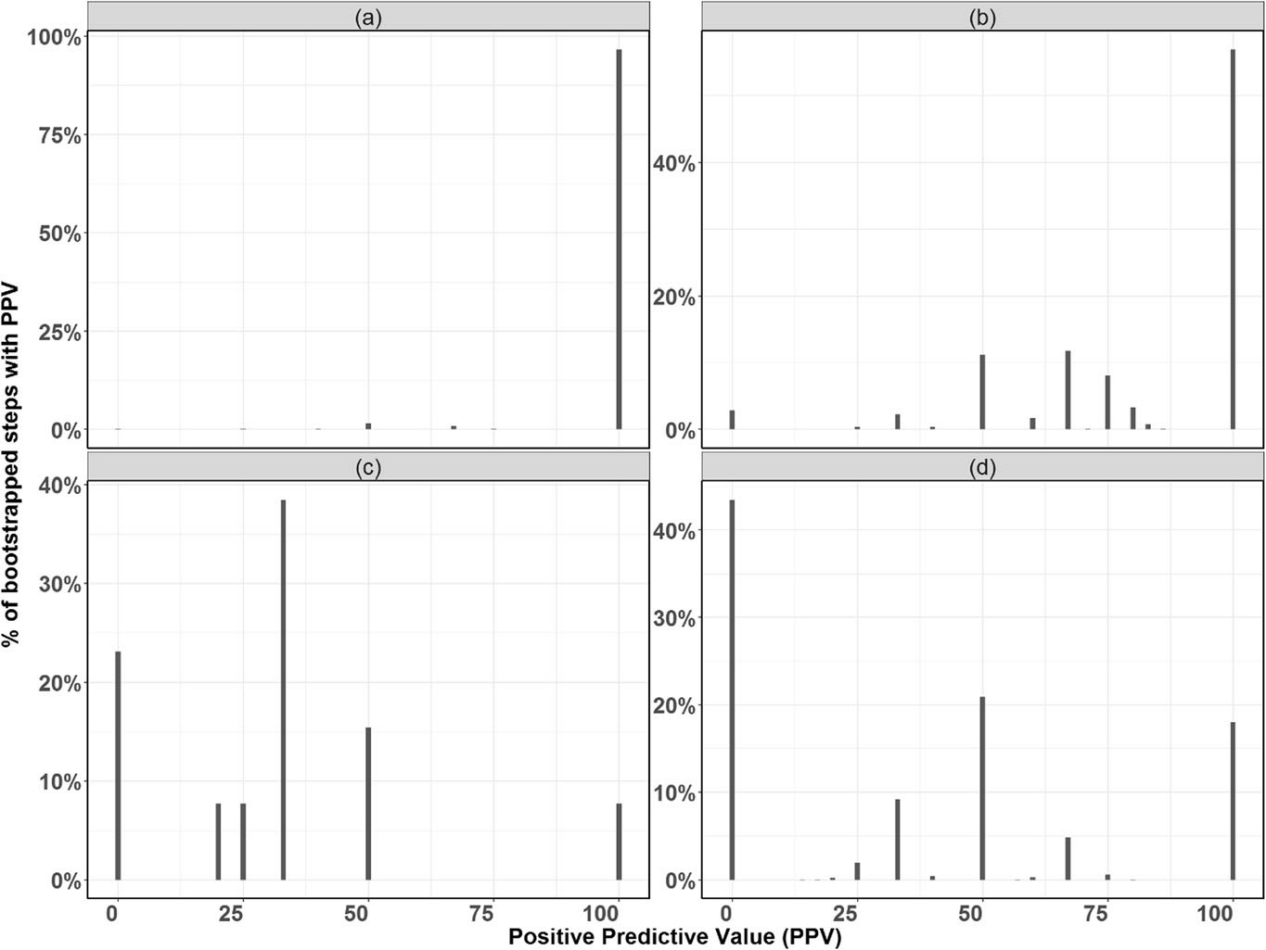
Left-skewed positive predictive value histograms demonstrated well-predicted adverse events, as shown in **a**) Febrile Neutropenia and **b**) Hypertension. Right-skewed positive predictive value histograms demonstrated poorly-predicted adverse events, as shown in **c**) Bacterial Infection and **d**) Haemorrhage

Results at varying thresholds (the minimum percentage of comparator drugs which are predicted positive for an adverse event to result in a positive prediction) for the safety label change evaluation and the number of adverse events with left-skewed positive predictive value, which demonstrated a high probability for high positive predictive value, are summarized in Table 1. Based on these results, we selected 70% as the optimum threshold. This resulted in the highest number of adverse events with high positive predictive values along with a high percentage of predicted safety label changes that were also issued by FDA (32%). All performance histograms at 70% threshold for each adverse event are provided in supplementary materials. Positive predictive value histograms of two well-predicted (i.e. left-skewed histograms) adverse events (febrile neutropenia and hypertension) and two poorly-predicted (i.e. right-skewed histograms) adverse events (bacterial infection and haemorrhage) are shown in Fig. 3.

**Table 1:** Performance of the algorithm when the threshold to make a positive prediction was varied

Threshold	FDA-issued safety label changes that were correctly predicted (%)	Predicted safety label changes that were also FDA-issued (%)	Number of adverse events with a high positive predictive value
0	43	13	11
10	39	14	19
30	32	18	28
50	18	28	42
60	17	29	49
70	13	32	53
90	11	34	48

Fifty-three adverse events showed 100% as the positive predictive value mode, with the median between 50 and 100, 25% quantile between 0 and 100, and 75% quantile at 100%, which suggests left-skewed distributions. By having a left-skewed distribution for positive predictive value, these adverse events were considered well-predicted, which suggests high probability of having high positive predictive value (Table 2). Additionally, these adverse events had a sensitivity mode between 0 and 100%, specificity mode of 100%, and negative predictive value mode of 50–100%.

**Table 2:** Performance and prevalence of adverse events that were well-predicted by the algorithm

Adverse Event	Median (25th – 75th quantile) Mode				Prevalence (%)	
	Sensitivity	Specificity	Positive Predictive Value	Negative Predictive Value	Comparator Drugs	Test Drugs
**AGRANULOCYTOSIS**	50 (50–100) 100	100 (100–100) 100	100 (100–100) 100	91 (82–91) 91	23	11
**ANAEMIA**	25 (17–33) 20	100 (100–100) 100	100 (100–100) 100	55 (45–64) 55	45	42
**ANAPHYLACTIC REACTION**	25 (20–33) 25	100 (100–100) 100	100 (100–100) 100	64 (56–73) 73	37	35
**BONE MARROW FAILURE**	100 (0–100) 100	100 (90–100) 100	100 (0–100) 100	91 (91–91) 91	14	5
**BRONCHITIS**	33 (25–50) 50	100 (100–100) 100	100 (100–100) 100	82 (73–90) 82	27	20
**CEREBRAL HAEMORRHAGE**	33 (0–50) 0	100 (90–100) 100	100 (0–100) 100	82 (82–91) 91	14	15
**CHOLESTASIS**	50 (50–100) 100	100 (100–100) 100	100 (100–100) 100	91 (82–91) 91	8	9
**CONFUSIONAL STATE**	25 (20–33) 25	100 (100–100) 100	100 (100–100) 100	70 (60–80) 73	50	33
**DEEP VEIN THROMBOSIS**	50 (33–100) 50	100 (100–100) 100	100 (100–100) 100	90 (82–91) 91	11	13
**DIABETES MELLITUS**	50 (0–100) 0	90 (90–100) 100	50 (0–100) 100	91 (82–91) 91	27	9
**DIPLOPIA**	100 (100–100) 100	100 (100–100) 100	100 (100–100) 100	91 (91–100) 91	17	4
**EXTRAPYRAMIDAL DISORDER**	100 (100–100) 100	100 (100–100) 100	100 (100–100) 100	91 (91–91) 91	21	4
**FEBRILE NEUTROPENIA**	50 (33–100) 50	100 (100–100) 100	100 (100–100) 100	90 (82–91) 91	7	15
**GASTROINTESTINAL HAEMORRHAGE**	33 (25–50) 25	100 (100–100) 100	100 (100–100) 100	73 (64–82) 73	27	31
**HEPATIC FAILURE**	33 (25–50) 33	100 (100–100) 100	100 (100–100) 100	73 (64–82) 73	29	25
**HEPATOTOXICITY**	25 (0–33) 0	90 (88–100) 100	50 (0–100) 100	73 (67–82) 73	25	27
**HYPERCHOLESTEROLAEMIA**	33 (0–50) 0	90 (89–100) 100	50 (0–100) 100	82 (80–91) 91	22	15
**HYPERGLYCAEMIA**	25 (17–40) 0	100 (89–100) 100	100 (50–100) 100	78 (67–82) 80	36	27
**HYPERKINESIA**	50 (50–100) 100	100 (100–100) 100	100 (100–100) 100	90 (82–91) 91	36	13
**HYPERSENSITIVITY**	20 (14–29) 17	100 (80–100) 100	100 (50–100) 100	44 (33–56) 50	67	58
**HYPERTENSION**	50 (33–67) 50	100 (86–100) 100	100 (67–100) 100	75 (62–83) 67	57	40
**HYPOGLYCAEMIA**	50 (25–100) 50	100 (90–100) 100	100 (50–100) 100	90 (82–91) 91	27	15
**IMPAIRED HEALING**	100 (50–100) 100	100 (100–100) 100	100 (100–100) 100	91 (90–91) 91	2	7
**INFECTION**	50 (33–67) 50	89 (86–100) 100	67 (50–100) 100	80 (73–89) 100	43	27
**INSOMNIA**	20 (14–25) 17	100 (100–100) 100	100 (100–100) 100	55 (45–64) 55	58	49
**INTERSTITIAL LUNG DISEASE**	50 (33–67) 50	100 (89–100) 100	100 (50–100) 100	89 (80–91) 90	12	18
**JAUNDICE**	33 (0–50) 0	90 (89–100) 100	50 (0–100) 100	82 (80–91) 91	40	15
**LARYNGEAL OEDEMA**	100 (67–100) 100	88 (79–100) 100	50 (33–100) 100	91 (82–91) 91	8	7
**LEUKOPENIA**	25 (25–33) 25	100 (100–100) 100	100 (100–100) 100	73 (64–82) 73	44	29
**MYOCARDIAL INFARCTION**	25 (0–40) 0	100 (88–100) 100	100 (0–100) 100	73 (67–82) 80	44	29
**NEUROLEPTIC MALIGNANT SYNDROME**	100 (100–100) 100	100 (100–100) 100	100 (100–100) 100	91 (91–100) 91	16	4
**NEUROPATHY PERIPHERAL**	40 (25–50) 50	86 (80–100) 100	67 (50–100) 100	67 (56–78) 67	60	42
**NEUTROPENIA**	50 (33–60) 50	100 (100–100) 100	100 (100–100) 100	75 (67–82) 78	23	38
**OEDEMA**	20 (14–33) 0	83 (75–100) 100	67 (50–100) 100	44 (33–55) 50	71	58
**PANCREATITIS**	50 (25–60) 50	90 (86–100) 100	75 (50–100) 100	80 (70–89) 78	33	29
**PANCYTOPENIA**	50 (25–67) 50	100 (90–100) 100	100 (50–100) 100	90 (82–91) 91	21	15
**PNEUMONIA**	50 (25–67) 50	89 (86–100) 100	50 (33–100) 100	86 (78–90) 100	37	22
**PROTEINURIA**	50 (33–50) 50	100 (100–100) 100	100 (100–100) 100	89 (82–91) 91	12	15
**PULMONARY EMBOLISM**	50 (25–100) 50	100 (90–100) 100	100 (50–100) 100	90 (82–91) 91	18	13
**PULMONARY OEDEMA**	50 (0–100) 0	90 (90–100) 100	50 (0–100) 100	90 (82–91) 91	17	9
**RENAL FAILURE**	50 (33–67) 50	100 (100–100) 100	100 (100–100) 100	89 (80–91) 91	28	18
**RENAL IMPAIRMENT**	50 (33–100) 50	100 (100–100) 100	100 (100–100) 100	91 (82–91) 91	27	11
**SEIZURE**	33 (0–50) 0	89 (86–100) 100	50 (0–100) 100	78 (70–88) 80	49	27
**SEPSIS**	50 (33–67) 50	100 (100–100) 100	100 (100–100) 100	88 (78–90) 100	20	24
**SEROTONIN SYNDROME**	100 (50–100) 100	100 (100–100) 100	100 (100–100) 100	91 (91–100) 100	13	7
**STEVENS-JOHNSON SYNDROME**	33 (20–50) 33	100 (90–100) 100	100 (50–100) 100	80 (73–89) 82	28	24
**STOMATITIS**	33 (0–50) 0	89 (86–100) 100	50 (0–100) 100	82 (75–90) 80	28	22
**SUICIDAL BEHAVIOUR**	25 (20–33) 33	100 (100–100) 100	100 (100–100) 100	82 (73–82) 82	24	22
**SUPRAVENTRICULAR TACHYCARDIA**	33 (25–50) 33	100 (100–100) 100	100 (100–100) 100	80 (73–90) 82	27	24
**TACHYCARDIA**	40 (25–50) 50	90 (86–100) 100	75 (50–100) 100	78 (67–88) 78	61	31
**THROMBOCYTOPENIA**	40 (25–50) 50	88 (83–100) 100	67 (50–100) 100	73 (62–80) 67	58	36
**UPPER RESPIRATORY TRACT INFECTION**	20 (17–33) 20	100 (86–100) 100	100 (50–100) 100	60 (50–70) 60	28	44
**URINARY TRACT INFECTION**	25 (20–40) 25	100 (88–100) 100	100 (67–100) 100	67 (56–75) 70	32	40

Fifty-six adverse events had positive predictive values mode between 0 and 33%, which suggested right-skewed distribution and thus were considered poorly-predicted (Table 3). While the positive predictive value was low, all these adverse events did have high specificity (mode: 76–100%) and negative predictive value (mode: 55–91%). Two adverse events, bacterial infection and fungal infection, additionally had high sensitivity (mode: 100%) (Table 3).

**Table 3:** Performance and prevalence of adverse events that were poorly-predicted by the algorithm

Adverse Event	Median (25th – 75th quantile) Mode				Prevalence (%)	
	Sensitivity	Specificity	Positive Predictive Value	Negative Predictive Value	Comparator Drugs	Test Drugs
**ACUTE KIDNEY INJURY**	0 (0–0) 0	88 (86–89) 88	0 (0–0) 0	73 (64–82) 73	23	27
**AGGRESSION**	0 (0–0) 0	78 (67–89) 89	0 (0–0) 0	82 (82–91) 91	22	15
**AMNESIA**	0 (0–0) 0	80 (79–89) 80	0 (0–0) 0	91 (82–91) 91	20	7
**ANGINA PECTORIS**	0 (0–50) 0	90 (88–100) 100	0 (0–100) 0	82 (73–91) 82	33	16
**ANGIOEDEMA**	0 (0–0) 0	86 (83–88) 83	0 (0–0) 0	55 (45–64) 55	45	45
**ARRHYTHMIA**	0 (0–0) 0	90 (89–90) 90	0 (0–0) 0	82 (82–91) 91	41	13
**BACTERIAL INFECTION**	75 (31–100) 100	80 (78–90) 80	33 (20–33) 33	91 (82–91) 91	3	11
**BLINDNESS**	0 (0–0) 0	80 (75–80) 80	0 (0–0) 0	91 (82–91) 91	8	11
**BRADYCARDIA**	0 (0–0) 0	90 (89–90) 90	0 (0–0) 0	91 (82–91) 91	32	11
**CANDIDA INFECTION**	0 (0–0) 0	90 (82–90) 90	0 (0–0) 0	91 (91–91) 91	8	5
**CARDIAC ARREST**	0 (0–0) 0	90 (78–90) 90	0 (0–0) 0	91 (82–91) 91	21	9
**CARDIAC FAILURE**	0 (0–33) 0	89 (88–90) 100	0 (0–67) 0	73 (64–82) 73	40	25
**CATARACT**	0 (0–0) 0	78 (70–80) 78	0 (0–0) 0	91 (82–91) 91	16	11
**CELLULITIS**	0 (0–0) 0	90 (89–90) 90	0 (0–0) 0	91 (82–91) 91	12	9
**CEREBROVASCULAR ACCIDENT**	0 (0–33) 0	89 (88–100) 89	0 (0–100) 0	80 (73–82) 82	35	22
**CONJUNCTIVITIS**	0 (0–0) 0	80 (70–80) 80	0 (0–0) 0	82 (82–91) 91	29	13
**DEAFNESS**	0 (0–0) 0	80 (70–80) 80	0 (0–0) 0	91 (82–91) 91	15	7
**DELIRIUM**	0 (0–0) 0	75 (70–80) 76	0 (0–0) 0	82 (82–91) 91	14	13
**DELUSION**	0 (0–0) 0	80 (72–80) 80	0 (0–0) 0	91 (91–91) 91	13	4
**DISORIENTATION**	0 (0–0) 0	80 (80–85) 80	0 (0–0) 0	91 (91–91) 91	14	5
**DRUG REACTION WITH EOSINOPHILIA AND SYSTEMIC SYMPTOMS**	0 (0–0) 0	80 (78–89) 78	0 (0–0) 0	91 (82–91) 91	4	11
**DYSGEUSIA**	0 (0–0) 0	89 (86–90) 89	0 (0–0) 0	80 (73–89) 82	31	20
**ELECTROCARDIOGRAM QT PROLONGED**	0 (0–0) 0	89 (89–90) 90	0 (0–0) 0	82 (80–91) 82	20	15
**EMBOLISM**	0 (0–0) 0	90 (90–90) 90	0 (0–0) 0	91 (90–91) 91	6	5
**EOSINOPHILIA**	0 (0–0) 0	90 (89–90) 90	0 (0–0) 0	91 (82–91) 91	24	9
**ERYTHEMA MULTIFORME**	0 (0–0) 0	80 (78–80) 80	0 (0–0) 0	91 (82–91) 91	25	9
**FALL**	0 (0–0) 0	78 (67–89) 90	0 (0–0) 0	82 (82–91) 91	0	13
**FRACTURE**	0 (0–0) 0	90 (90–90) 90	0 (0–0) 0	91 (90–91) 91	7	5
**FUNGAL INFECTION**	100 (50–100) 100	80 (78–89) 80	33 (22–50) 33	91 (82–91) 91	6	9
**GLAUCOMA**	0 (0–0) 0	80 (80–90) 80	0 (0–0) 0	91 (91–91) 91	14	5
**HAEMATOMA**	0 (0–0) 0	89 (88–89) 89	0 (0–0) 0	82 (73–82) 82	16	20
**HAEMOLYTIC ANAEMIA**	0 (0–0) 0	90 (90–90) 90	0 (0–0) 0	91 (91–91) 91	15	4
**HAEMORRHAGE**	17 (0–33) 0	86 (75–88) 100	33 (0–50) 0	64 (56–73) 60	44	36
**HALLUCINATION**	0 (0–0) 0	89 (89–90) 90	0 (0–0) 0	82 (80–91) 91	33	15
**HEPATITIS**	0 (0–25) 0	88 (83–90) 89	0 (0–50) 0	78 (70–82) 80	43	24
**HOSTILITY**	0 (0–0) 0	80 (78–88) 80	0 (0–0) 0	91 (82–91) 91	16	9
**MEMORY IMPAIRMENT**	0 (0–0) 0	79 (70–80) 80	0 (0–0) 0	82 (82–91) 91	11	15
**MYOSITIS**	0 (0–0) 0	80 (80–90) 80	0 (0–0) 0	91 (91–91) 91	5	4
**PARALYSIS**	0 (0–0) 0	80 (78–90) 80	0 (0–0) 0	91 (82–91) 91	10	7
**PARANOIA**	0 (0–0) 0	80 (80–90) 85	0 (0–0) 0	91 (91–91) 91	11	4
**PHOTOSENSITIVITY REACTION**	0 (0–0) 0	90 (90–90) 90	0 (0–0) 0	91 (90–91) 91	34	5
**RECTAL HAEMORRHAGE**	0 (0–0) 0	80 (80–90) 90	0 (0–0) 0	91 (91–91) 91	15	5
**RESPIRATORY FAILURE**	0 (0–0) 0	89 (89–90) 90	0 (0–0) 0	82 (80–91) 91	16	15
**RHABDOMYOLYSIS**	0 (0–0) 0	89 (80–90) 90	0 (0–0) 0	91 (82–91) 91	19	11
**SLEEP DISORDER**	0 (0–0) 0	80 (80–90) 80	0 (0–0) 0	91 (91–91) 91	13	4
**SUDDEN DEATH**	0 (0–0) 0	80 (70–80) 80	0 (0–0) 0	91 (91–91) 91	14	4
**THROMBOPHLEBITIS**	0 (0–0) 0	90 (90–90) 90	0 (0–0) 0	91 (91–91) 91	12	4
**TINNITUS**	0 (0–0) 0	89 (89–90) 90	0 (0–0) 0	82 (80–91) 91	41	15
**TOXIC EPIDERMAL NECROLYSIS**	0 (0–50) 0	90 (89–100) 100	0 (0–100) 0	84 (80–91) 91	25	15
**URTICARIA**	25 (0–40) 0	88 (80–90) 100	50 (0–75) 0	70 (60–80) 70	64	33
**VAGINAL HAEMORRHAGE**	0 (0–0) 0	90 (90–90) 90	0 (0–0) 0	91 (91–91) 91	9	4
**VASCULITIS**	0 (0–0) 0	90 (90–90) 90	0 (0–0) 0	91 (90–91) 91	15	5
**VENTRICULAR ARRHYTHMIA**	0 (0–0) 0	88 (74–90) 90	0 (0–0) 0	91 (82–91) 91	29	7
**VISION BLURRED**	0 (0–0) 0	88 (83–89) 89	0 (0–0) 0	82 (73–82) 82	0	22
**VISUAL IMPAIRMENT**	33 (0–50) 0	90 (89–100) 100	50 (0–100) 0	89 (80–91) 91	38	15
**WEIGHT INCREASED**	0 (0–0) 0	79 (70–88) 90	0 (0–0) 0	82 (73–91) 82	43	18

## Discussion

In this study we developed a preliminary approach to predict 135 adverse events of high priority to regulatory review using post-market safety data from marketed drugs that have the same activity at one or more of the same targets. We identified 53 adverse events that were well-predicted with this approach and chose to use a threshold which optimizes positive predictive value. These adverse events had varying sensitivity, but high specificity and negative predictive value. A model with high positive predictive value but low sensitivity will miss some true adverse events, but this was deemed acceptable for this study. In discussions about optimizing either positive predictive value or sensitivity in this study, it was deemed more important to identify adverse events that are most likely to be true and save time and effort sifting through false positives. In practice, a balance between sensitivity and positive predictive value would likely be optimal in conjunction with a manual review of predictions.

Adverse event predictions based on molecular targets have multiple applications. We may be able to identify difficult to observe events that are not commonly seen in clinical trials to statistical significance. Predicted adverse events may be able to augment post-marketing surveillance activities by providing a list of adverse events to monitor. If an adverse event is discovered during pre-market evaluation or post-market utilization, examination of other drugs with similar pharmacologic mechanism and activity may help evaluate causality of the event and determine if further studies are necessary based on information from all comparators, not necessarily limited to those with the same indication. Particularly, examination of secondary targets may be useful, as this may explain the emergence of an adverse event or why a particular drug is at lower risk for adverse events traditionally labeled as a class adverse event. While the preliminary approach presented here is considered a tool for hypothesis generation, further evaluation and refinement will determine if it is useful in regulatory safety review.

The method reported in this study matches safety data based on drug activity at one or more of the same known targets. This may limit the predictive ability, as some adverse events may be idiosyncratic or be associated with unknown secondary targets, and thus the mechanisms responsible for the event have not yet been identified. Associations may still be identified, however, if overlapping structural features capture this unknown shared idiosyncratic activity. This method can be expanded to match a drug not only based on drug activity at one or more of the same targets, but also considering other features which characterize the drug activity, such as Anatomical Therapeutic Chemical (ATC) codes or binding strength (Ki). ATC codes, developed by the World Health Organization, may provide insight into drugs that are related by mechanism or therapeutic use [16]. Binding strength to targets of interest, which may be obtained from literature or databases such as the Psychoactive Drug Screening Program [17] or ChEMBL [18], may provide further classification of target similarity by identifying comparator drugs that bind to targets of interest at a similar order of magnitude. The model also does not capture drug dose that may be needed to produce the required target activity.

Fifty-six adverse events were predicted with low positive predictive value. Therefore, a positive prediction for these adverse events should be carefully reviewed by experts before reaching a conclusion. In practice, expert review augments this by assessment of FDA Adverse Event Reporting System (FAERS) reports, literature, and more recently evaluations using insurance claims and electronic health data. Reviewers may examine predictions made by this algorithm by reviewing literature and other databases to identify plausible mechanisms for the drug eliciting the reaction, or review cases in FAERS and electronic health records. More detail about evaluation of safety signals at the FDA can be found in Szarfman et al. [19]. Analysis of the poor-performing adverse events in this study identified several clinical patterns: hemorrhage (including “haemorrhage”, “haematoma”, and “rectal haemorrhage”), infection (including “cellulitis”, “fungal infection”, and “bacterial infection”), and psychiatric (including “paranoia”, “delirium”, and “hallucination”) adverse events were among the worst-performing events by positive predictive value. Many of these adverse events may be idiosyncratic or related to unknown secondary target effects, and therefore it is difficult to predict an adverse event based on the known drug targets. This study may have been limited by the known targets that are available in DrugBank, as DrugBank may not contain all known secondary targets for all drugs. To better capture adverse events that may be related to secondary drug targets, target prediction for the test drugs and comparator drugs may be incorporated to better match comparator drugs to test drugs. DrugBank contains limited target predictions, so another source would be used.

This study had several limitations. First, the current version of Embase only allows users to extract manually curated adverse events by date for one drug at a time, which makes this process time-intensive for a large set of test drugs and their comparators and thus limited the number of drugs used in this study. We tried to address this limitation by using a probabilistic approach of performance evaluation using bootstrapping. Creating a tool to automate extraction of these adverse events may alleviate the manual burden. Additionally, text-mining FDA labels for adverse events is most accurate when used on a structured document, and thus we elected to use test drugs that had labels available in SPL format. While an assessment of the text-mining for 20 labels showed positive predictive value, sensitivity, and F-score at approximately 90% (unpublished data, Racz et al., 2018), we anticipate larger text-mining errors. This assessment identified patterns in the text-mining algorithm that may lead to errors, and the query is currently being updated to improve performance. Finally, several adverse events were not observed or observed with low prevalence in the test drug set. Further analysis of these adverse events identified some events that may be associated with targets that were not substantially analyzed. This includes events such as “respiratory depression”, which is particularly associated with drugs such as benzodiazepines and opioids and their related receptors [20], and “hypokinesia”, which may be associated with dopamine receptors [21]. Other adverse events, such as “anaphylactoid reaction” and “apnea”, may be reported interchangeably with other MedDRA Preferred Terms, such as “anaphylactic reaction” and “sleep apnea”, respectively; therefore, these terms may be reported in lower frequency. To better capture this, we may consider alternative groupings or adding additional terms to complete a mechanistically-related grouping.

## Conclusions

This classifier algorithm predicts significant adverse events that are of high priority for regulatory monitoring, some of which may be difficult to observe in clinical trials. The prediction algorithm uses evidence of adverse events available through FDA product labels and scientific literature for drugs that have the same activity at one or more of the same targets along with structural and target similarities and the duration of post-market experience. For this study, we prioritized achieving high positive predictive value for the adverse event prediction. The model achieved high positive predictive value on 53 out of 135 adverse events, including several adverse events with well-characterized target relationships. We found that 32% of the model predicted safety label changes were FDA-issued within four to nine years after approval.

## Methods

### Selection of adverse events for evaluation

This methodology predicts 135 adverse events identified by FDA medical experts and reviewers to be of high priority to regulatory review and the pharmacovigilance efforts of the Office of Surveillance and Epidemiology. High priority was determined by FDA pharmacovigilance experts as events that are serious, may be life-threatening or debilitating, or represent frequent events that result in the need for safety label changes. These 135 adverse events were derived using 167 MedDRA Preferred Terms, grouped by mechanistic similarity according to FDA medical experts. For example, “pancreatitis” and “pancreatitis acute” are mechanistically similar and may be reported interchangeably, thus they were captured as one adverse event, “pancreatitis”. The 135 adverse events and the 167 MedDRA Preferred Terms used to define them are listed in Table 4. MedDRA is the Medical Dictionary for Regulatory Activities and is the international medical terminology developed under the auspices of the International Council for Harmonization of Technical Requirements for Pharmaceuticals for Human Use [22]. MedDRA Preferred Terms are medical concepts for symptoms, signs, diagnoses, indications, investigations, procedures, and medical, social, or family history. The FDA Adverse Event Reporting System (FAERS) currently codes reported adverse events as MedDRA Preferred Terms, and all terms from other sources were converted to MedDRA Preferred Terms as described below.

**Table 4:** Adverse events defined using MedDRA Preferred Terms. The bolded MedDRA Preferred Term is used to name the adverse event, while all MedDRA Preferred Terms grouped together were used to define that adverse event

Adverse Event			
ACCIDENT	CONFUSIONAL STATE	HALLUCINATION	PULMONARY OEDEMA
ACUTE KIDNEY INJURY	CONJUNCTIVITIS	HEPATIC FAILURE	RECTAL HAEMORRHAGE
AGGRESSION	CROHN’S DISEASE	HEPATIC NECROSIS	RENAL FAILURE
AGRANULOCYTOSIS	DEAFNESS	HEPATITIS	RENAL IMPAIRMENT
AMNESIA	DEEP VEIN THROMBOSIS	HOSTILITY	RESPIRATORY DEPRESSION
ANAEMIA	DELIRIUM	HYPERSENSITIVITY	RHABDOMYOLYSIS
ANAPHYLACTOID REACTION	DELUSION	HYPERTENSION	ROAD TRAFFIC ACCIDENT
ANGINA PECTORIS	DERMATITIS BULLOUS	HYPOGLYCAEMIA	SEROTONIN SYNDROME
ANGIOEDEMA	DERMATITIS EXFOLIATIVE	ABASIA	SKIN ULCER
APLASTIC ANAEMIA	DIABETES MELLITUS	IMPAIRED HEALING	SLEEP DISORDER
APNOEA	DIPLOPIA	INFECTION	STOMATITIS
ARRHYTHMIA	DISORIENTATION	INJURY	SUDDEN DEATH
ATRIOVENTRICULAR BLOCK	DYSGEUSIA	INSOMNIA	TACHYCARDIA
AZOTAEMIA	EMBOLISM	INTERSTITIAL LUNG DISEASE	THROMBOCYTOPENIA
BACTERIAL INFECTION	EOSINOPHILIA	LARYNGEAL OEDEMA	THROMBOPHLEBITIS
BLINDNESS	ERYTHEMA MULTIFORME	LEUKOPENIA	THROMBOSIS
BONE MARROW FAILURE	COLITIS ULCERATIVE	MEMORY IMPAIRMENT	TINNITUS
BRADYCARDIA	FALL	MYOPATHY	TOXIC EPIDERMAL NECROLYSIS
BRONCHITIS	FEBRILE NEUTROPENIA	MYOSITIS	ULCER
CANDIDA INFECTION	FRACTURE	NEUTROPENIA	VISION BLURRED
CARDIAC ARREST	FUNGAL INFECTION	OLIGURIA	URINARY TRACT INFECTION
CARDIOMYOPATHY	GLAUCOMA	PANCYTOPENIA	URTICARIA
CATARACT	GRANULOCYTOPENIA	PARALYSIS	VAGINAL HAEMORRHAGE
CELLULITIS	HAEMATOMA	PARANOIA	VASCULITIS
GASTROINTESTINAL HAEMORRHAGE	NEUROLEPTIC MALIGNANT SYNDROME	PHOTOSENSITIVITY REACTION	UPPER RESPIRATORY TRACT INFECTION
CHOLESTASIS	HAEMOLYTIC ANAEMIA	PNEUMONIA	WEIGHT INCREASED
COAGULOPATHY	HAEMORRHAGE	PROTEINURIA	**SEIZURE**, EPILEPSY
COLITIS	CEREBRAL INFARCTION	PULMONARY EMBOLISM	**SEPSIS**, SEPTIC SHOCK
STEVENS-JOHNSON SYNDROME	EXTRAPYRAMIDAL DISORDER	**JAUNDICE**, JAUNDICE CHOLESTATIC	RESPIRATORY ARREST, **RESPIRATORY FAILURE**
**ANAPHYLACTIC REACTION**, ANAPHYLACTIC SHOCK	**NEUROPATHY PERIPHERAL**, PARAESTHESIA	**HYPERGLYCAEMIA**, BLOOD GLUCOSE INCREASED	OEDEMA PERIPHERAL, **OEDEMA**
CARDIAC FAILURE CONGESTIVE, **CARDIAC FAILURE**	TORSADE DE POINTES, **ELECTROCARDIOGRAM QT PROLONGED**	**MYOCARDIAL INFARCTION**, ACUTE MYOCARDIAL INFARCTION	ATRIAL FIBRILLATION, **SUPRAVENTRICULAR TACHYCARDIA**
**CEREBRAL HAEMORRHAGE**, HAEMORRHAGE INTRACRANIAL, CEREBELLAR HAEMORRHAGE	**SUICIDAL BEHAVIOUR**, COMPLETED SUICIDE, SUICIDE ATTEMPT, SUICIDAL IDEATION	VENTRICULAR FIBRILLATION, **VENTRICULAR ARRHYTHMIA**, VENTRICULAR EXTRASYSTOLES, VENTRICULAR TACHYCARDIA	**HYPERKINESIA**, TARDIVE DYSKINESIA, DYSKINESIA, AKATHISIA, DYSTONIA, HYPERTONIA
**HYPERCHOLESTEROLAEMIA**, HYPERLIPIDAEMIA	**HEPATOTOXICITY**, LIVER INJURY	**PANCREATITIS**, PANCREATITIS ACUTE	**GASTRIC ULCER**, PEPTIC ULCER
**CEREBROVASCULAR ACCIDENT**, TRANSIENT ISCHAEMIC ATTACK	VISUAL ACUITY REDUCED, **VISUAL IMPAIRMENT**, VISUAL FIELD DEFECT	DRUG REACTION WITH EOSINOPHILIA AND SYSTEMIC SYMPTOMS	

### Dataset

#### Drug set selection

##### Selection of test drugs

Fifty-four drugs approved by FDA between 2008 and 2013 were chosen for this analysis. Analyses were based on available Structured Product Labeling for products and required both an original label and a subsequent version of the label for this assessment. As Structured Product Labeling began in 2006, 2008 was selected to allow time for the requirement to be adequately implemented. The year 2013 was selected as the upper bound to allow at least four years of post-market experience to 2017, which is the median time for a regulatory action on a safety event (e.g. updating a drug label) [23]. Of the drugs approved between 2008 and 2013, drugs were included as long as there was at least one other U.S. marketed drug with the same pharmacological activity at one or more of the same known targets. Additional inclusion criteria were systemic exposure (e.g. not ophthalmic only) and multiple doses (i.e. drugs with single dose administration were excluded) due to an increased likelihood of multiple and significant adverse events.

##### Selection of comparator drugs

Comparator drugs, defined as drugs that have the same activity (i.e. agonist or antagonist) at one or more of the same targets as the test drug, were chosen using DrugBank [24]. Test and comparator drug targets were identified if the drug had “pharmacological action” at the target (i.e. the column “pharmacological action” in DrugBank must read “yes” as opposed to “no” or “unknown”) and must have a defined action column in DrugBank (i.e. “antagonist” or “agonist”) at the target. Additionally, the comparator drugs must have been approved in the United States and thus have an FDA product label available.

### Features for classifier algorithm

#### Adverse Events from FDA drug labels

Adverse events were obtained from two versions of the test drug label: the originally-approved FDA product label (between 2008 and 2013) and the drug label as of 2017. The adverse events from the 2017 FDA product label were text-mined using Linguamatics I2E (OnDemand Release, Linguamatics Limited, Cambridge, United Kingdom). Adverse events were extracted as MedDRA Preferred Terms from the Boxed Warnings, Warnings and Precautions, and Adverse Reactions sections. The adverse events from the original product label were manually extracted and translated to MedDRA Preferred Terms by a medical expert from the Boxed Warnings, Warnings and Precautions, and Adverse Reactions sections. Manual curation was employed as Linguamatics OnDemand text-mines the current product label only.

Comparator drug adverse events were text-mined using Linguamatics I2E (Enterprise Release, Linguamatics Limited, Cambridge, United Kingdom). Adverse events were extracted as MedDRA Preferred Terms from Boxed Warnings, Warnings and Precautions, and Adverse Reactions sections. For each comparator drug, the FDA product label in use at the time of the respective test drug approval was used as the source for text-mining (e.g.: if a test drug was approved on November 1, 2010, the comparator drug labels that were in use on November 1, 2010 were mined).

For each drug label and adverse event, the presence or absence of a MedDRA Preferred Term was indicated by “1” or “0”, respectively. The classifiers were trained on and performance was analyzed using test drug label data from 2017. To assess the algorithm’s ability to predict future safety label changes at the approval date (described in detail in “Classifier” below), the difference between drug label data from 2017 and the label at approval (2008–2013) was used.

#### Adverse events from scientific literature

Adverse events from scientific literature were mined using Embase Biomedical Database (Elsevier B. V, Amsterdam, The Netherlands), a biomedical database covering journals and conference abstracts [25]. A team of Embase indexers manually curate all adverse events from all full-text articles and associate each adverse event with the related drug. These drugs and adverse events are documented in Emtree terms, Elsevier’s controlled terminology. Therefore, each drug in Embase has hundreds to thousands of adverse events associated with it, and each adverse event-drug association has a curated reference. Adverse events reported for all comparator drugs before their respective test drug’s approval date were searched for in Embase. The list of adverse events documented by Elsevier as Emtree terms for each comparator drug was exported and manually matched to MedDRA Preferred Terms.

#### Comparator drug duration in market

Comparator time in market was included as a feature. The longer a drug has been marketed, the more adverse events, particularly difficult to observe adverse events, are identified and evaluated for labeling. The duration in market for comparator drugs was determined from the Orange Book [26]. Drugs that were approved before 1982 have an approval date listed as “Approved Prior to Jan 1, 1982”; the duration in market for these drugs was imputed to be 36 years (1982 to 2017).

#### Structural similarity

Structural similarity was included as a feature as it was hypothesized that the more structurally similar a comparator drug was to a test drug, the more likely they were to share pharmacology, including unknown secondary pharmacology that was not included in this analysis and may contribute to similar idiosyncratic reactions. Structural similarities of each test drug to its respective comparator drugs were determined using Tanimoto scores. Simplified Molecular Input Line Entry System (SMILES) structures for all test and comparator drugs were imported into the Tanimoto Matrix workflow in the KNIME Analytics Platform (version 3.3.2) [27]. Structures were then converted to MACCS 166-bit fingerprints, and structural similarity between the test drug and the respective comparator drug was determined. For biologics where similarity score was not available, − 1 was imputed as Tanimoto score.

#### Target similarity

Target similarity, or how closely the target profile of each comparator aligned with that of the test drug, was included as a feature as it was hypothesized that the more targets a comparator shares with a test drug, the more likely it is that a comparator and test drug share adverse events. The set of known pharmacological targets for each test drug and corresponding comparator drugs was extracted from DrugBank [24]. Target similarities of each test drug with its comparator drugs were determined using target-based cosine similarity scores. A trivalent drug-by-target matrix was then constructed such that for each drug-target pair an entry of “1” indicates drug-target activation, an entry of “-1” indicates drug-target inhibition, and an entry of “0” indicates no pharmacological activity. Cosine similarities the test drug has with its comparator drugs were then computed as follows:

$$ cosine\left(\left[ Test\ Drug\right],\left[ Comparator\ Drug\right]\right)=\frac{\left[ Test\ Drug\right]\bullet \left[ Comparator\ Drug\right]}{\left\Vert \left[ Test\ Drug\right]\right\Vert \left\Vert \left[ Comparator\ Drug\right]\right\Vert } $$

### Classifier

**Fig. 4:**
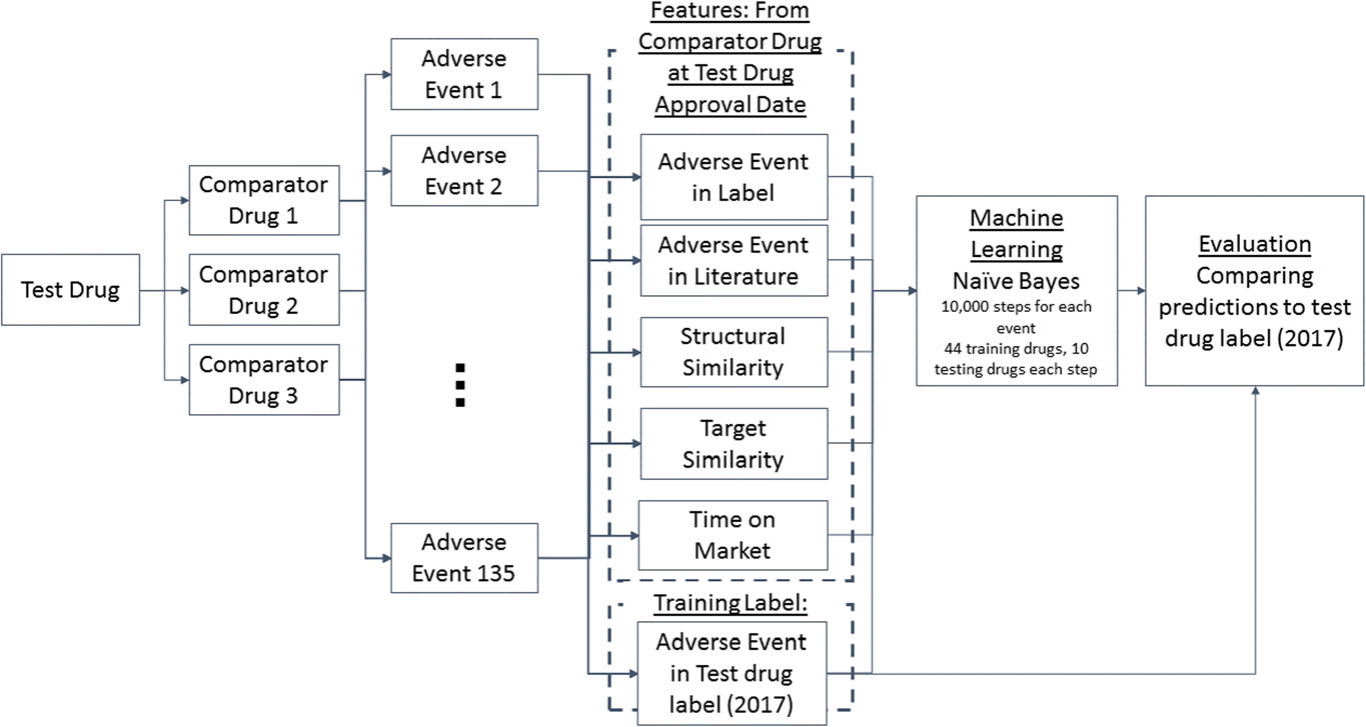
Flow diagram of experimental methods

Five features were defined for each comparator-test drug -adverse event association: 1) presence or absence of an adverse event in FDA drug label for the comparator drug; 2) presence or absence of an adverse event in scientific literature for comparator drug; 3) structural similarity between comparator drug and test drug; 4) target similarity between comparator drug and test drug; and 5) duration the comparator drug was on the market (Fig. 4), all of which are independent of each other. These features were used to train a Naïve Bayes classifier, using presence or absence of an adverse event in the 2017 FDA drug label for the test drug as the training label (see section *Adverse Events from FDA Drug Labels* for details). Given the wide range of prevalence of presence of an adverse event, we anticipated the contribution of prevelance of presence of an adverse event to model prediction would be high. Therefore a Naïve Bayes classifier was chosen in order to take into account both prior probability (i.e. prevelance of presence of an adverse event) and likelihood for presence of an adverse event. All statistical calculations were conducted in R version 3.2.2 (R Foundation for Statistical Computing, Vienna, Austria) and the Naïve Bayes classifier from package e1071 was used [28] (see supplemental materials for code).

Due to the limited number of drugs available for testing and the high dimensionality of prediction (135 adverse events), 10,000 bootstrapping steps were conducted by selecting a random set of 44 drugs to train the Naïve Bayes classifier, while leaving 10 drugs for testing at each iteration (i.e. 10,000/ ${C}_{44}^{54}$). A prediction was made by each comparator drug-test drug association for an adverse event of interest. Therefore, since a single test drug can have multiple comparator drugs, there may be multiple predictions for one test drug for each adverse event of interest. To remediate this, if the percentage of comparator drug-test drug combinations that predicted the adverse event of interest was above a predefined threshold, the adverse event was considered a positive prediction for the test drug. Performance was calculated while varying the threshold (0, 10, 30, 50, 60, 70, 90%) above which the percentage of comparator drug-test drug combinations predicted the adverse event of interest to identify the optimum threshold.

As 10,000 bootstrapping steps were performed, the most frequent value (mode), median, 25th and 75th quantiles for each of the performance metrics (sensitivity, specificity, positive predictive value and negative predictive value) were calculated to assess the predictive ability for each adverse event. Performance metric histograms for each adverse event are provided in the supplemental materials. We chose to optimize positive predictive value, as false positives may be more costly in terms of additional studies and regulatory review compared to false negatives. Adverse events with a distribution for positive predictive value that was left-skewed (defined as a mode positive predictive value > 75%) were considered well-predicted.

Leave-one-out cross validation was performed to evaluate safety label changes. Predictions were evaluated as follows:

$$ \%\mathrm{of}\ \mathrm{FDA}-\mathrm{issued}\ \mathrm{safety}\ \mathrm{label}\ \mathrm{changes}\ \mathrm{that}\ \mathrm{were}\ \mathrm{predicted}=\frac{\# of\ drug- AE\  combos\ that\ \boldsymbol{change}\boldsymbol{d}\ \boldsymbol{from}\ \boldsymbol{negative}\ \boldsymbol{to}\ \boldsymbol{positive}\  between\ approval\ and\ 2017\  that\ were\ \boldsymbol{predicted}\ \boldsymbol{positive}}{\# of\ drug- AE\  combos\ that\ \boldsymbol{change}\mathbf{d}\ \boldsymbol{from}\ \boldsymbol{negative}\ \boldsymbol{to}\ \boldsymbol{positive}\  between\ approval\ and\ 2017} $$

$$ \%\mathrm{of}\ \mathrm{predicted}\ \mathrm{safety}\ \mathrm{label}\ \mathrm{changes}\ \mathrm{that}\ \mathrm{were}\ \mathrm{also}\ \mathrm{FDA}-\mathrm{issued}=\frac{\# of\ drug- AE\  combos\ that\ \boldsymbol{changed}\ \boldsymbol{from}\ \boldsymbol{negative}\ \boldsymbol{to}\ \boldsymbol{posi}\mathbf{t}\boldsymbol{ive}\  between\ approval\ and\ 2017\  that\ were\ \boldsymbol{predicted}\ \boldsymbol{posi}\boldsymbol{tive}}{\# of\ drug- AE\  combos\ that\ were\ \boldsymbol{negative}\ \boldsymbol{at}\ \boldsymbol{approval}\  that\ were\ \boldsymbol{predicted}\ \boldsymbol{posi}\boldsymbol{tive}} $$

### Evaluation of false positive predictions

Positive predictions that were made by the Naïve Bayes classifier that were not on the respective 2017 drug label were classified as “false positives”. To further evaluate if these predictions may be early signals not yet on the label, the case count and Proportional Reporting Ratio (PRR) were identified for each drug-adverse event pair from the FDA Adverse Event Reporting System using OpenFDA [29, 30]. Data from June 30, 1989 to January 1, 2018 was used in this analysis.

## Supplementary information

**Additional file 1.** “Supplemental Materials” contains histograms of the performance for each adverse event; “Supplemental Table 1” contains all targets represented in this study.

**Additional file 2.** Contains Naïve Bayes code, files necessary to run code, and output files obtained to perform analysis described in the paper.

## Data Availability

The datasets supporting the conclusions of this article are included within the article (and its additional files).

## Abbreviations

ATC: Anatomical Therapeutic Chemical
MedDRA: Medical Dictionary for Regulatory Activities
FAERS: FDA Adverse Event Reporting System
PRR: Proportional Reporting Ratio
FDA: Food and Drug Administration
SMILES: Simplified Molecular Input Line Entry System
