# Supplementary material for: Predicting potential adverse events using safety data from marketed drugs
Source: BMC Bioinformatics. 2020 Apr 29;21:163. doi: 10.1186/s12859-020-3509-7 (PMC7191698; doi:10.1186/s12859-020-3509-7)
Supplement: Supplementary file 1 — Additional file 1. “Supplemental Materials” contains histograms of the performance for each adverse event; “Supplemental Table 1” contains all targets represented in this study. [file 12859_2020_3509_MOESM1_ESM.pdf]

Supplemental Materials

1 ACUTE KIDNEY INJURY

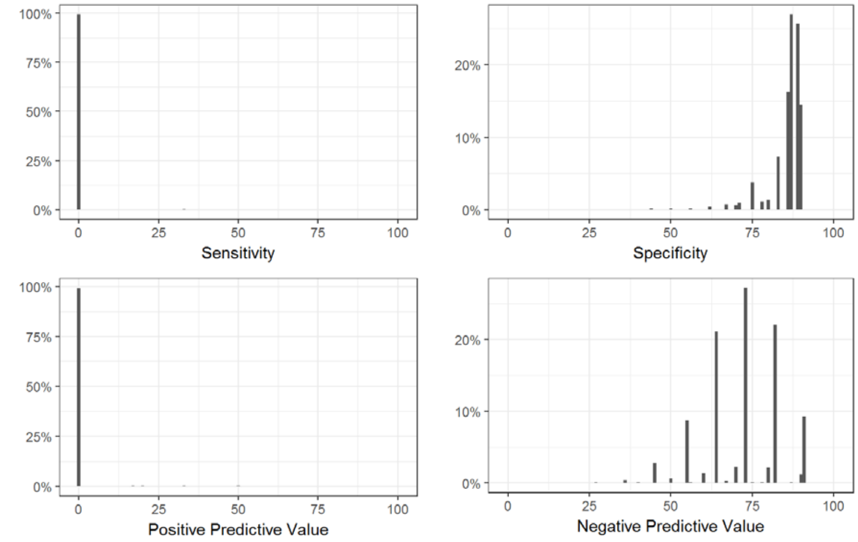

2 AGGRESSION

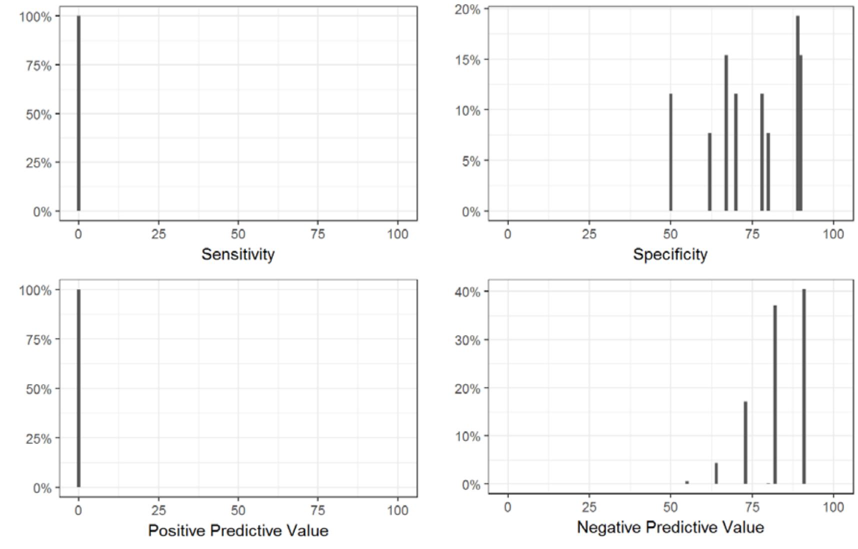

3 AGRANULOCYTOSIS

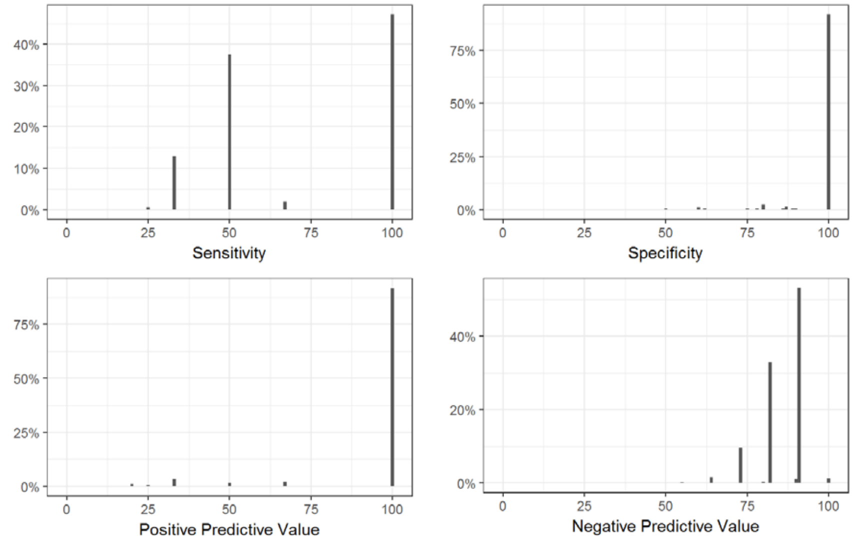

4 AMNESIA

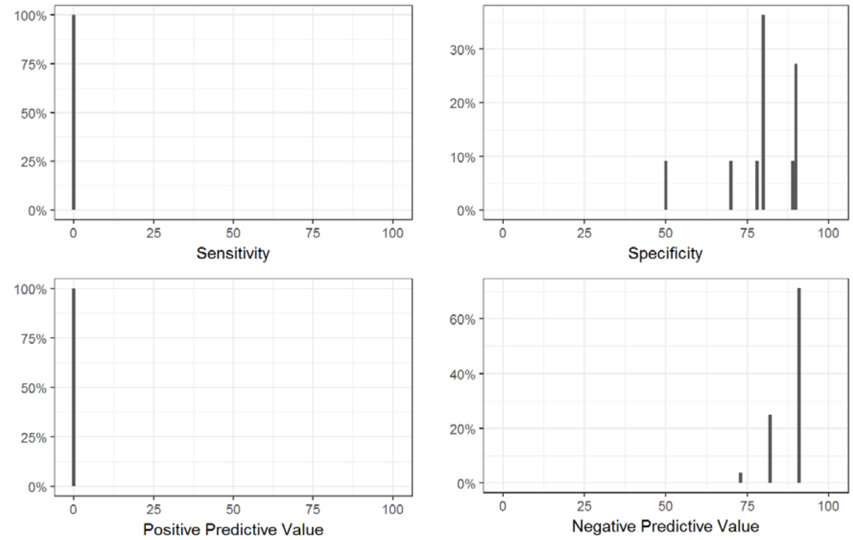

5 ANAEMIA

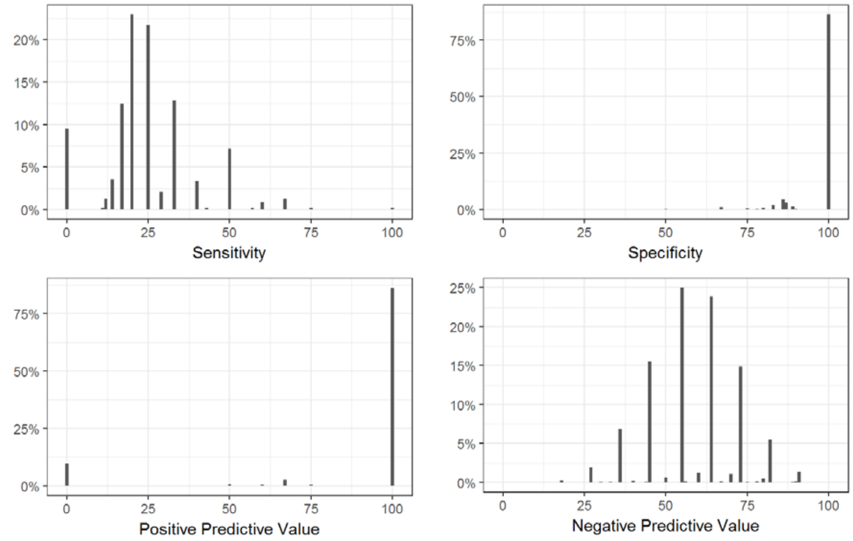

6 ANAPHYLACTIC REACTION

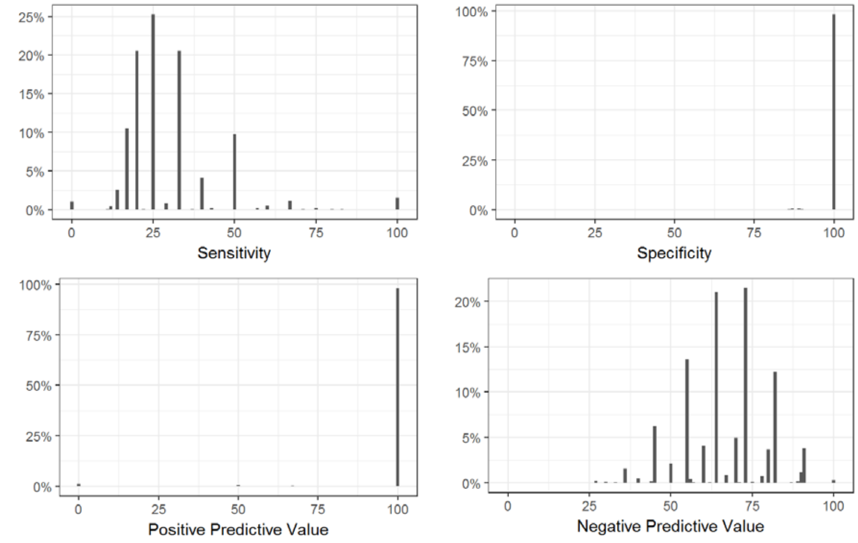

7 ANGINA PECTORIS

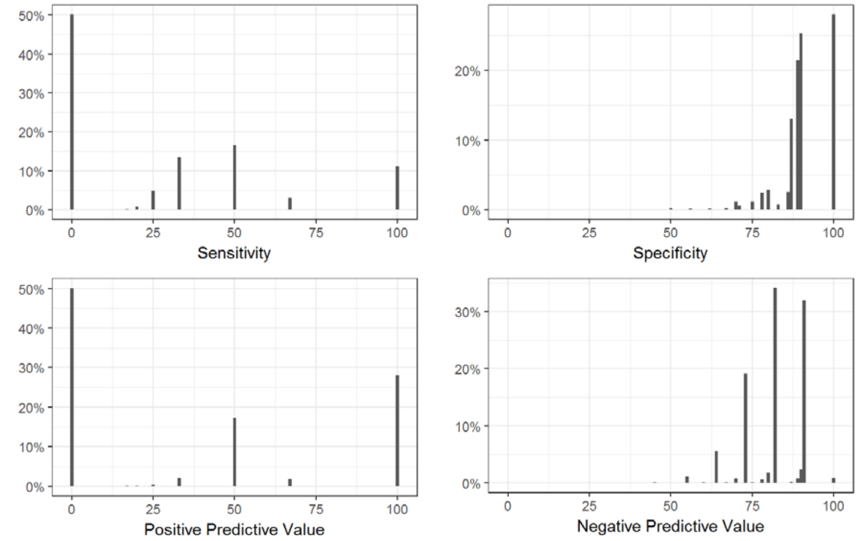

8 ANGIOEDEMA

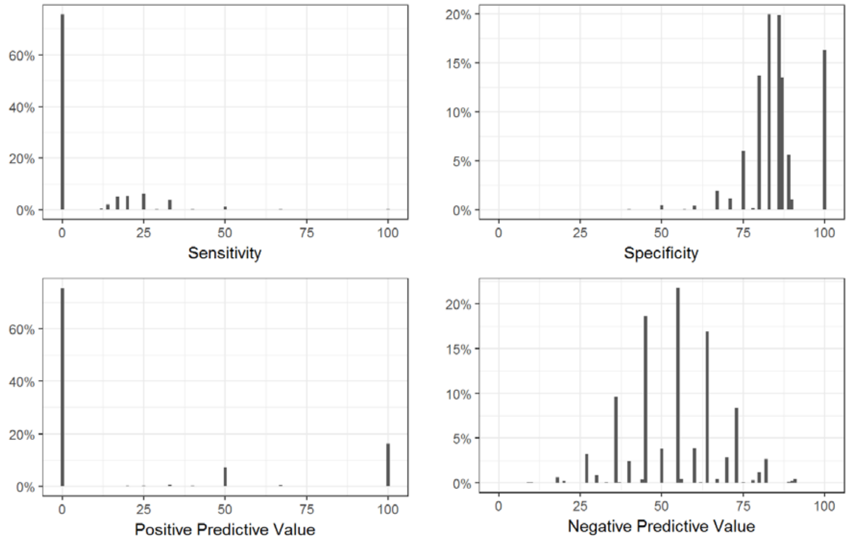

9 ARRHYTHMIA

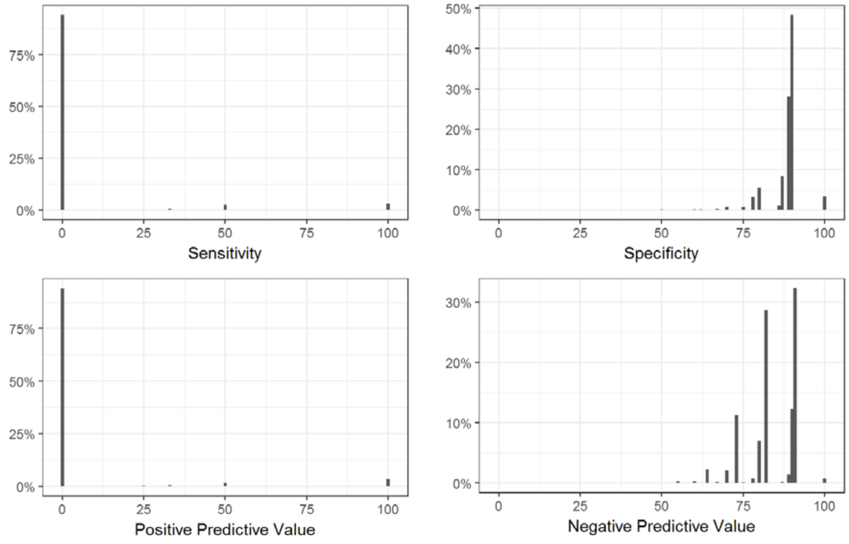

10 BACTERIAL INFECTION

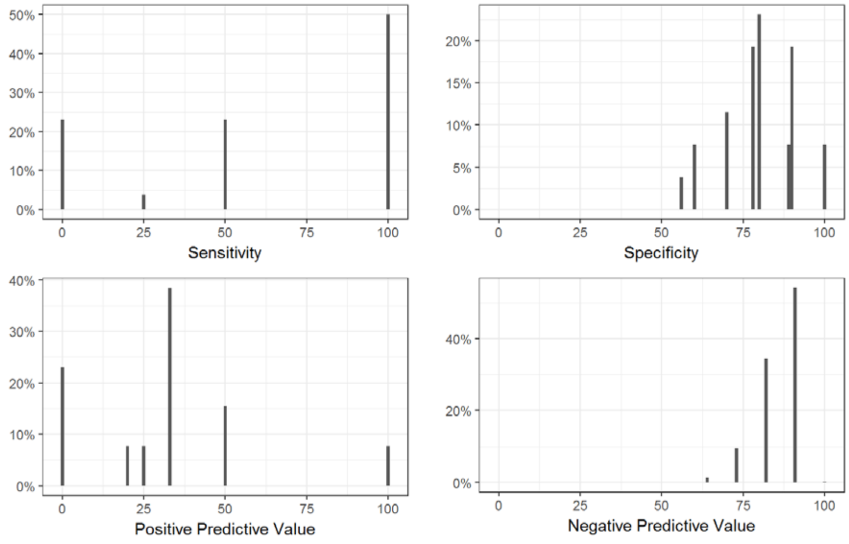

11 BLINDNESS

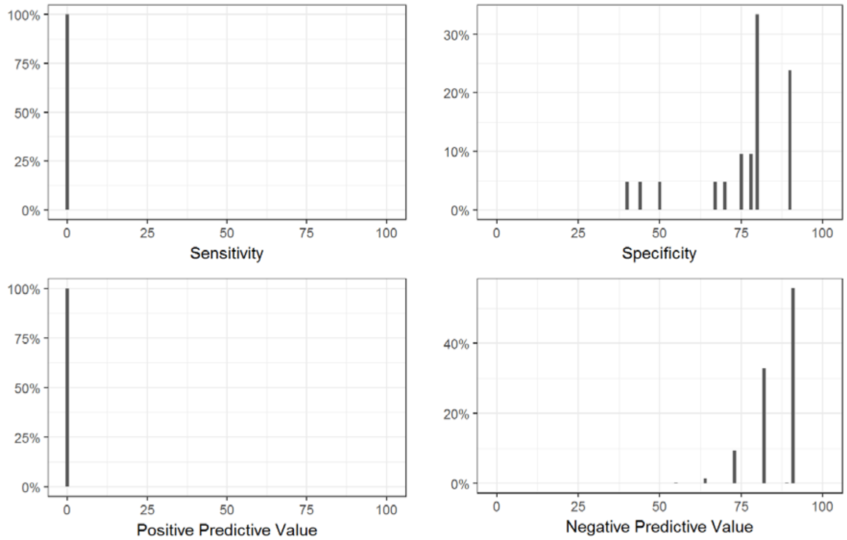

12 BONE MARROW FAILURE

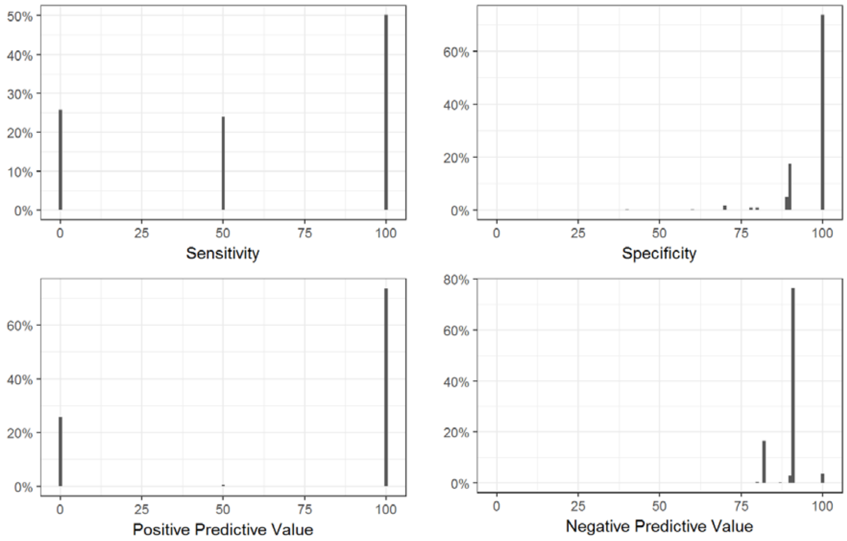

13 BRADYCARDIA

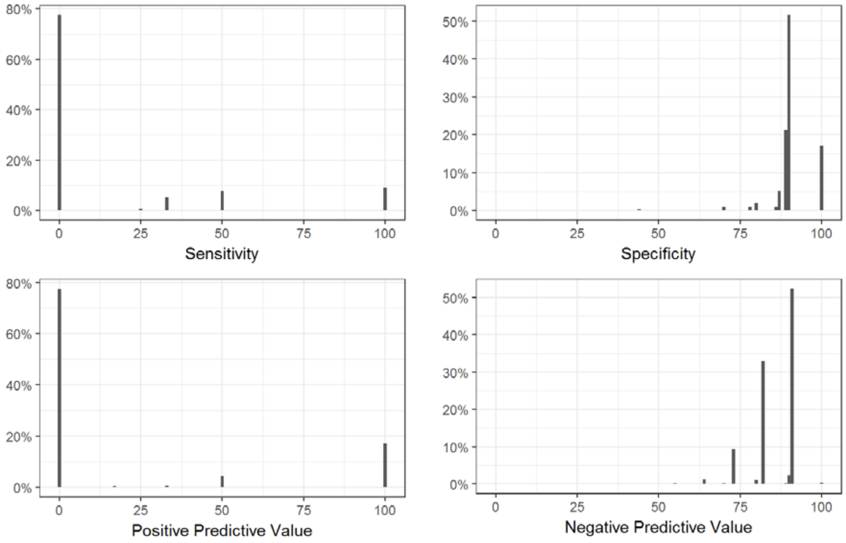

14 BRONCHITIS

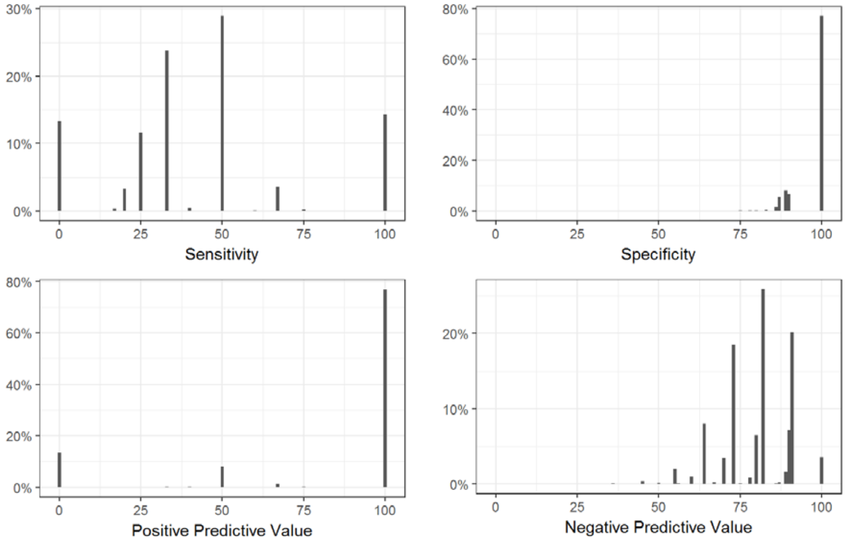

15 CANDIDA INFECTION

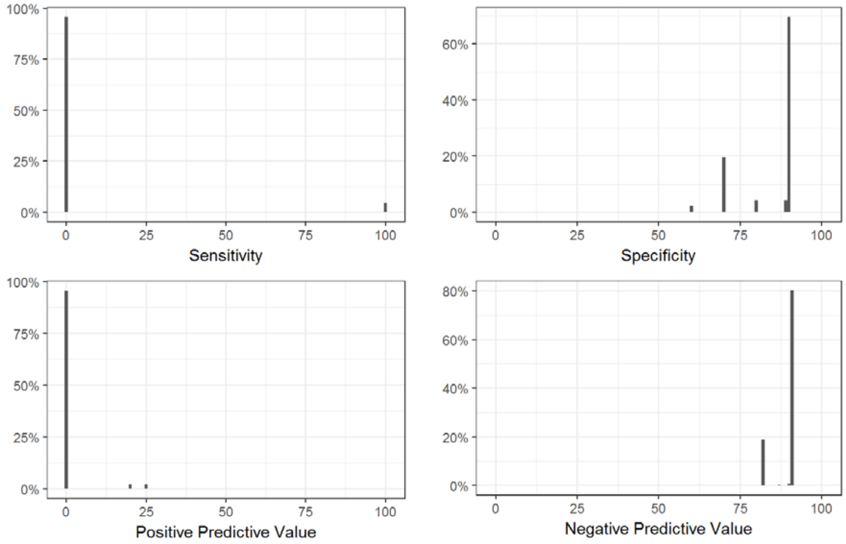

16 CARDIAC ARREST

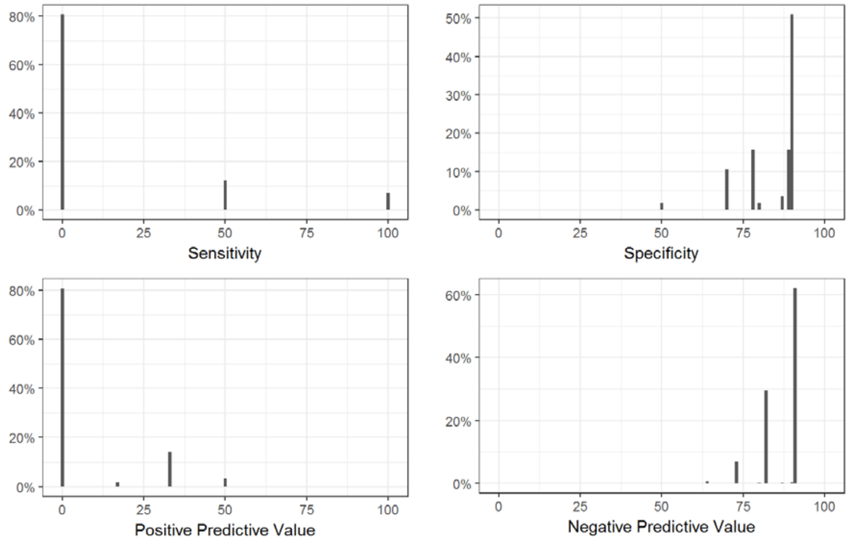

17 CARDIAC FAILURE

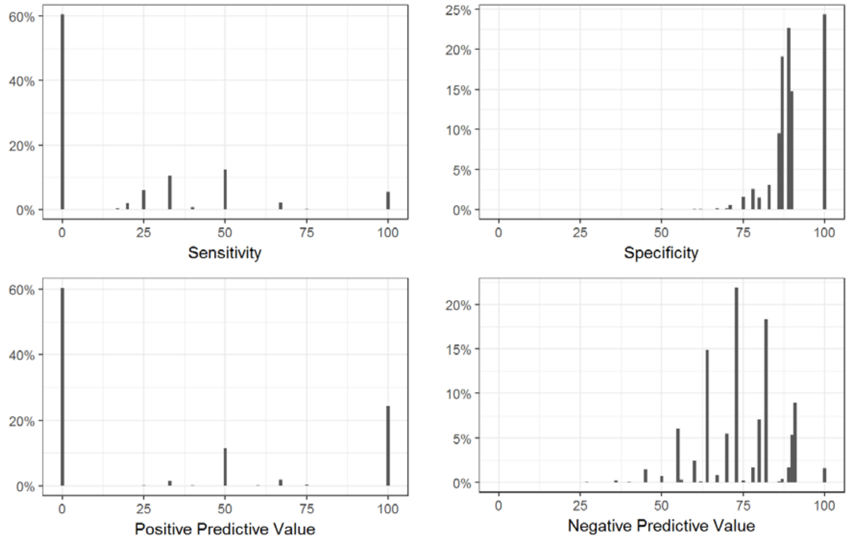

18 CATARACT

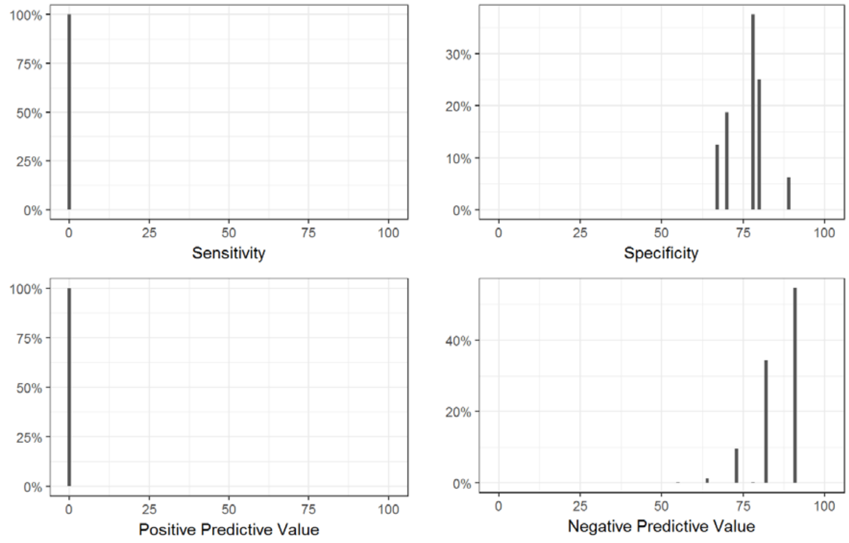

19 CELLULITIS

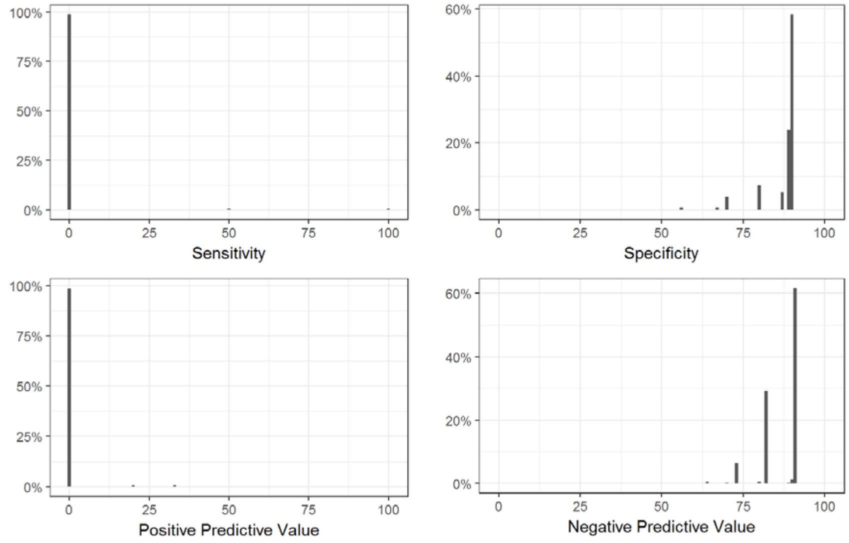

20 CEREBRAL HAEMORRHAGE

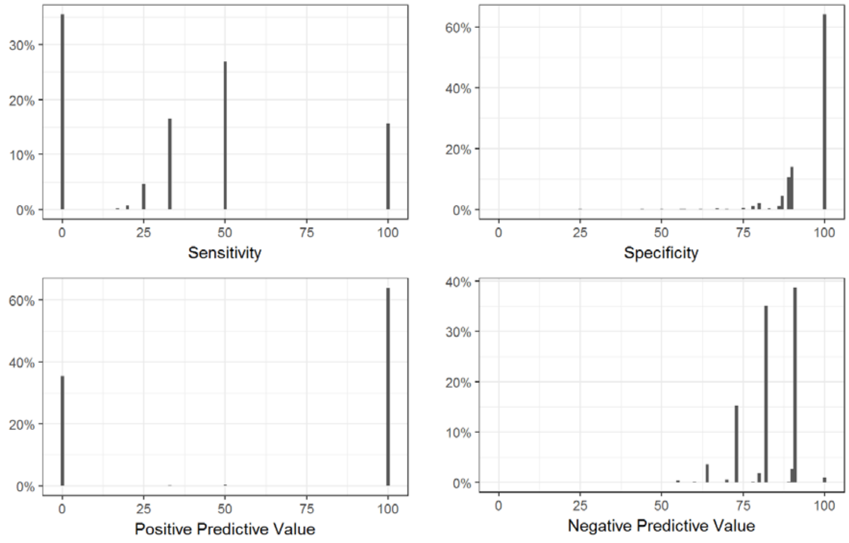

21 CEREBROVASCULAR ACCIDENT

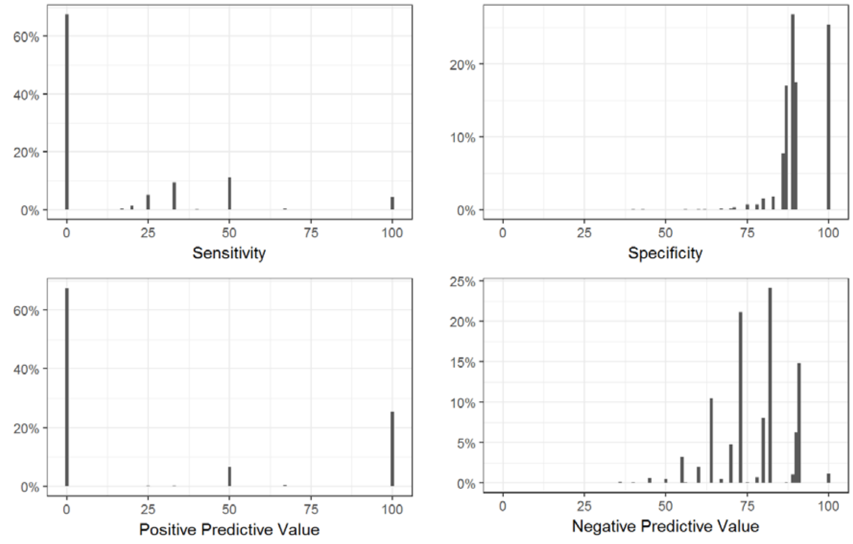

22 CHOLESTASIS

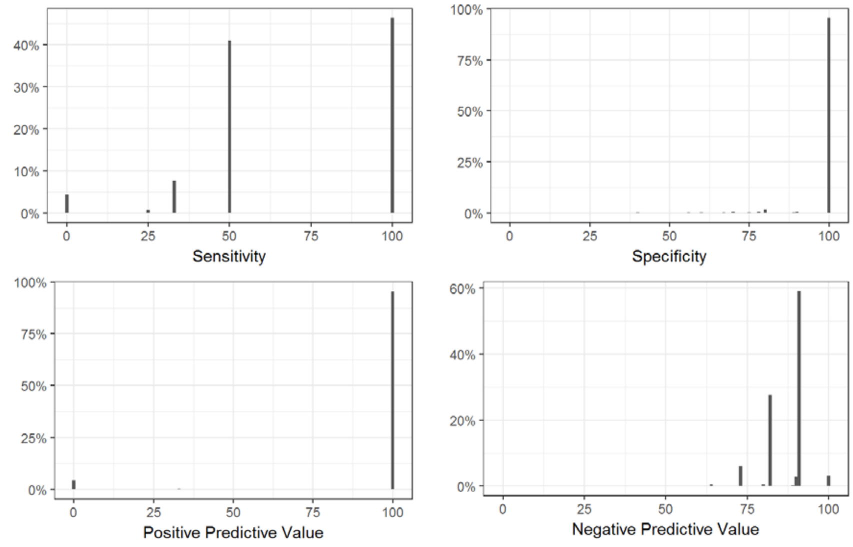

23 CONFUSIONAL STATE

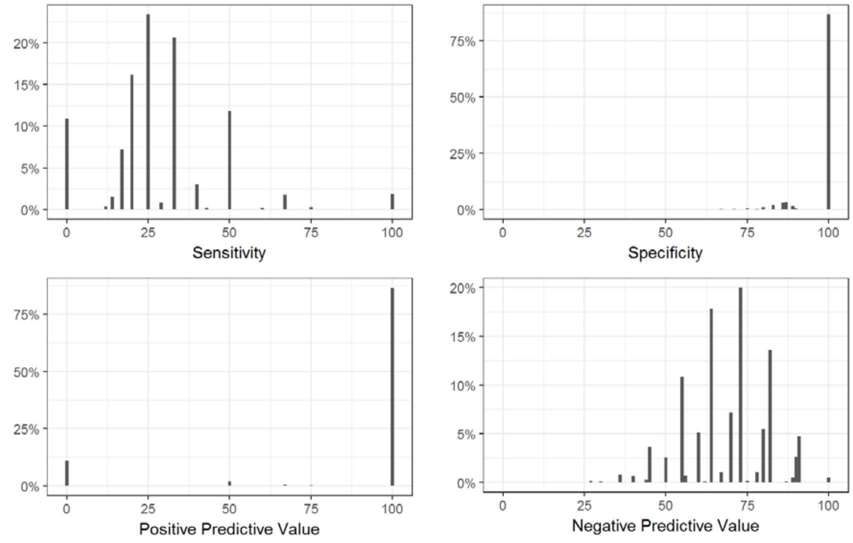

24 CONJUNCTIVITIS

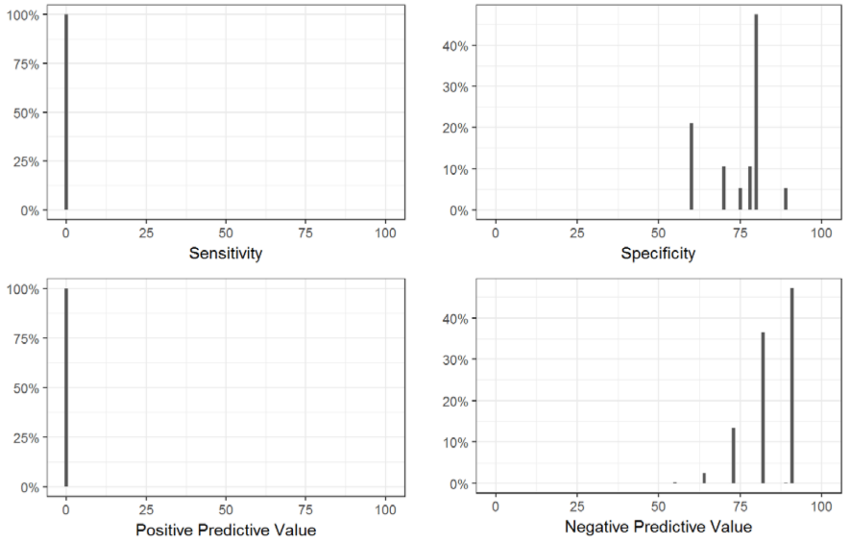

25 DEAFNESS

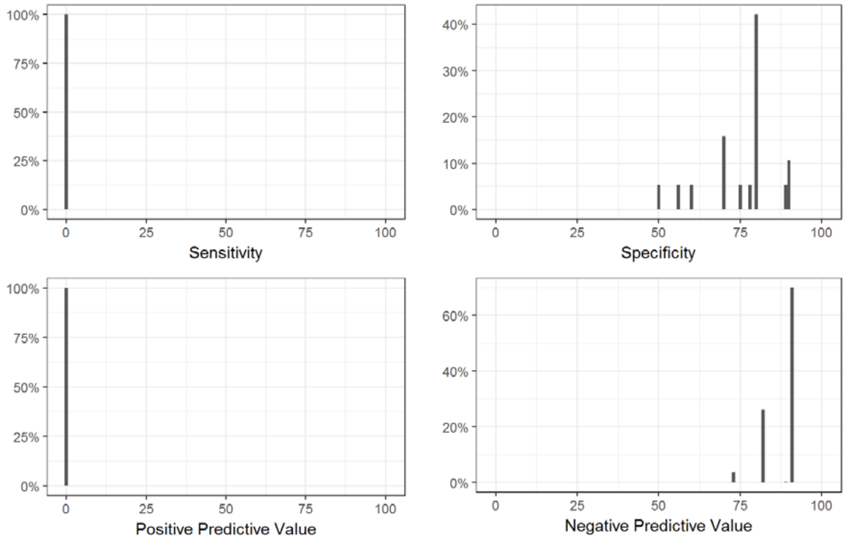

26 DEEP VEIN THROMBOSIS

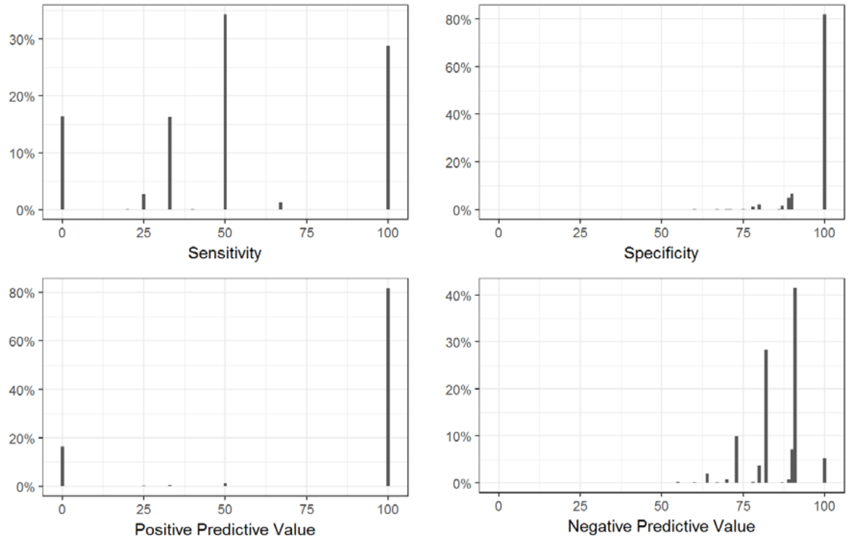

27 DELIRIUM

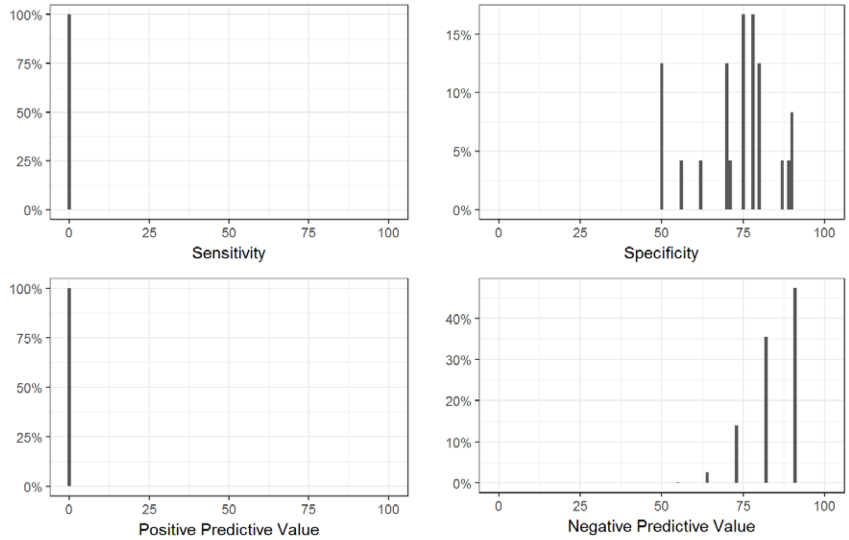

28 DELUSION

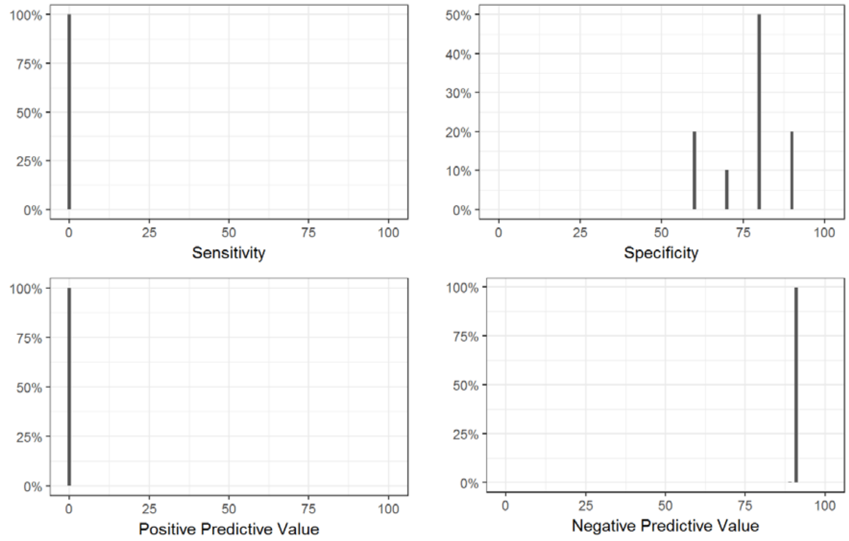

29 DIABETES MELLITUS

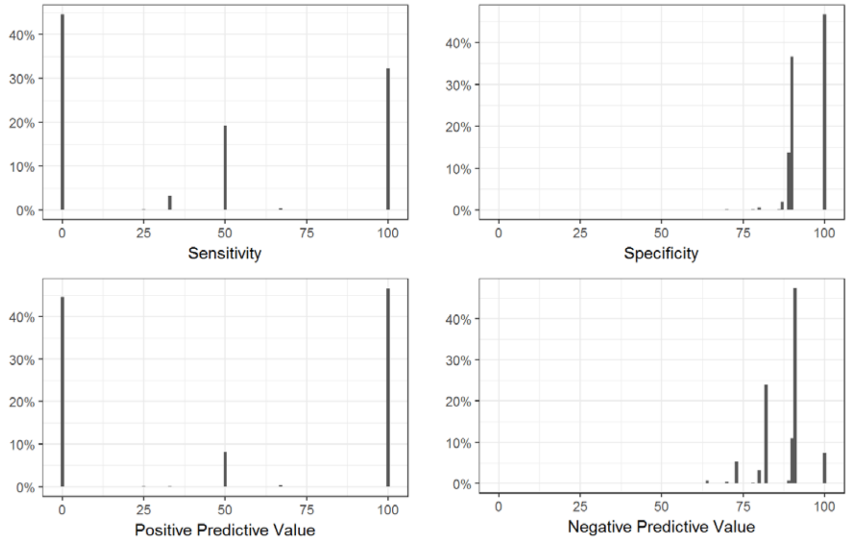

30 DIPLOPIA

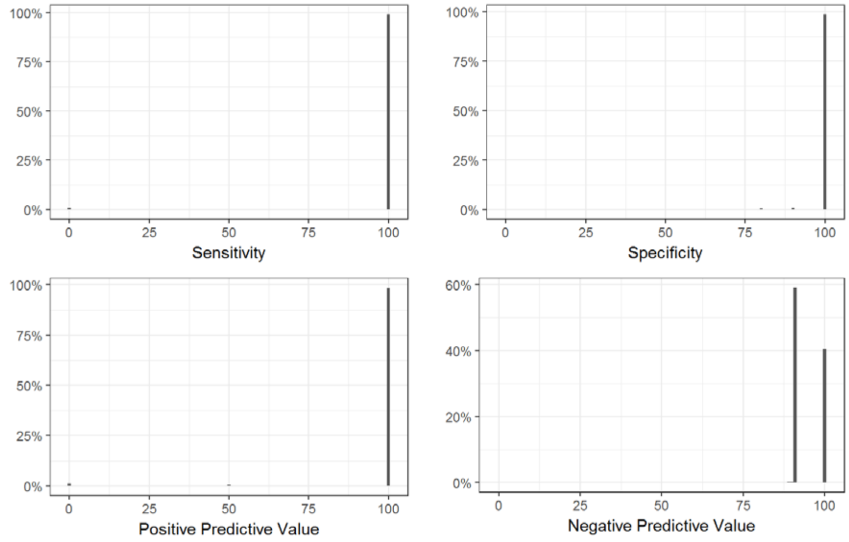

31 DISORIENTATION

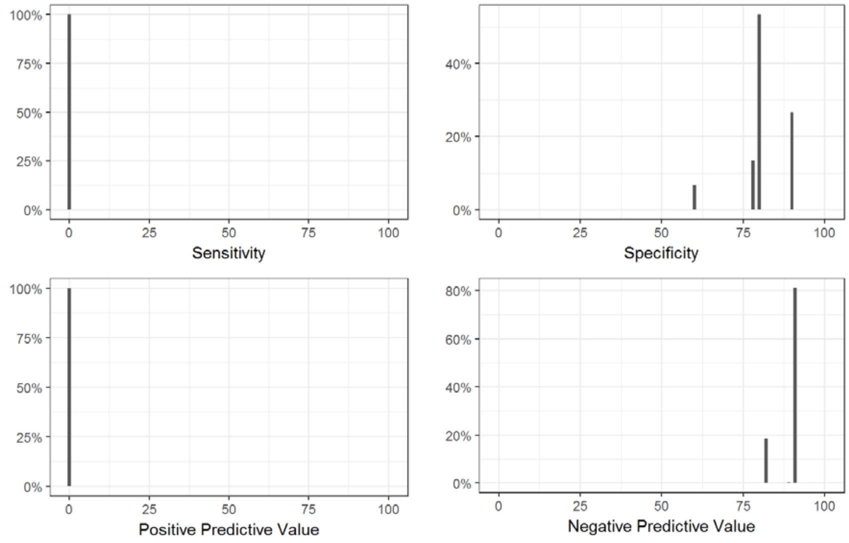

32 DRUG REACTION WITH EOSINOPHILIA AND SYSTEMIC SYMPTOMS

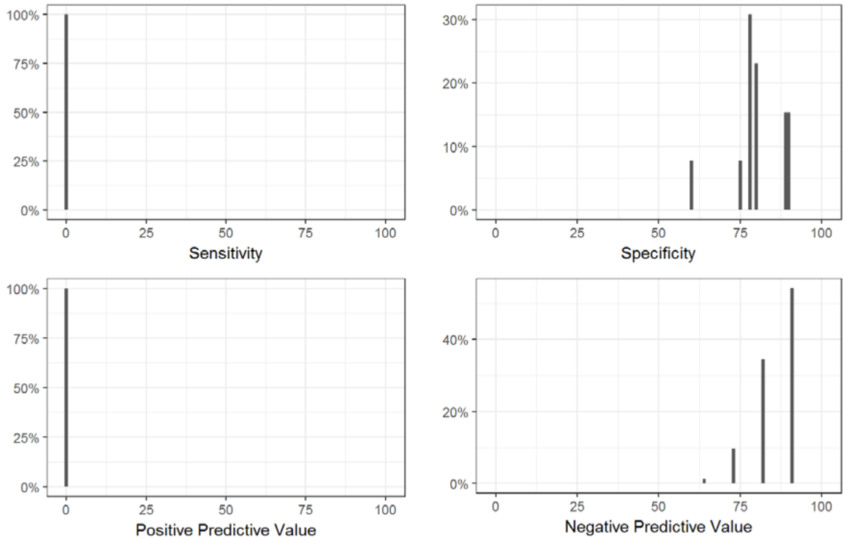

33 DYSGEUSIA

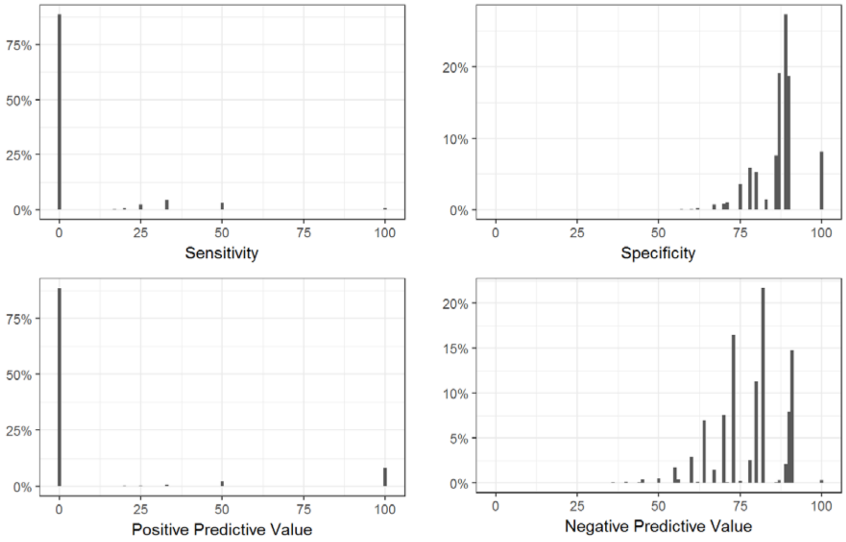

34 ELECTROCARDIOGRAM QT PROLONGED

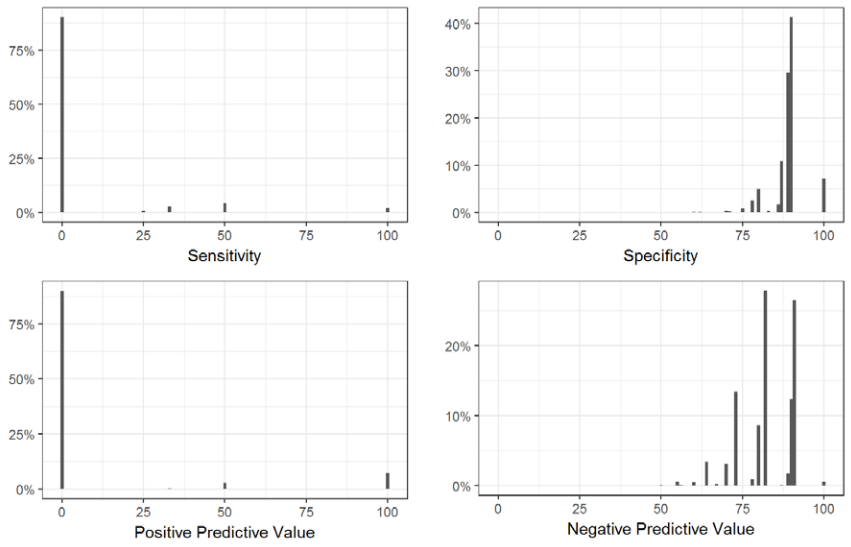

35 EMBOLISM

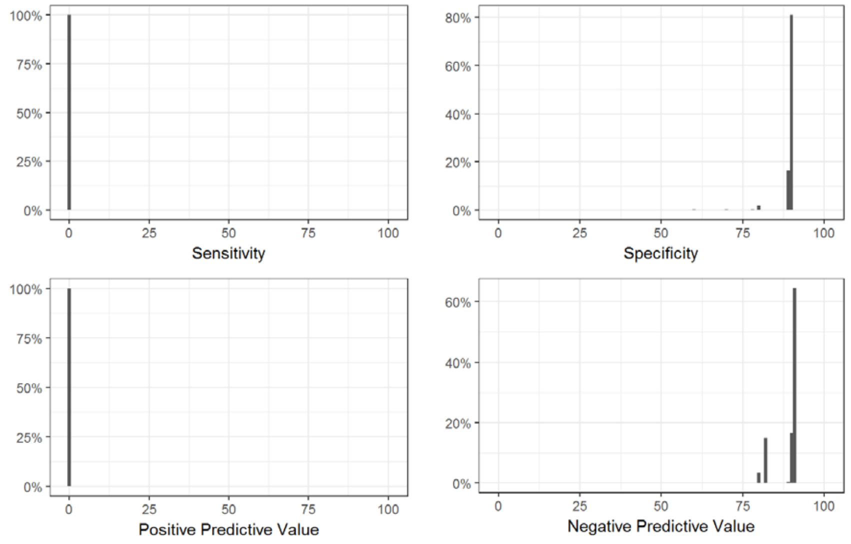

36 EOSINOPHILIA

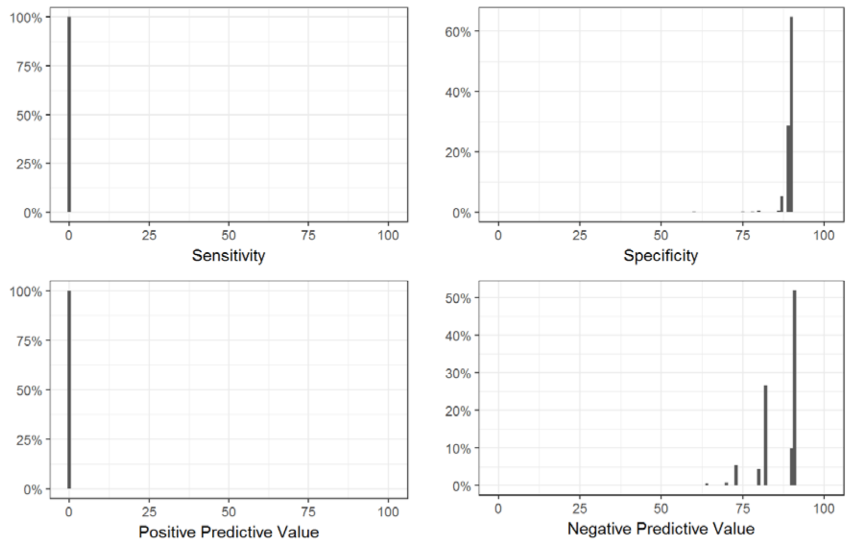

37 ERYTHEMA MULTIFORME

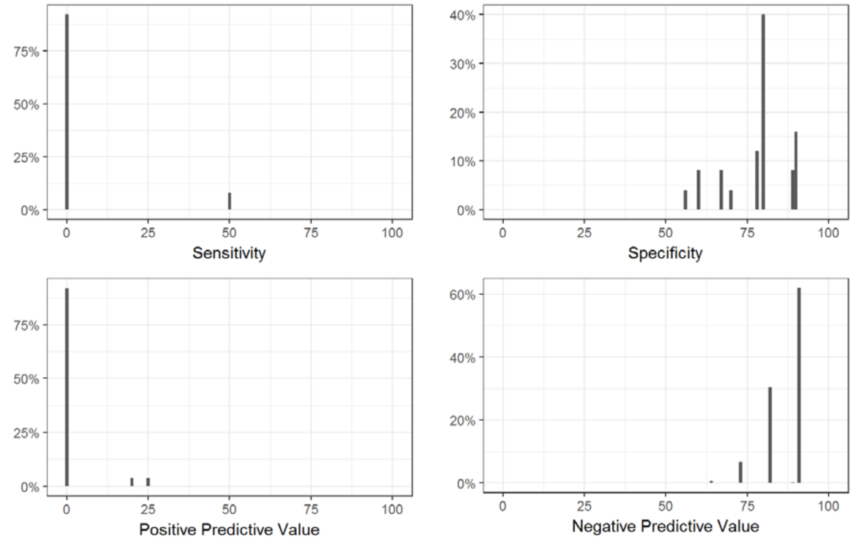

38 EXTRAPYRAMIDAL DISORDER

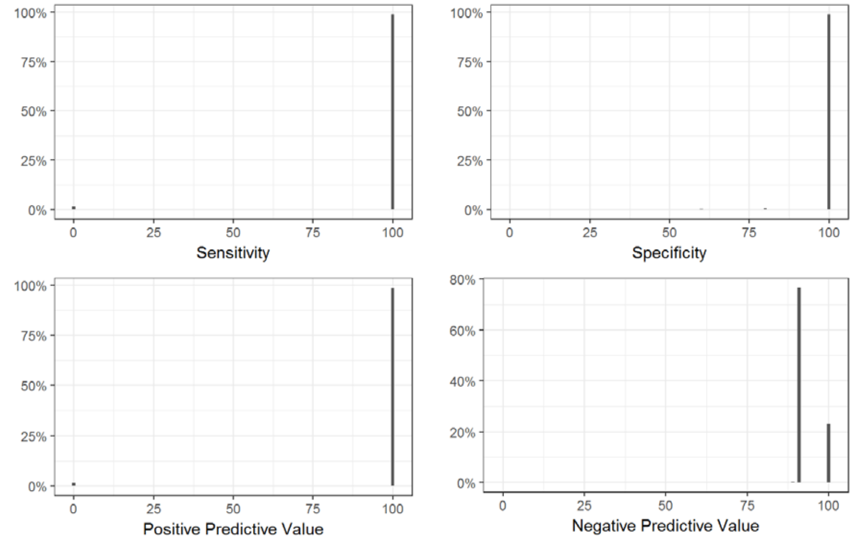

39 FALL

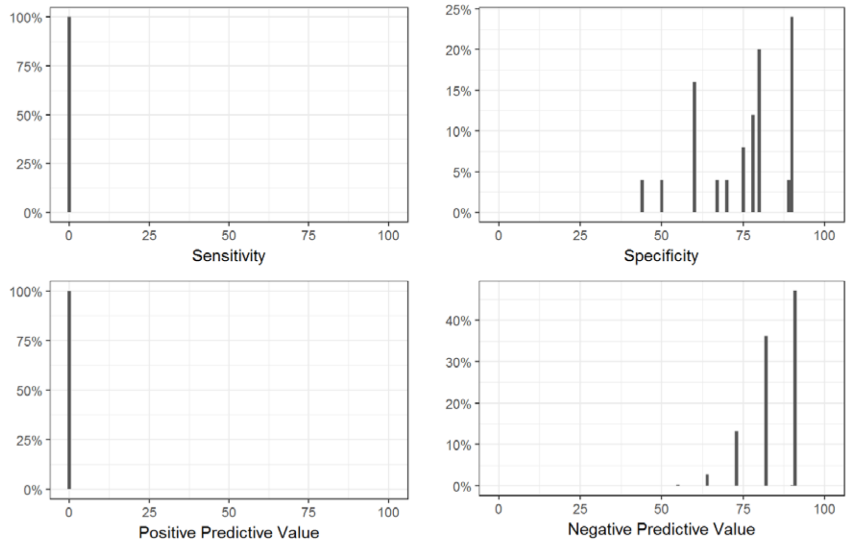

40 FEBRILE NEUTROPENIA

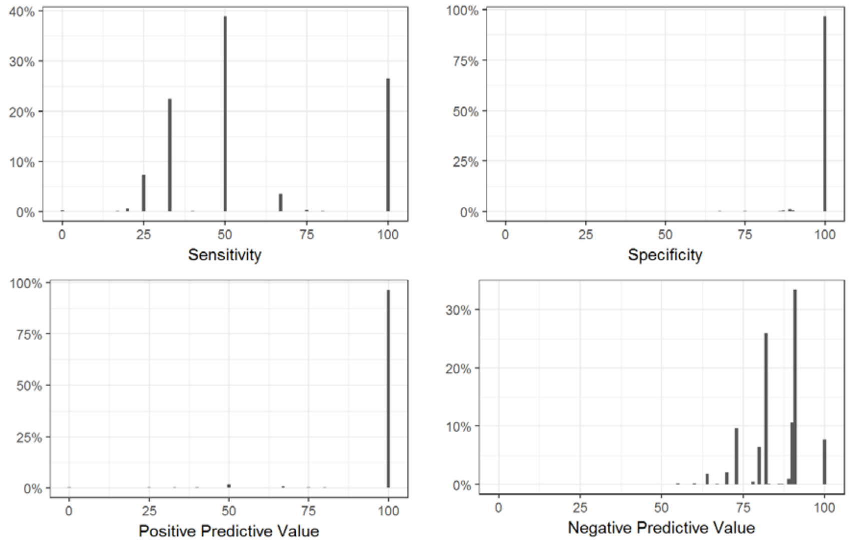

41 FRACTURE

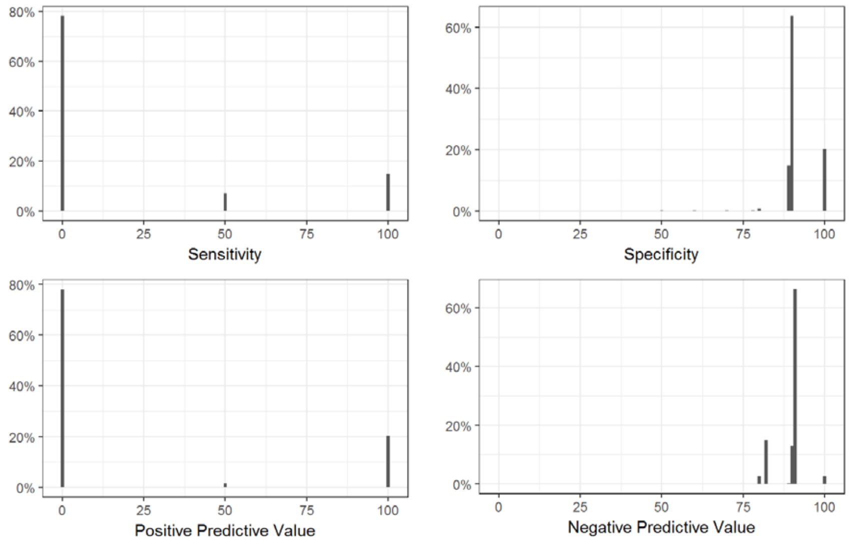

42 FUNGAL INFECTION

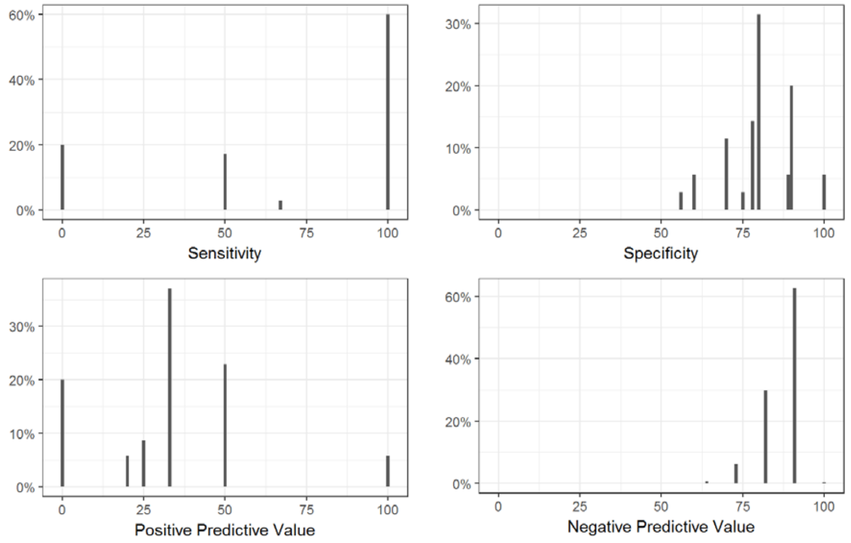

43 GASTROINTESTINAL HAEMORRHAGE

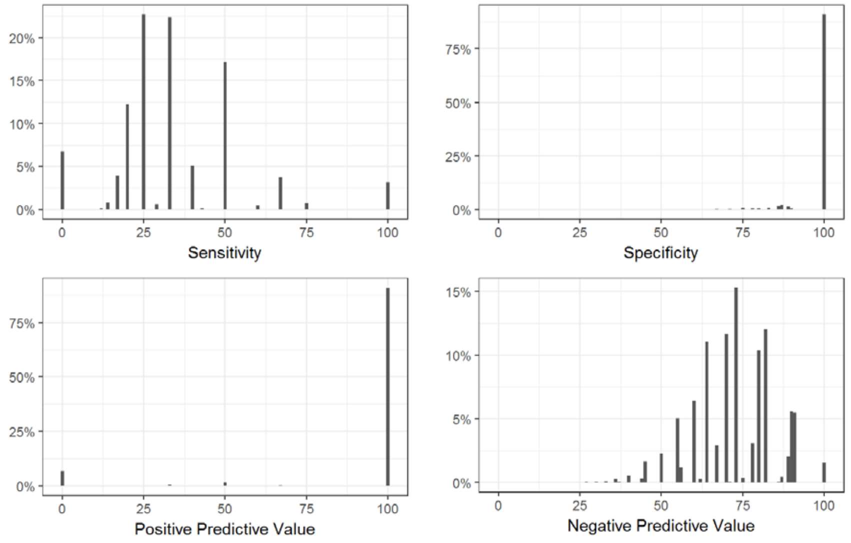

44 GLAUCOMA

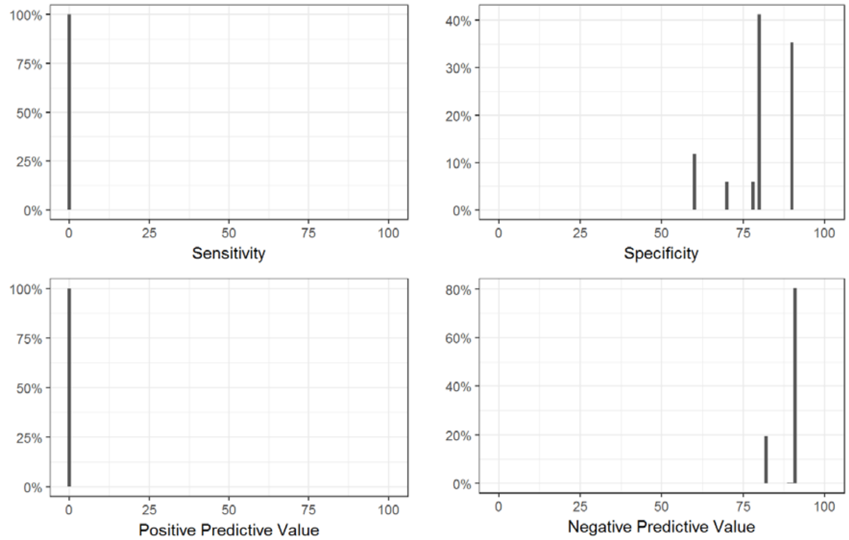

45 HAEMATOMA

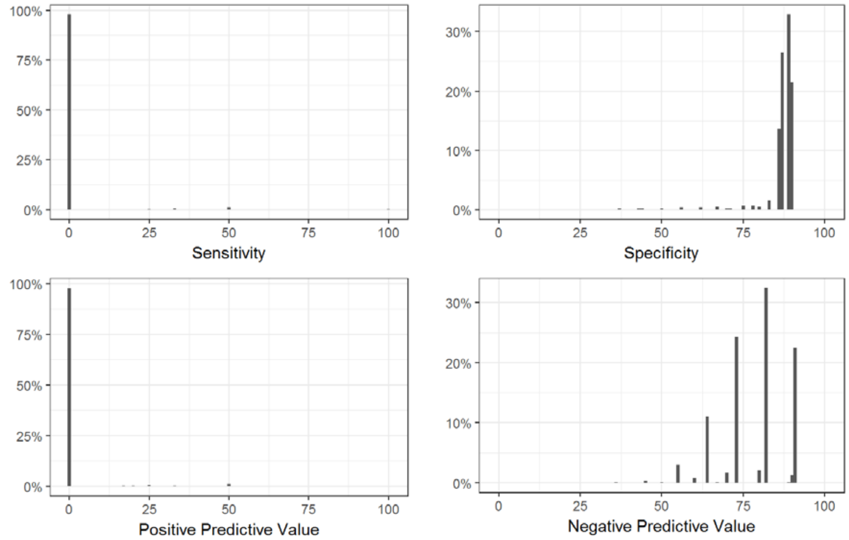

46 HAEMOLYTIC ANAEMIA

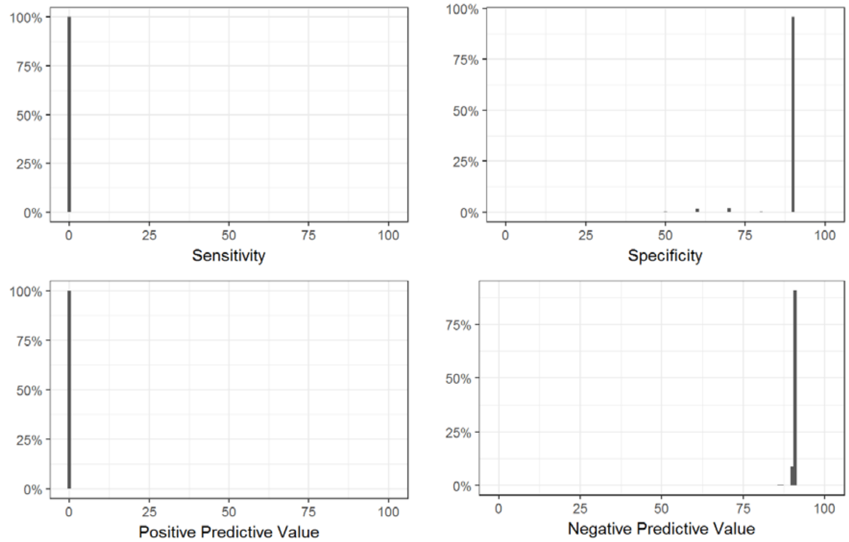

47 HAEMORRHAGE

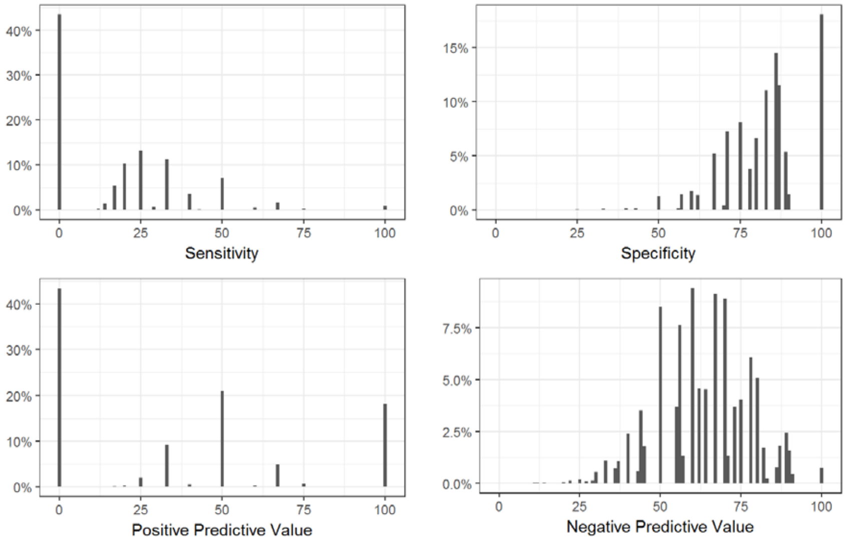

48 HALLUCINATION

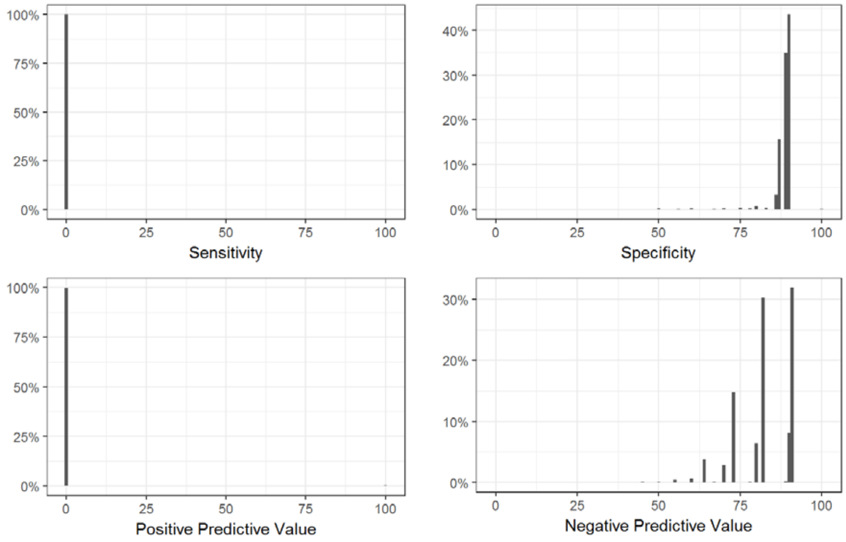

49 HEPATIC FAILURE

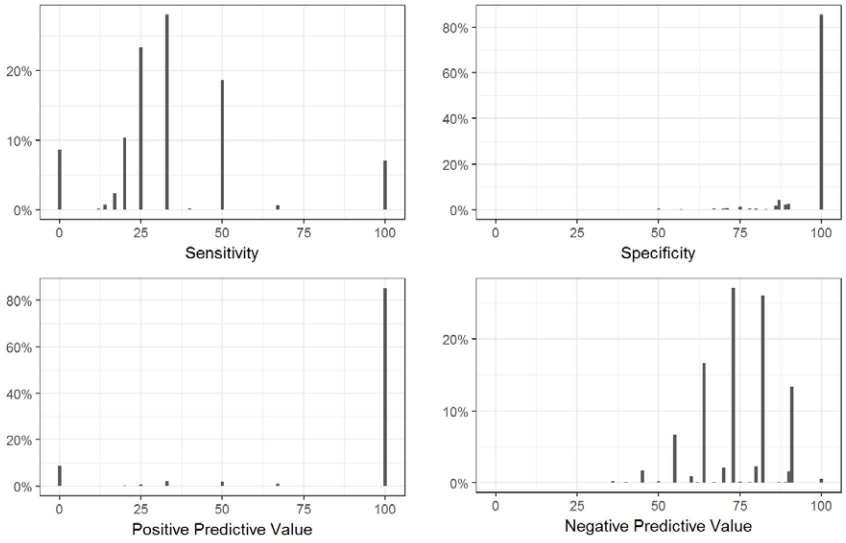

50 HEPATITIS

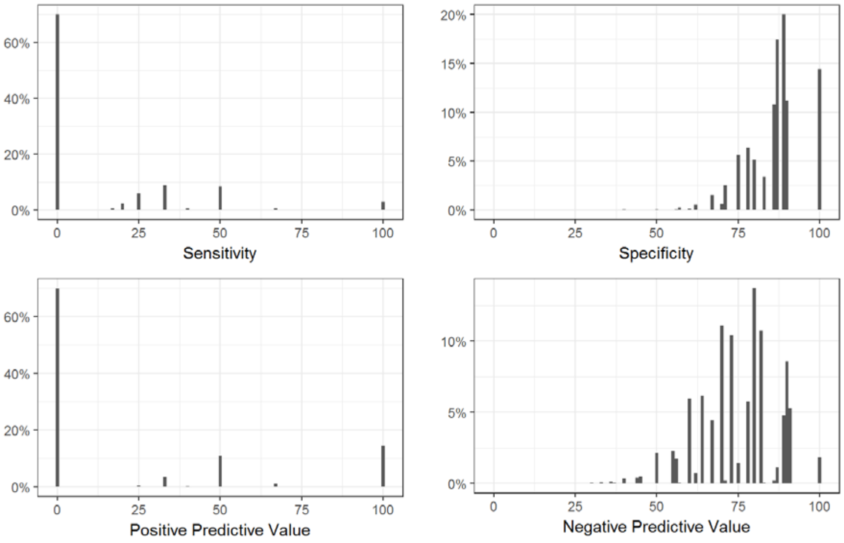

51 HEPATOTOXICITY

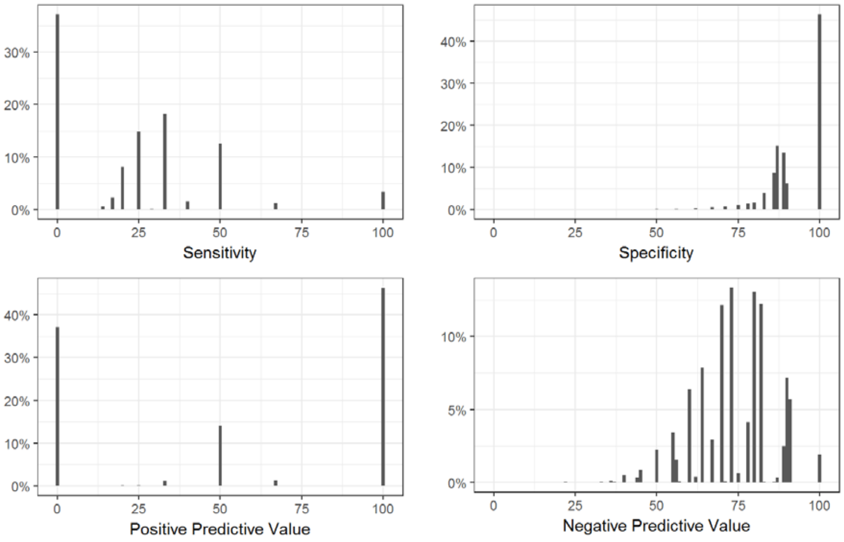

52 HOSTILITY

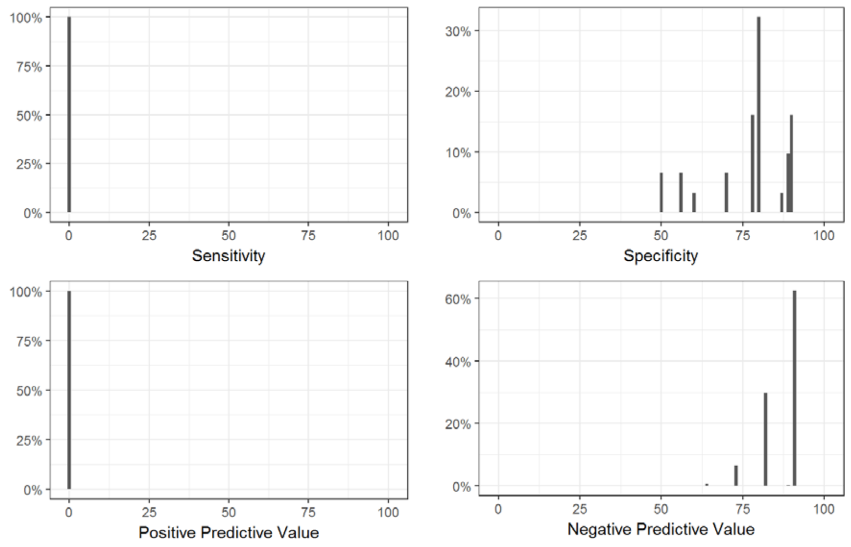

53 HYPERCHOLESTEROLAEMIA

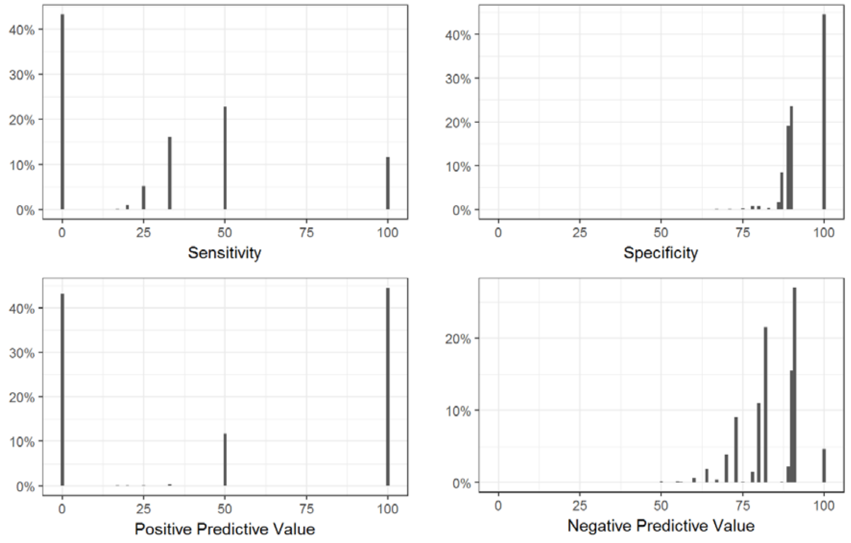

54 HYPERGLYCAEMIA

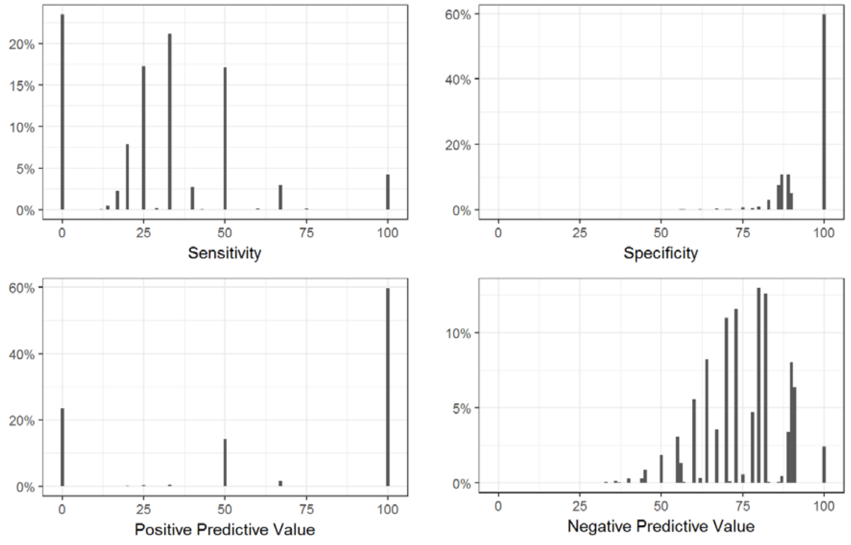

55 HYPERKINESIA

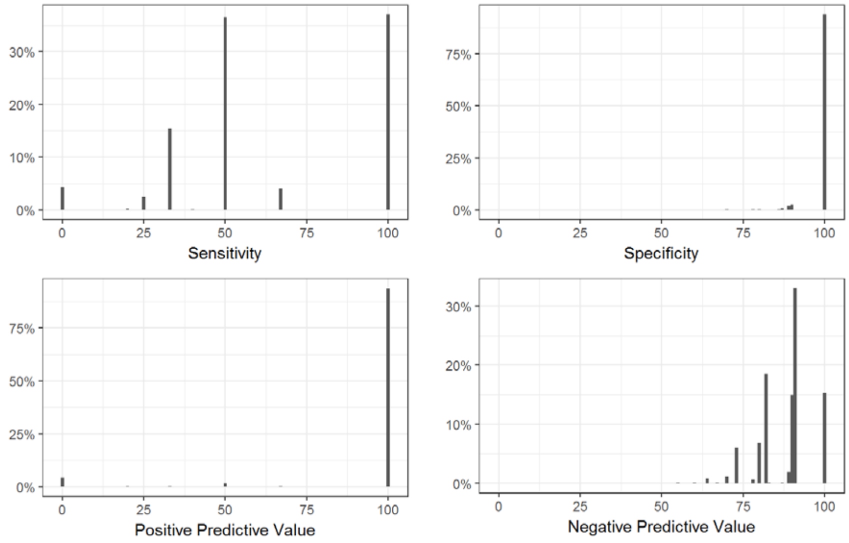

56 HYPERSENSITIVITY

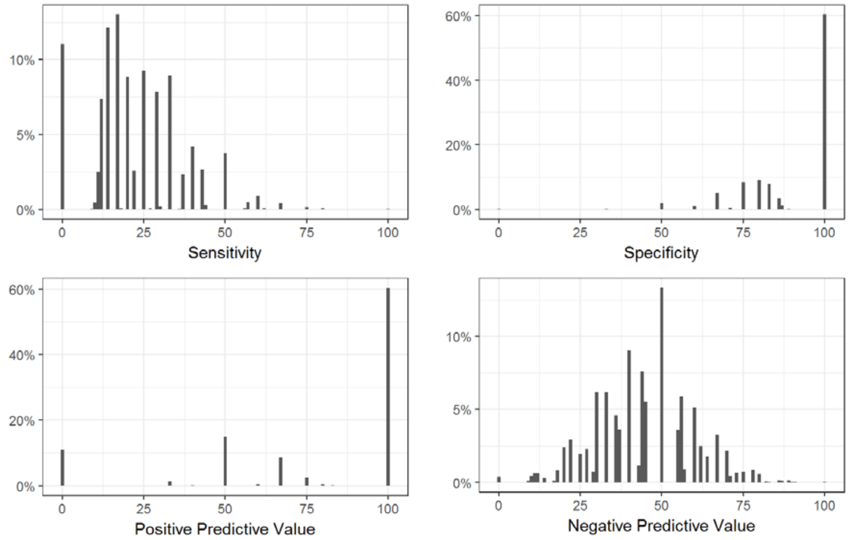

57 HYPERTENSION

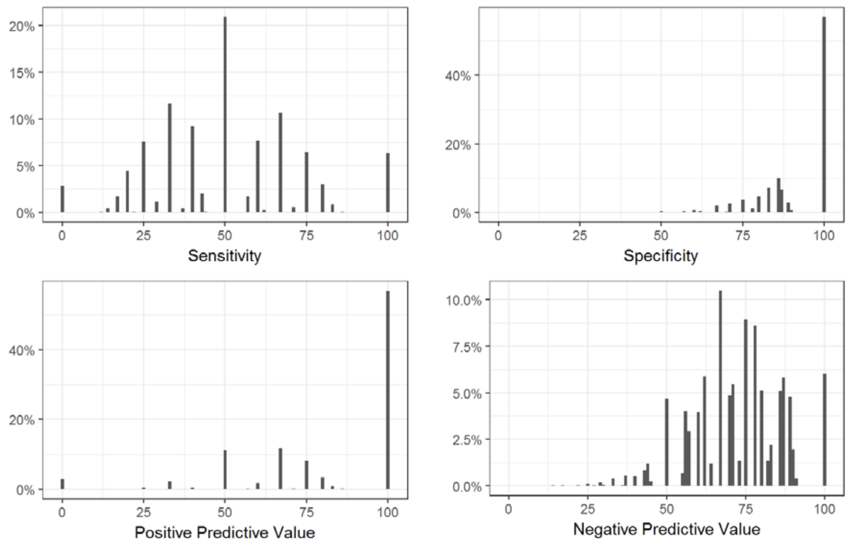

58 HYPOGLYCAEMIA

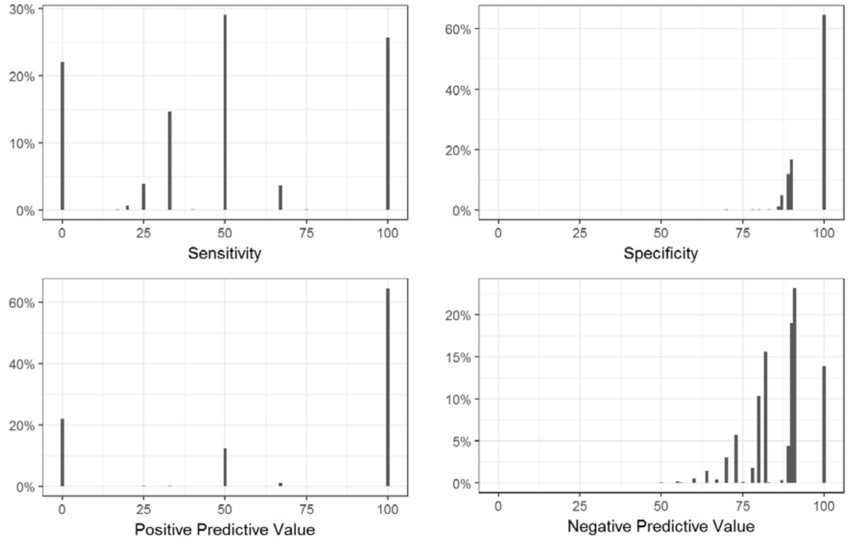

59 IMPAIRED HEALING

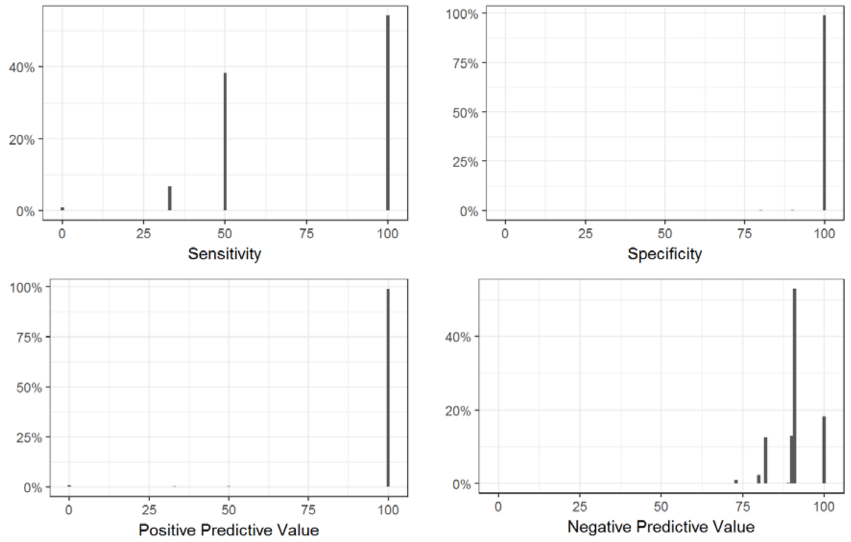

60 INFECTION

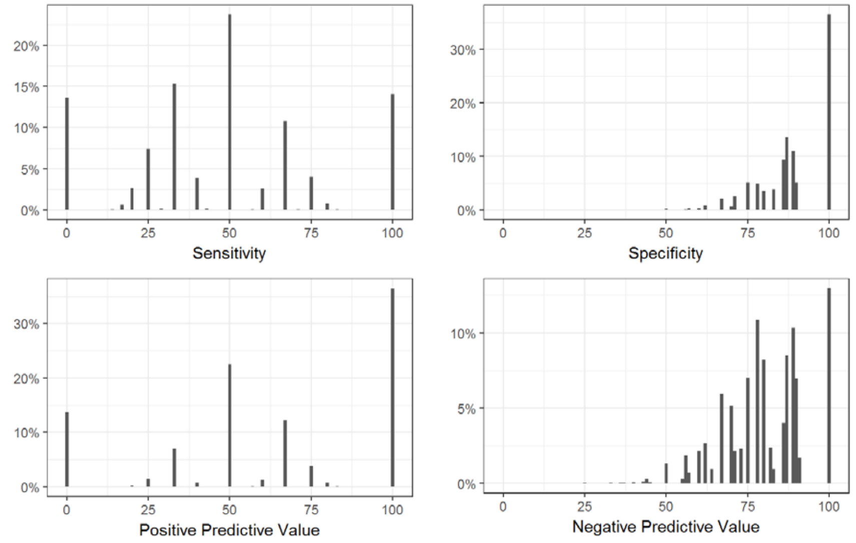

61 INSOMNIA

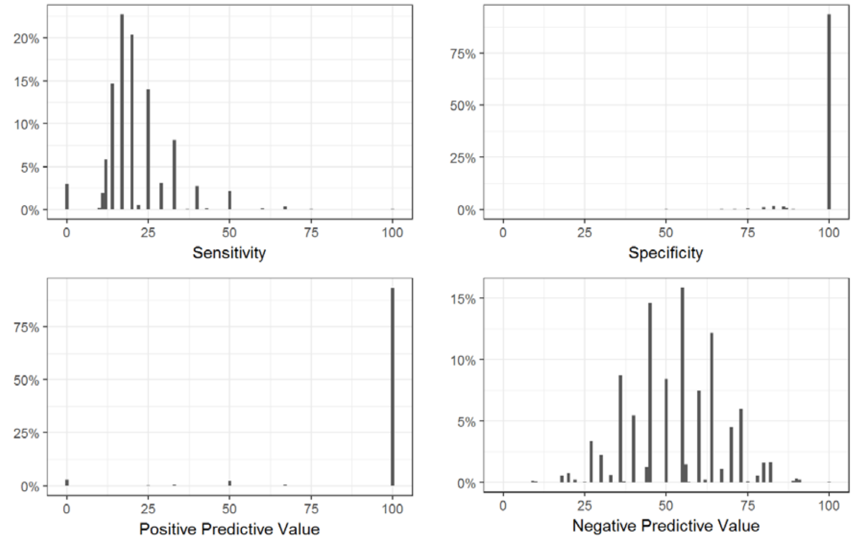

62 INTERSTITIAL LUNG DISEASE

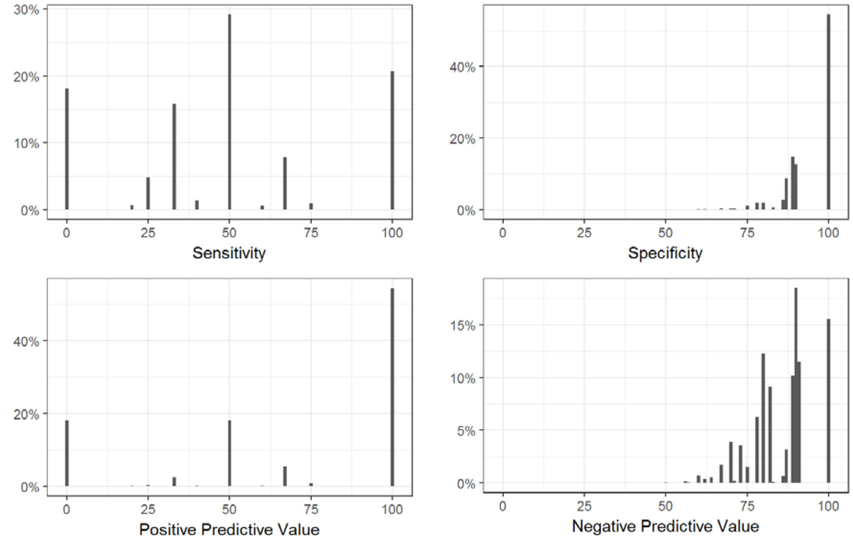

63 JAUNDICE

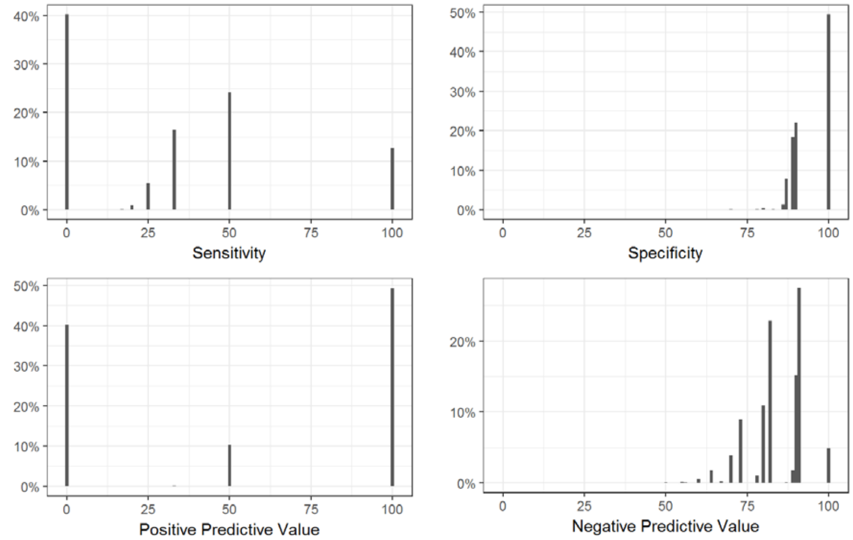

64 LARYNGEAL OEDEMA

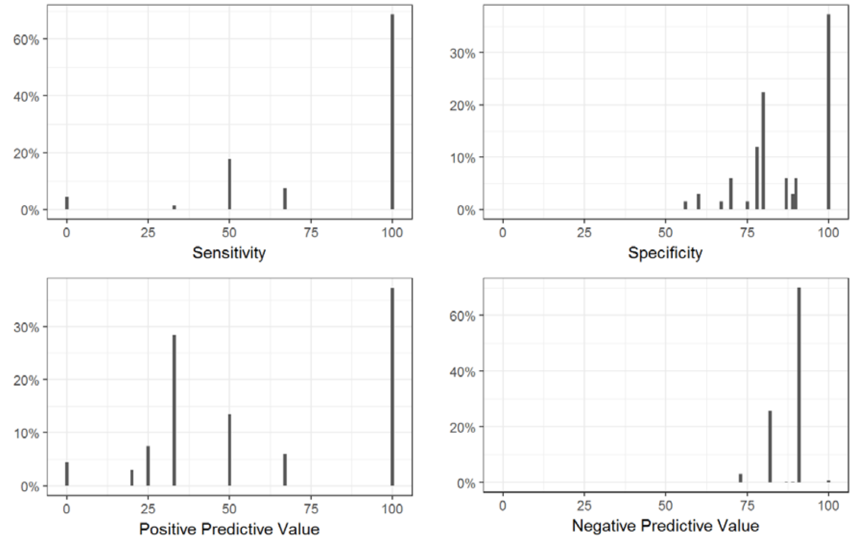

65 LEUKOPENIA

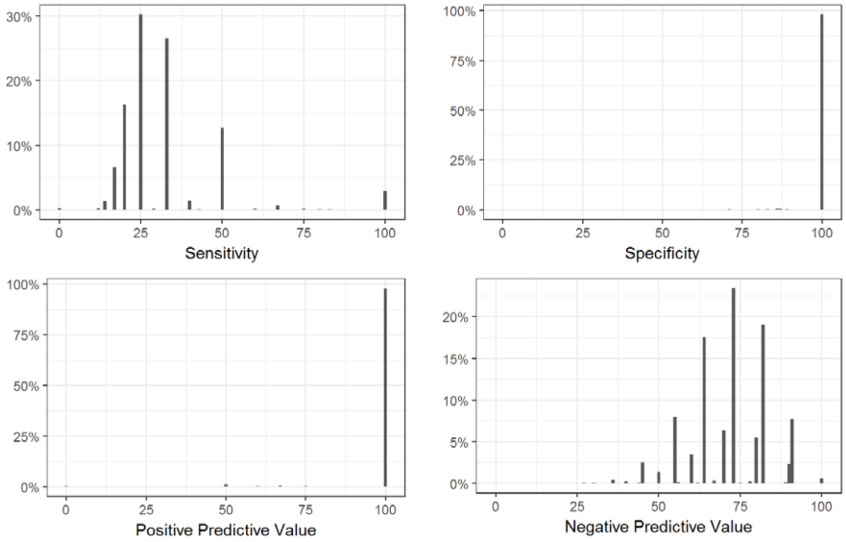

66 MEMORY IMPAIRMENT

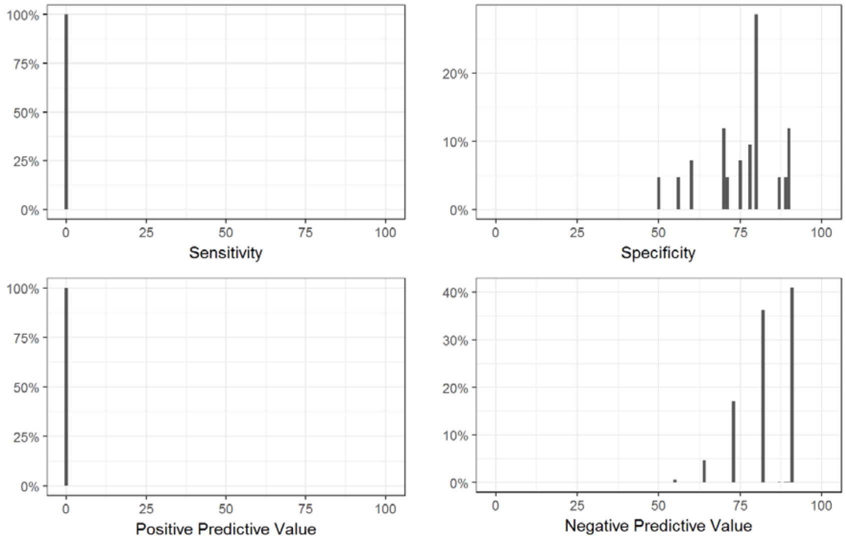

67 MYOCARDIAL INFARCTION

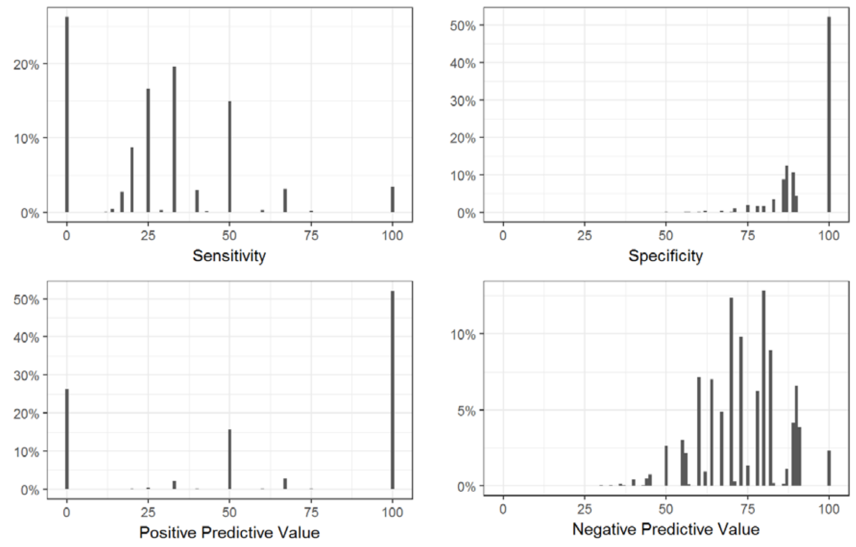

68 MYOSITIS

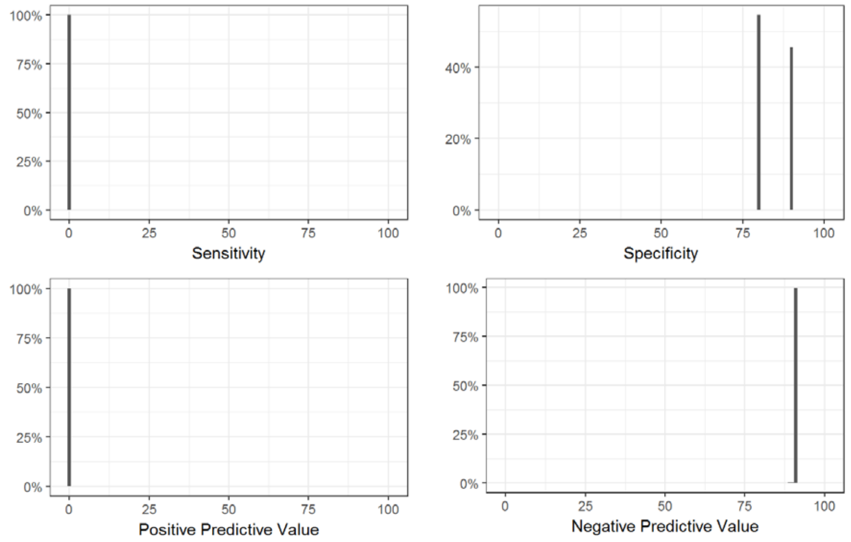

69 NEUROLEPTIC MALIGNANT SYNDROME

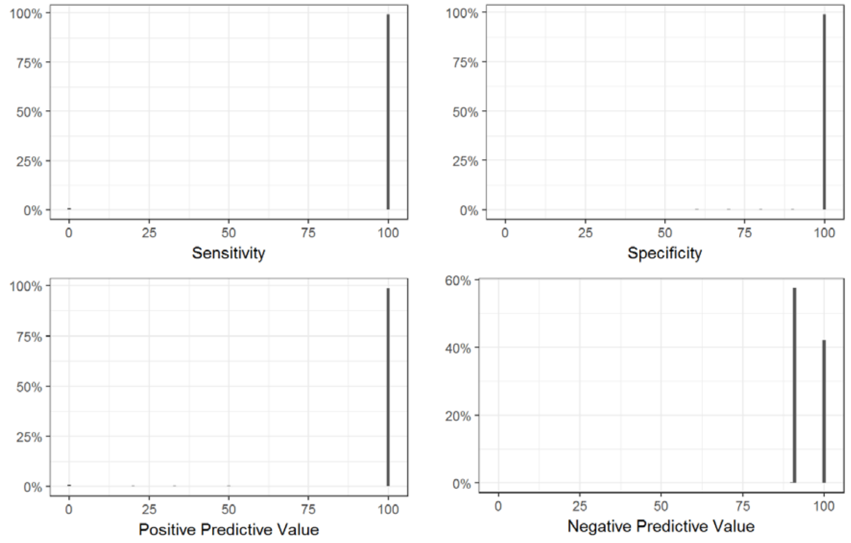

70 NEUROPATHY PERIPHERAL

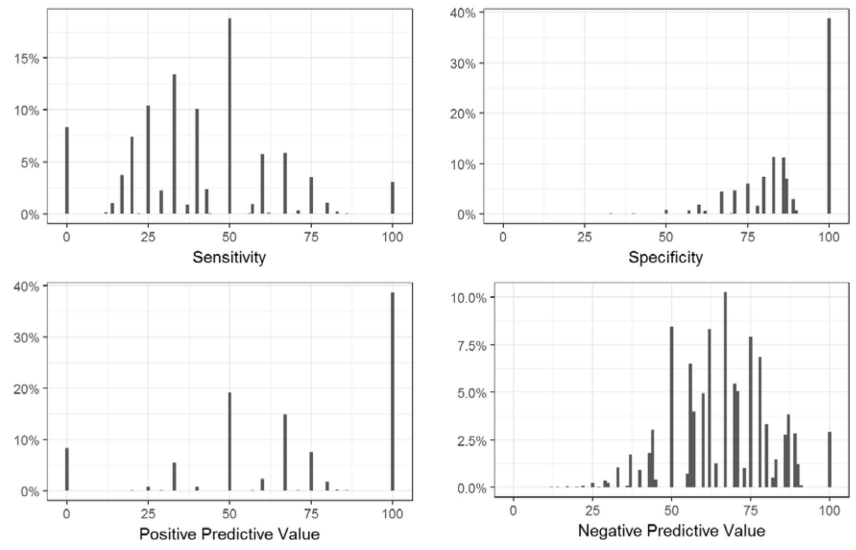

71 NEUTROPENIA

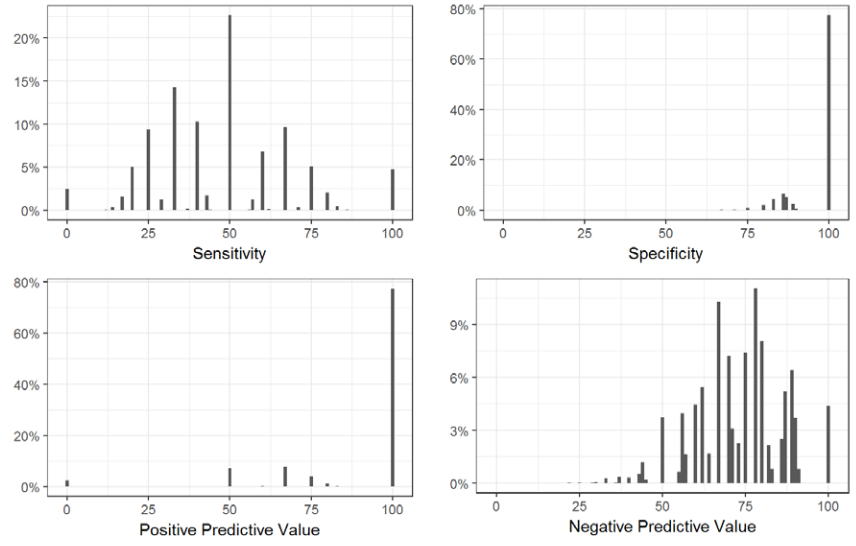

72 OEDEMA

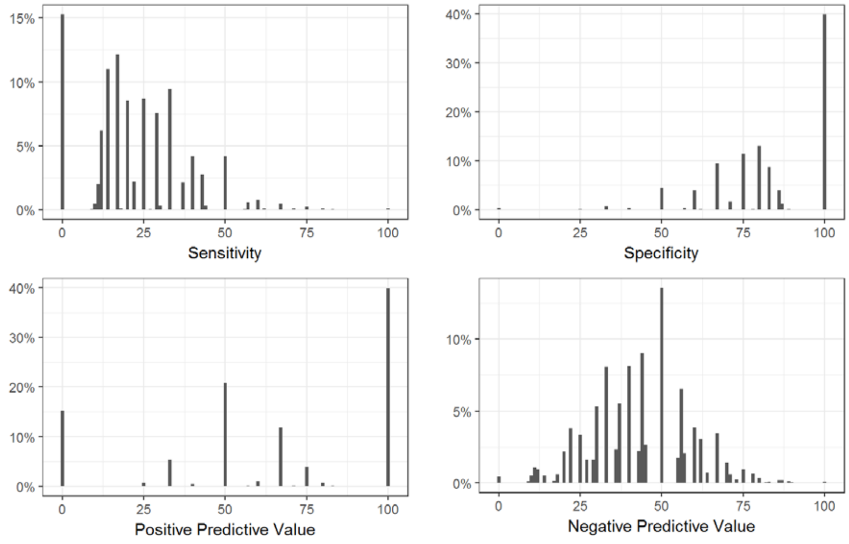

73 PANCREATITIS

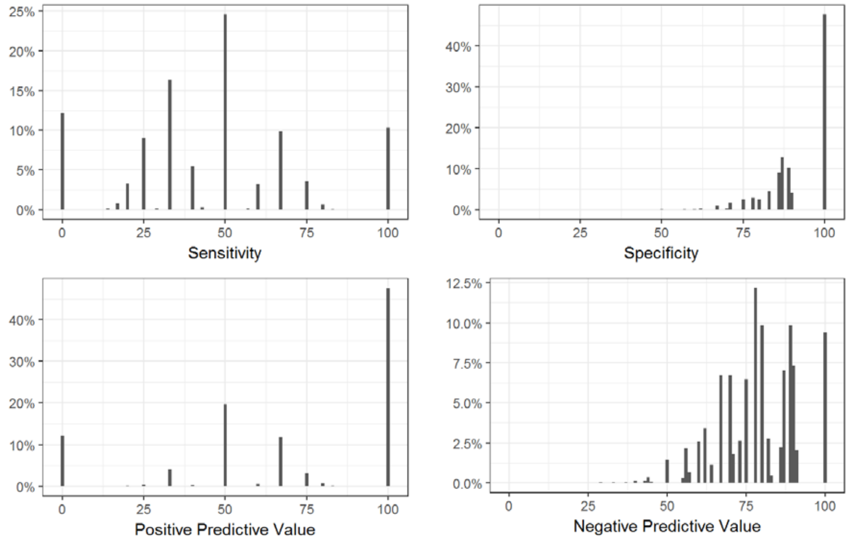

74 PANCYTOPENIA

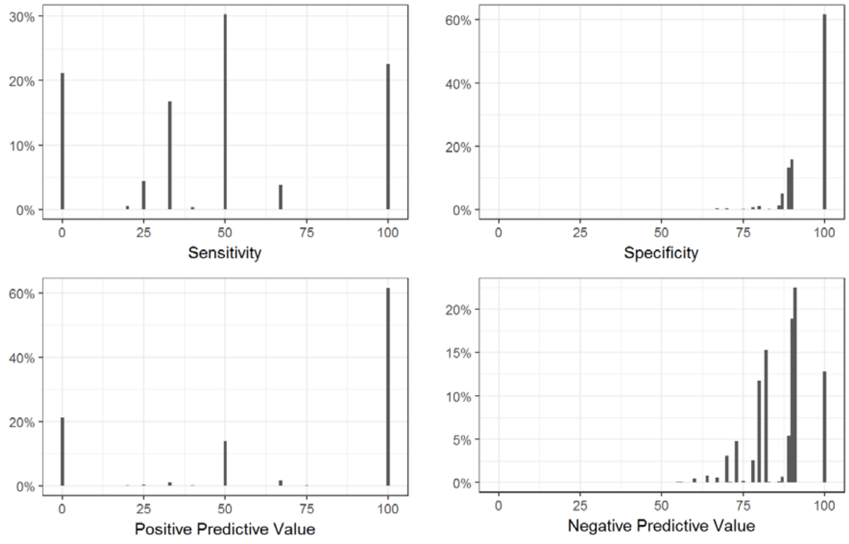

75 PARALYSIS

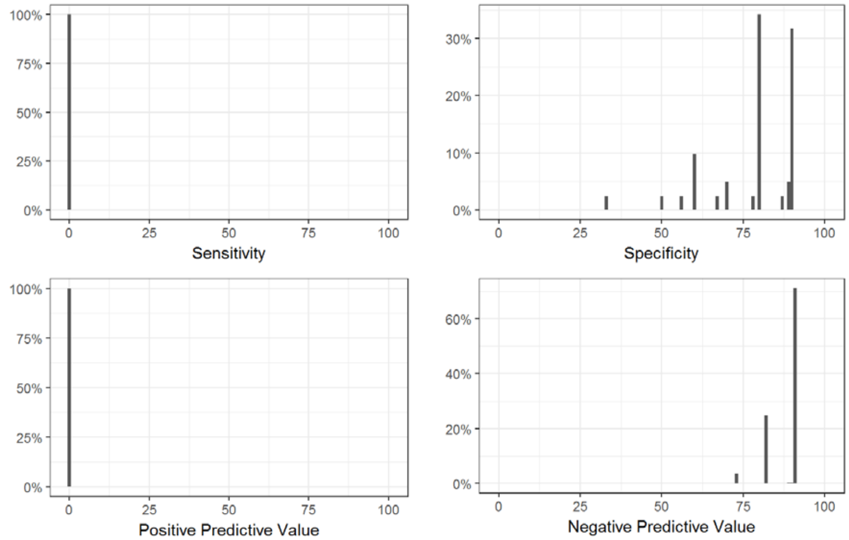

76 PARANOIA

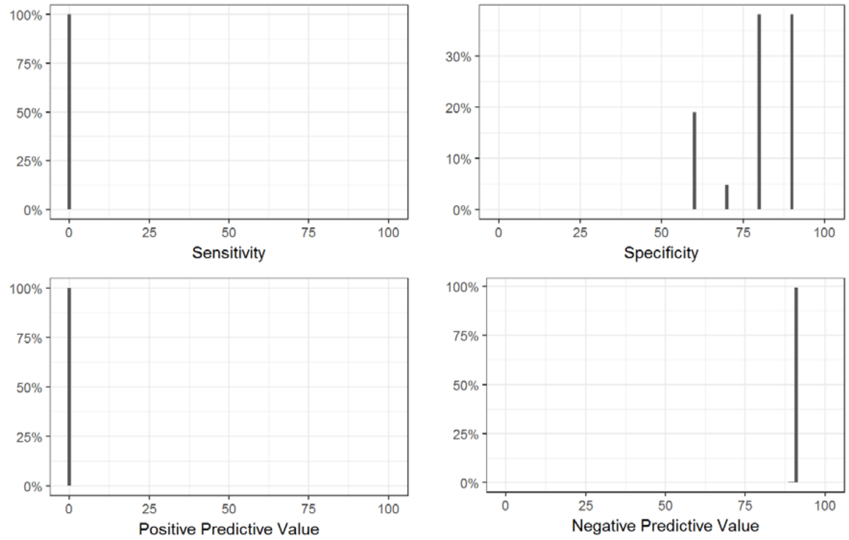

77 PHOTOSENSITIVITY REACTION

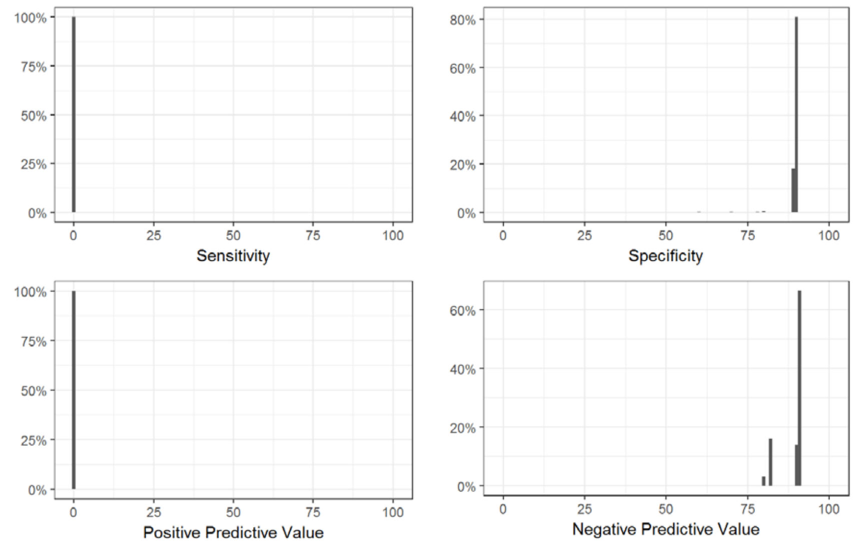

78 PNEUMONIA

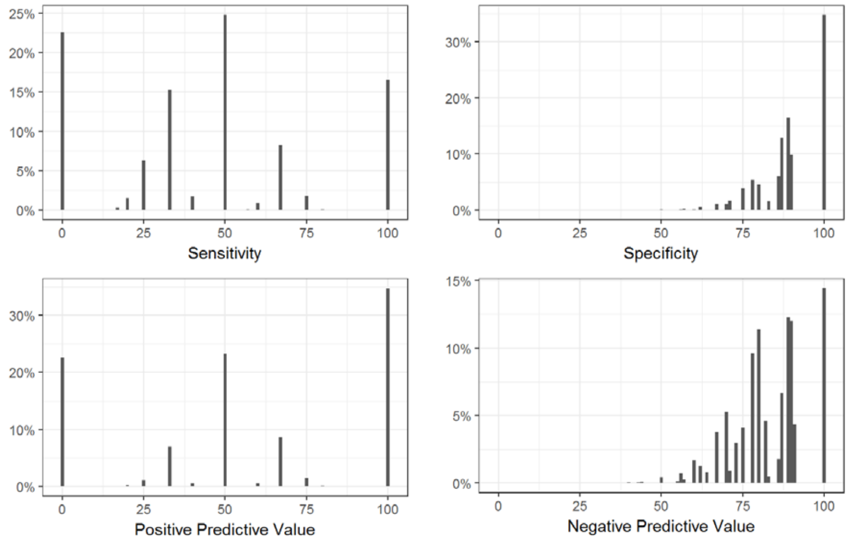

79 PROTEINURIA

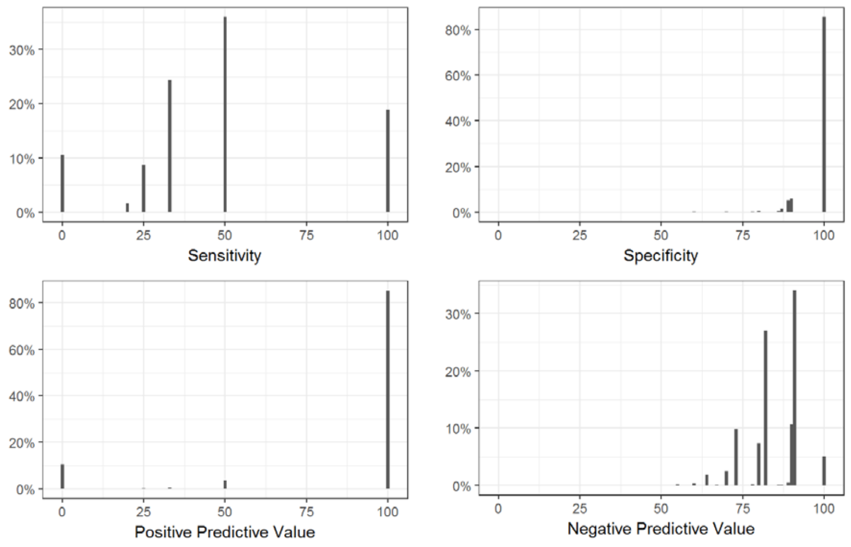

80 PULMONARY EMBOLISM

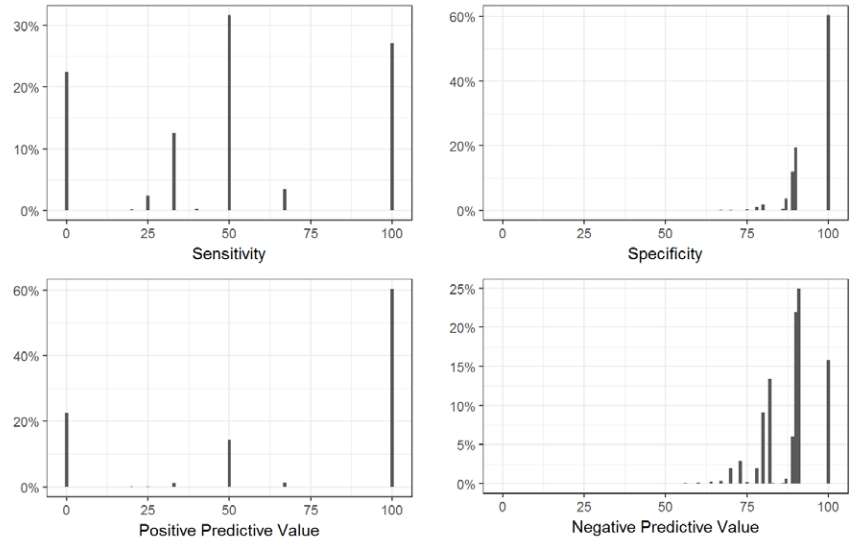

81 PULMONARY OEDEMA

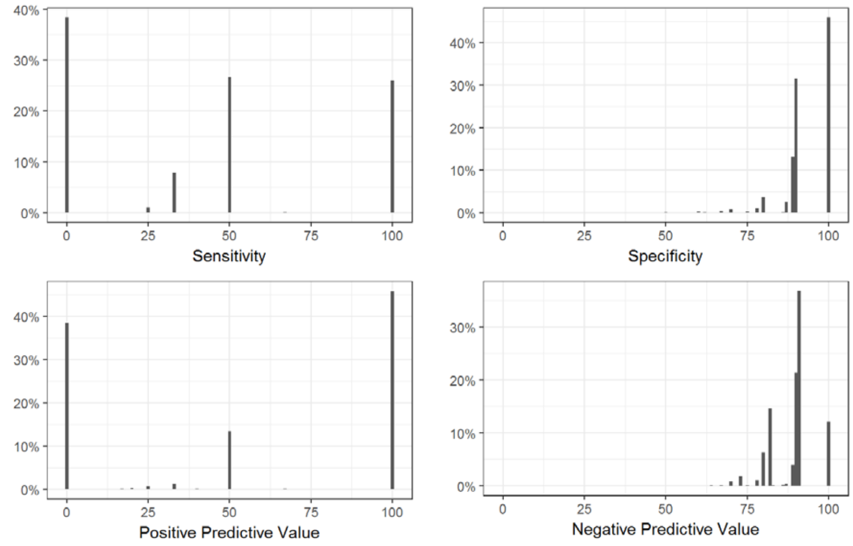

82 RECTAL HAEMORRHAGE

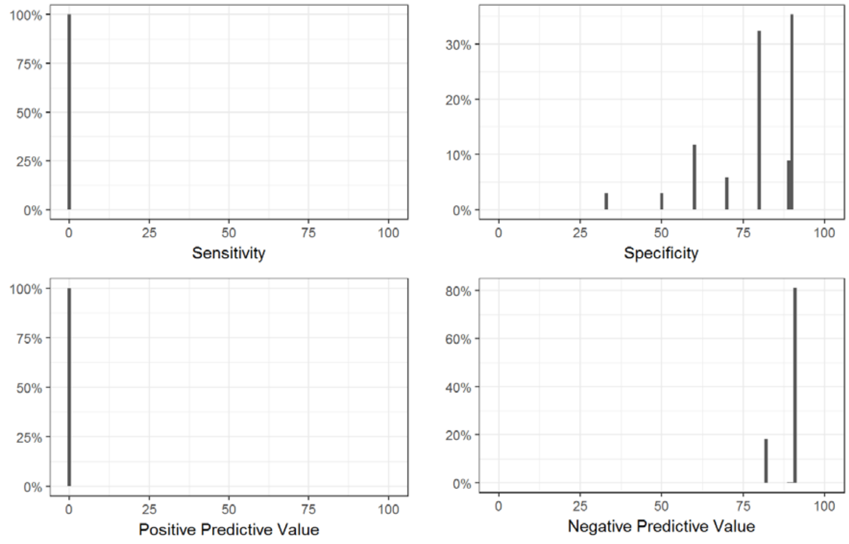

83 RENAL FAILURE

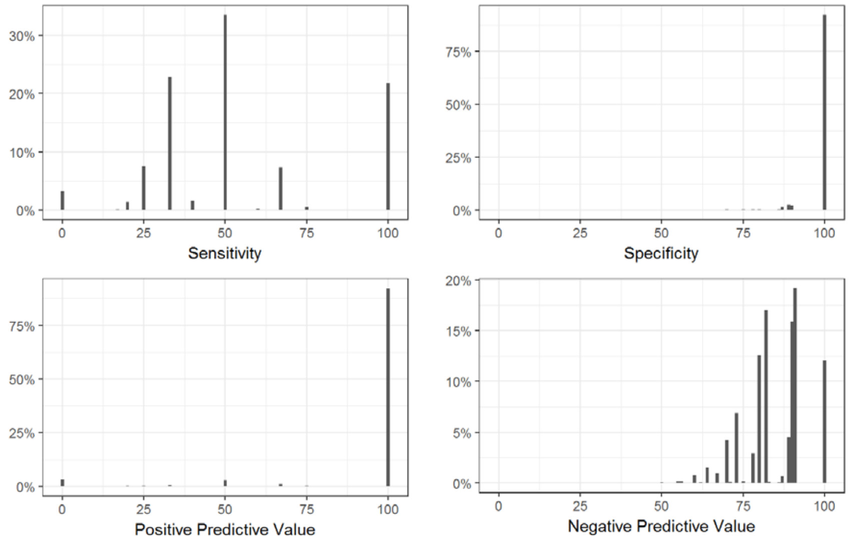

84 RENAL IMPAIRMENT

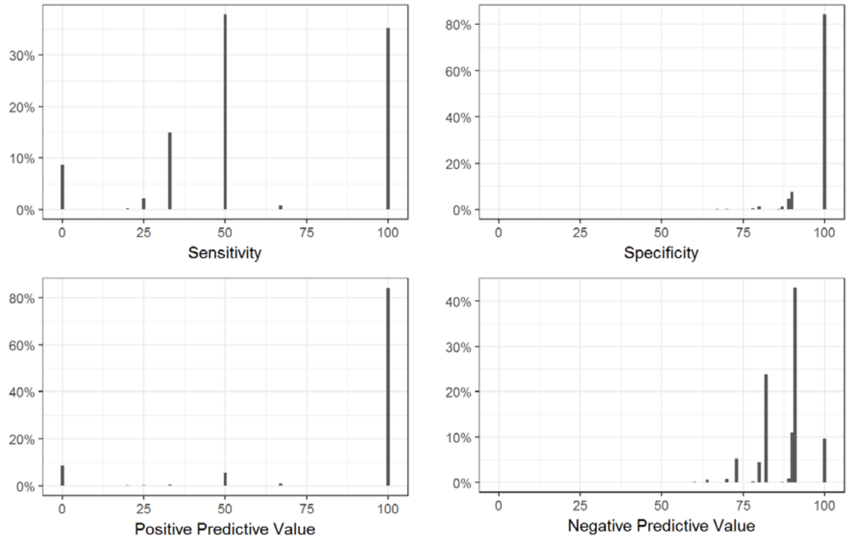

85 RESPIRATORY FAILURE

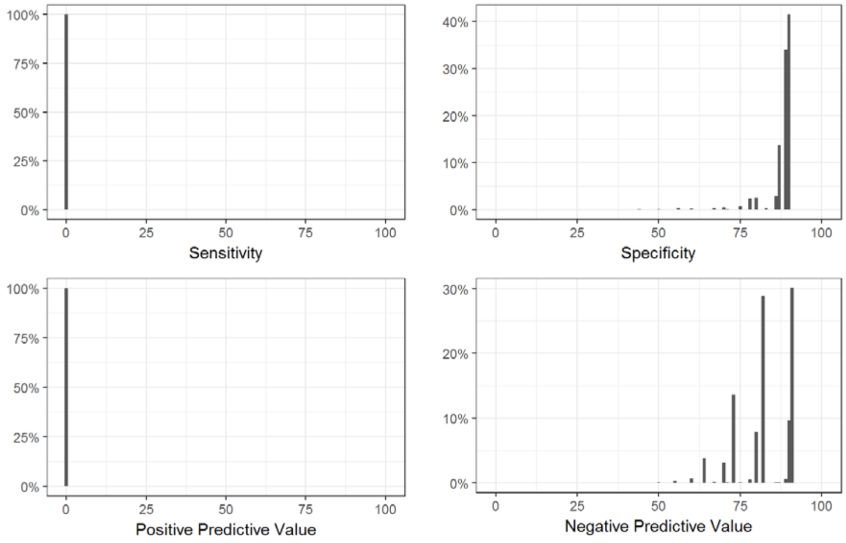

86 RHABDOMYOLYSIS

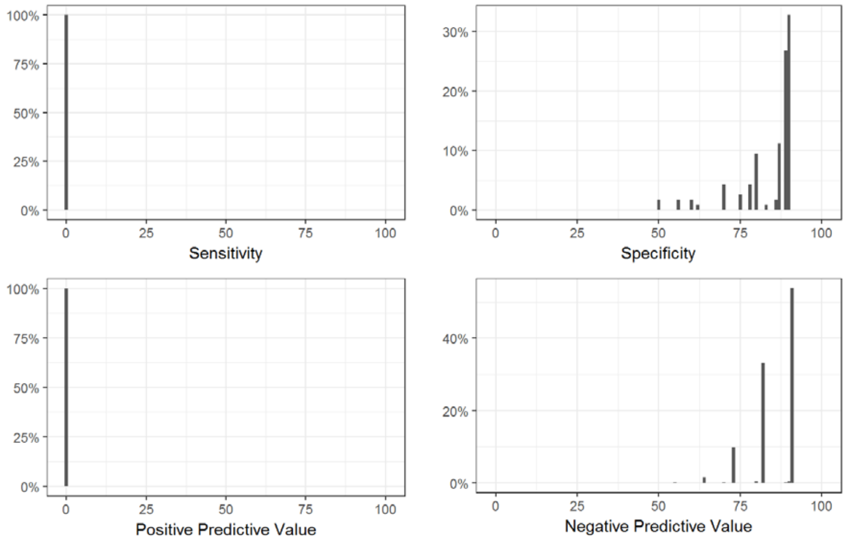

87 SEIZURE

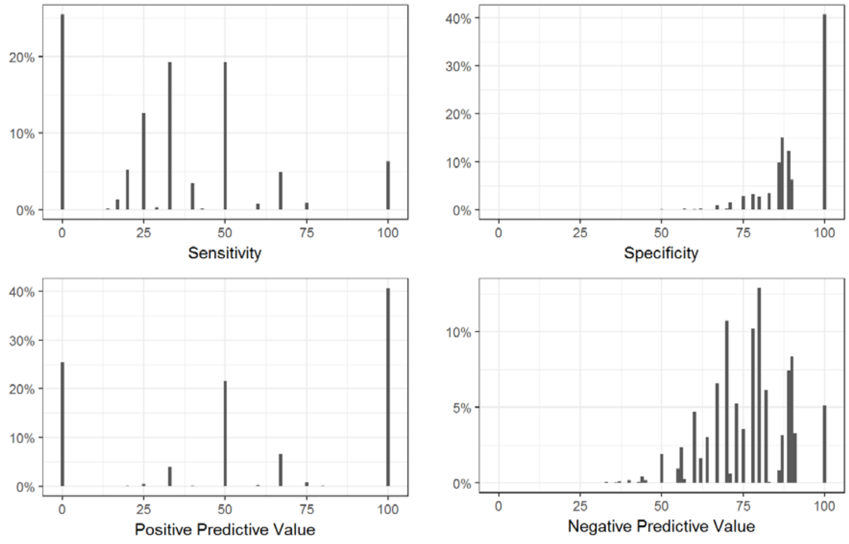

88 SEPSIS

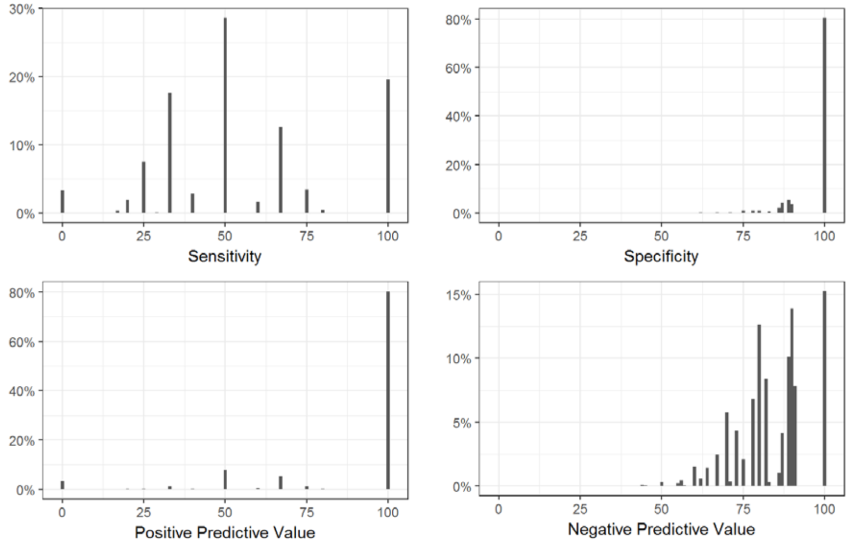

89 SEROTONIN SYNDROME

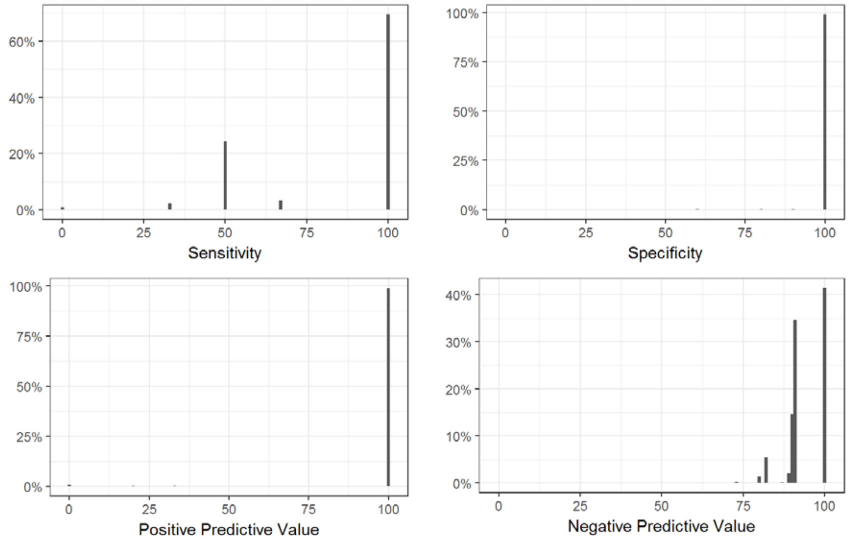

90 SLEEP DISORDER

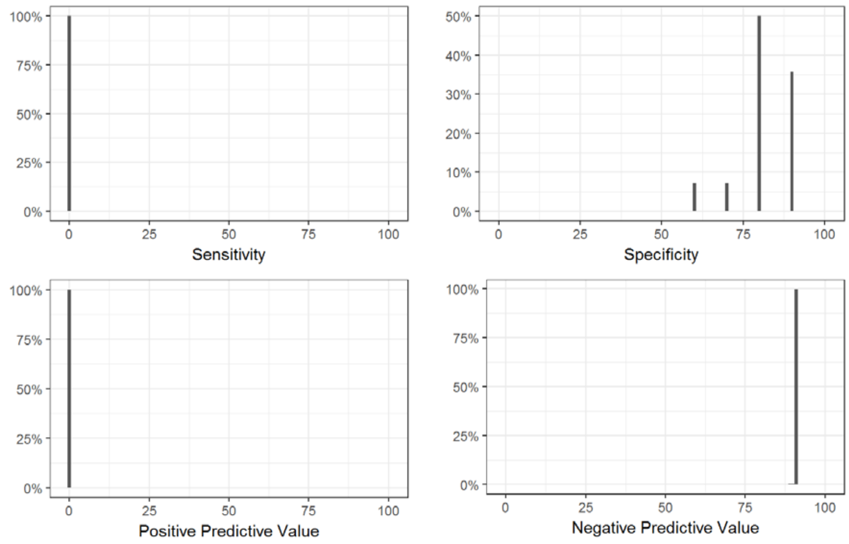

91 STEVENS-JOHNSON SYNDROME

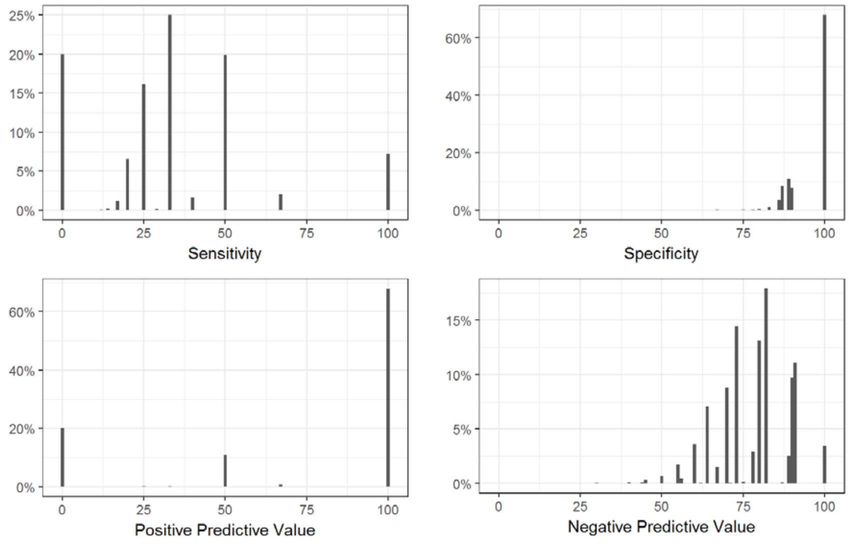

92 STOMATITIS

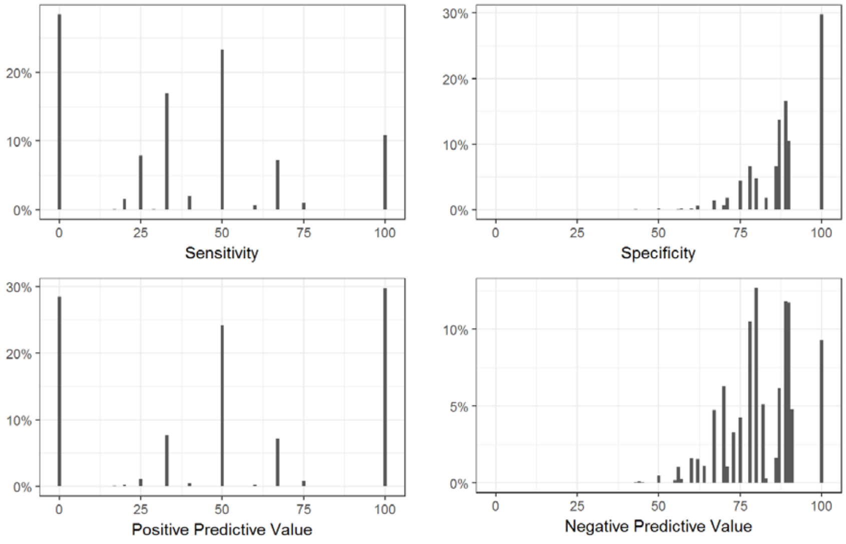

93 SUDDEN DEATH

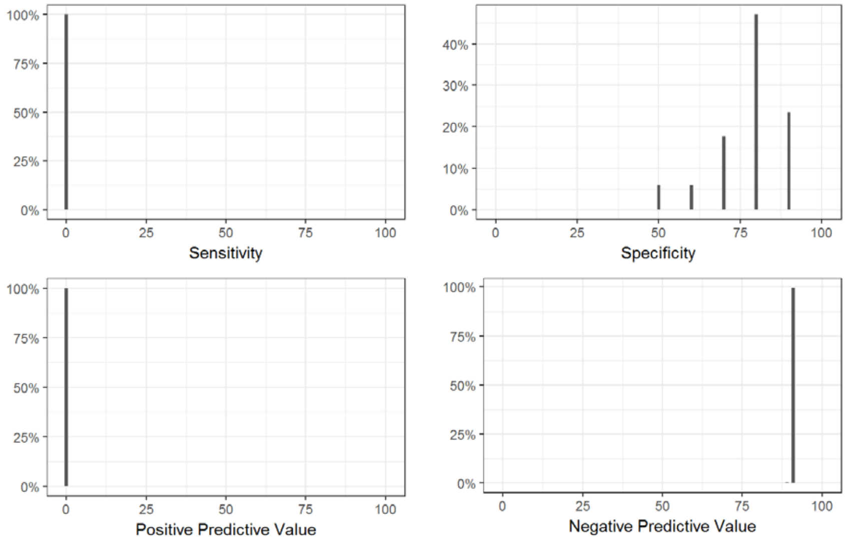

94 SUICIDAL BEHAVIOUR

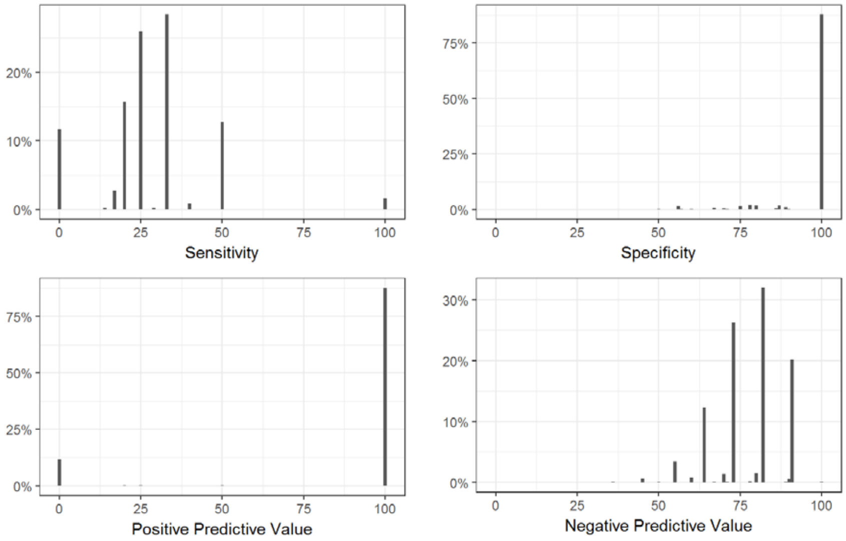

95 SUPRAVENTRICULAR TACHYCARDIA

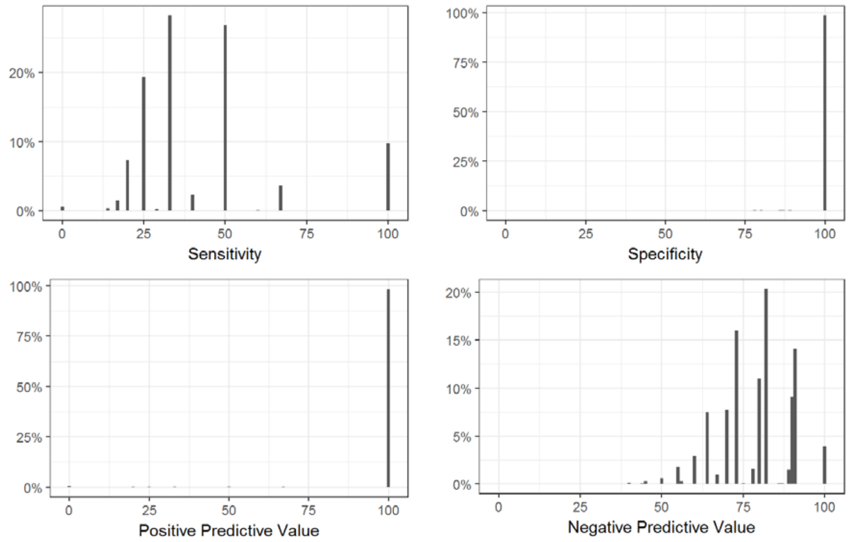

96 TACHYCARDIA

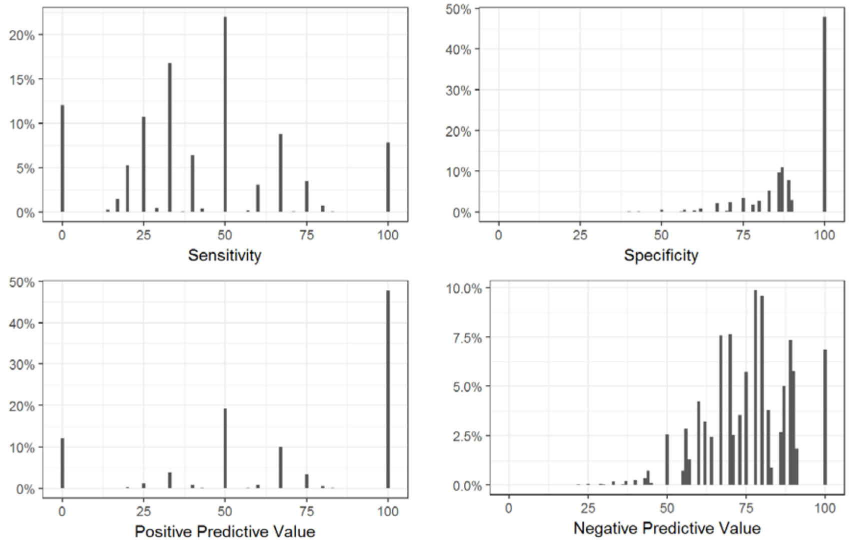

97 THROMBOCYTOPENIA

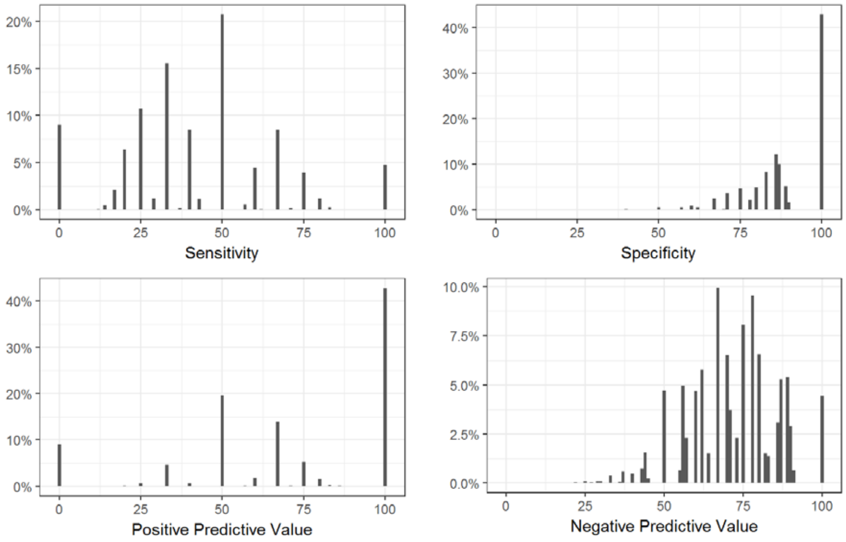

98 THROMBOPHLEBITIS

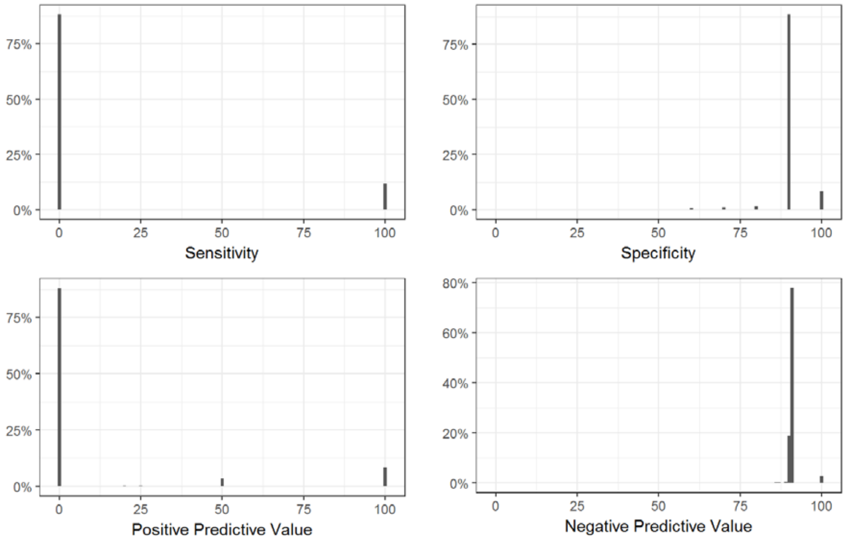

99 TINNITUS

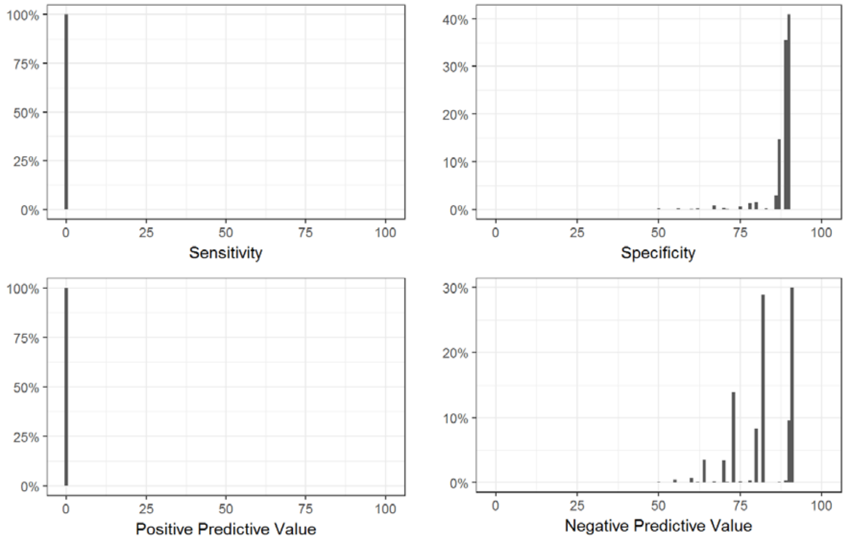

100 TOXIC EPIDERMAL NECROLYSIS

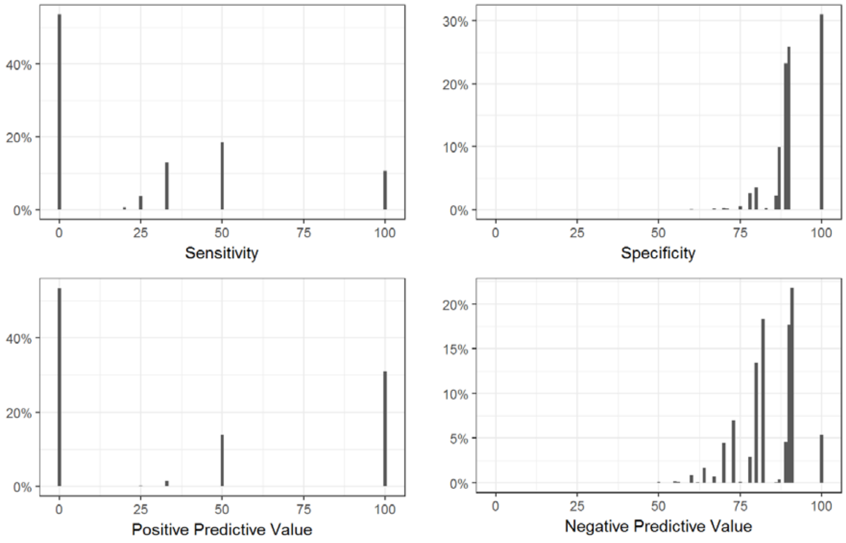

101 UPPER RESPIRATORY TRACT INFECTION

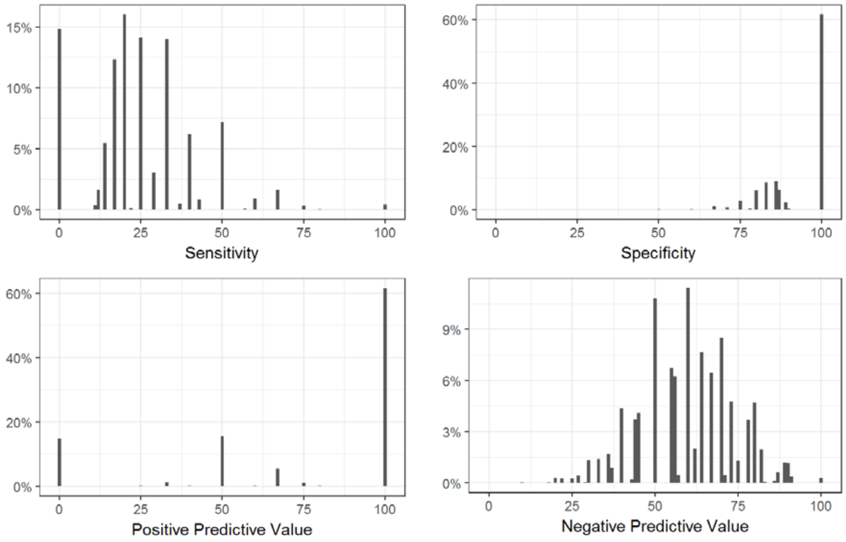

102 URINARY TRACT INFECTION

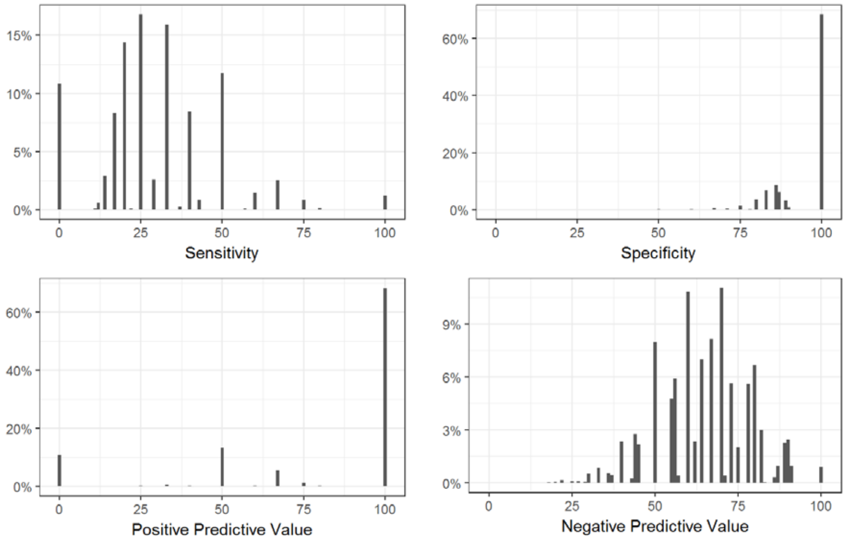

103 URTICARIA

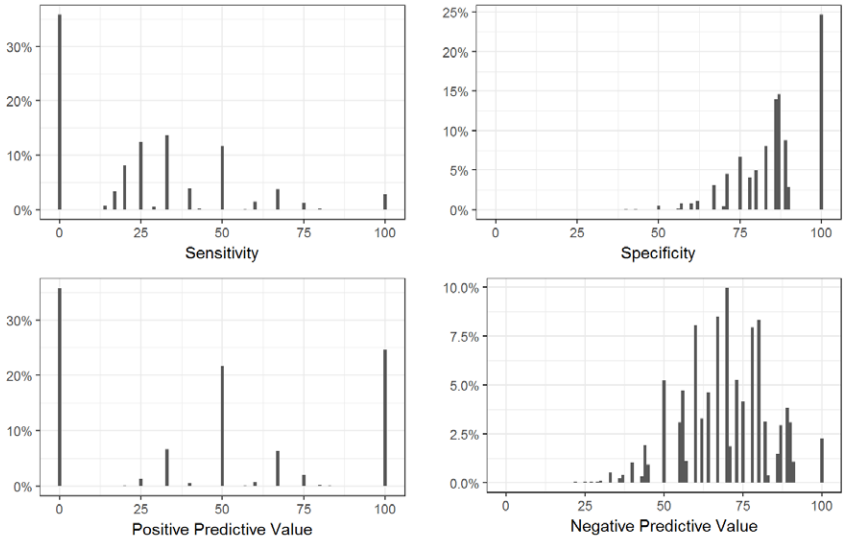

104 VAGINAL HAEMORRHAGE

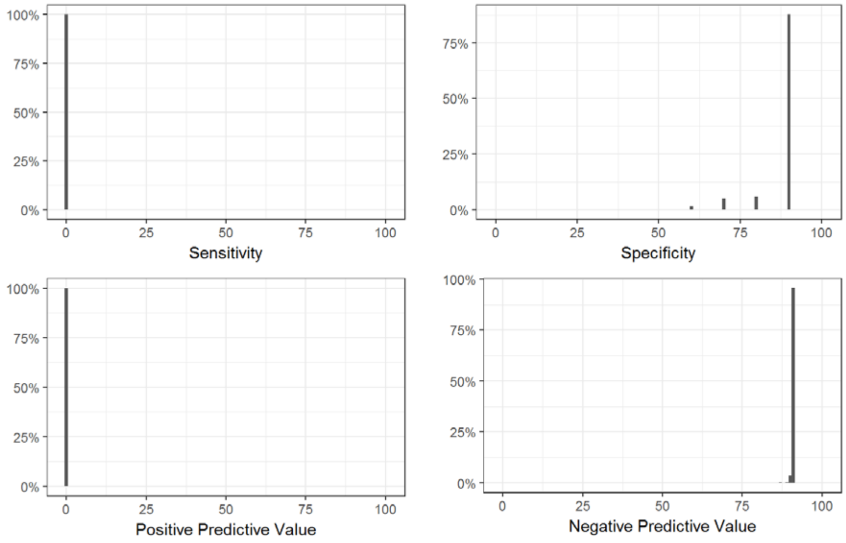

105 VASCULITIS

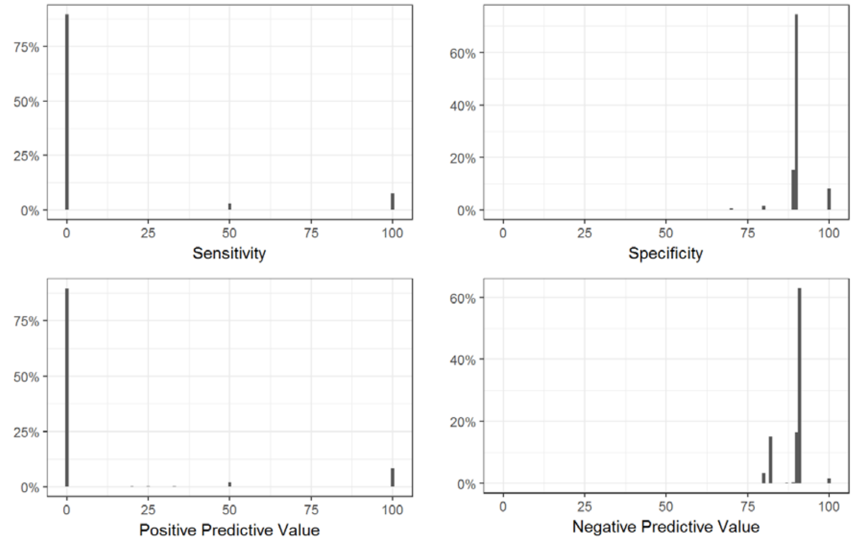

106 VENTRICULAR ARRHYTHMIA

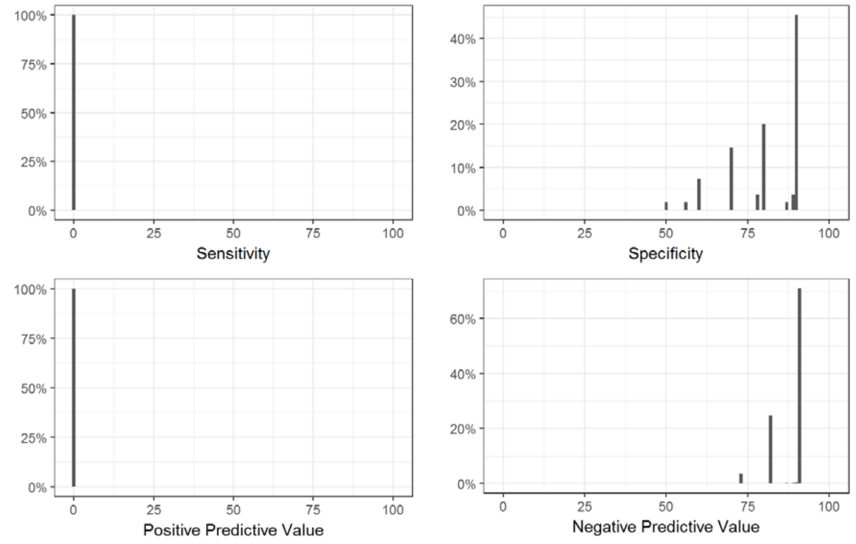

107 VISION BLURRED

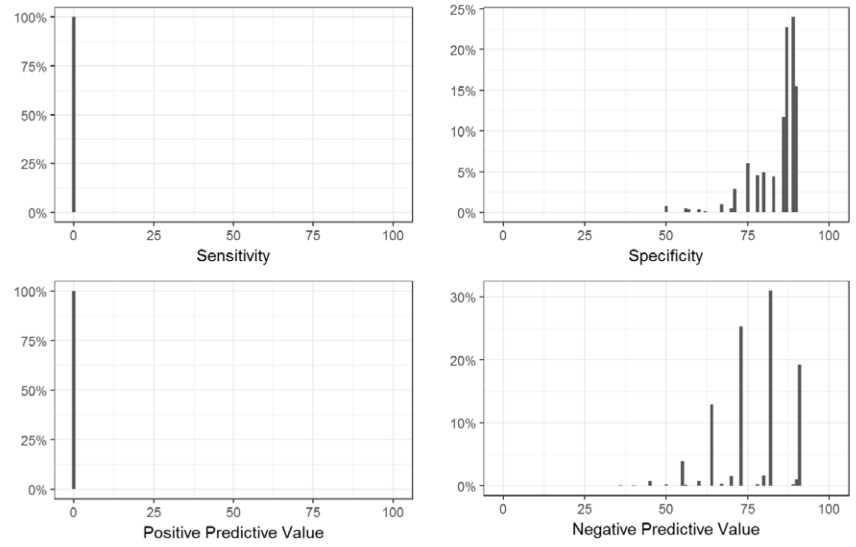

108 VISUAL IMPAIRMENT

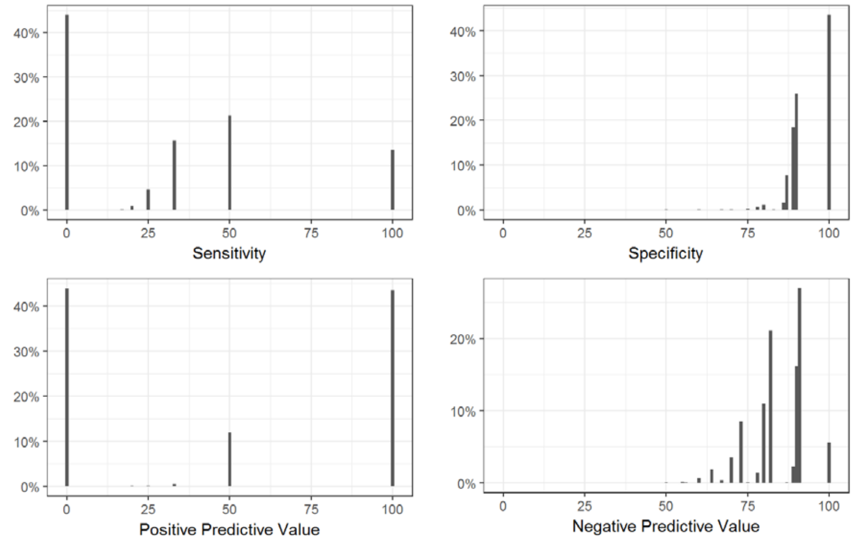

109 WEIGHT INCREASED

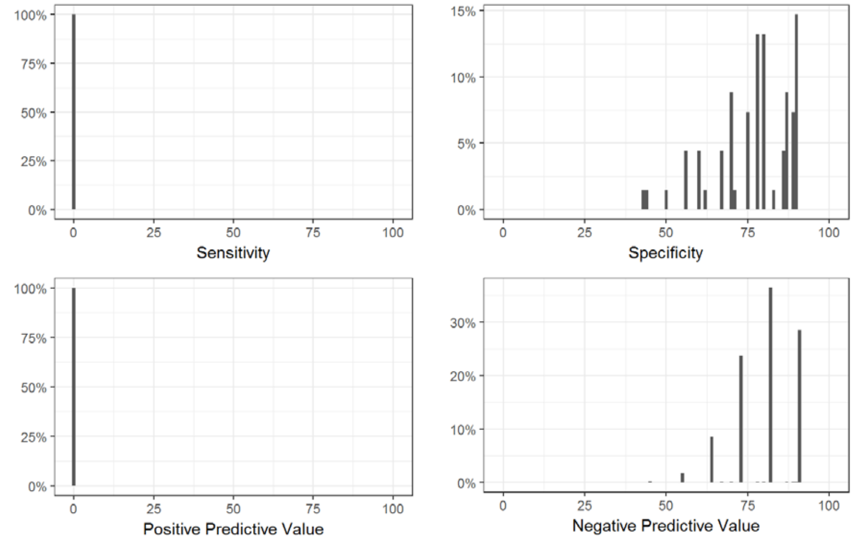

110 Overall

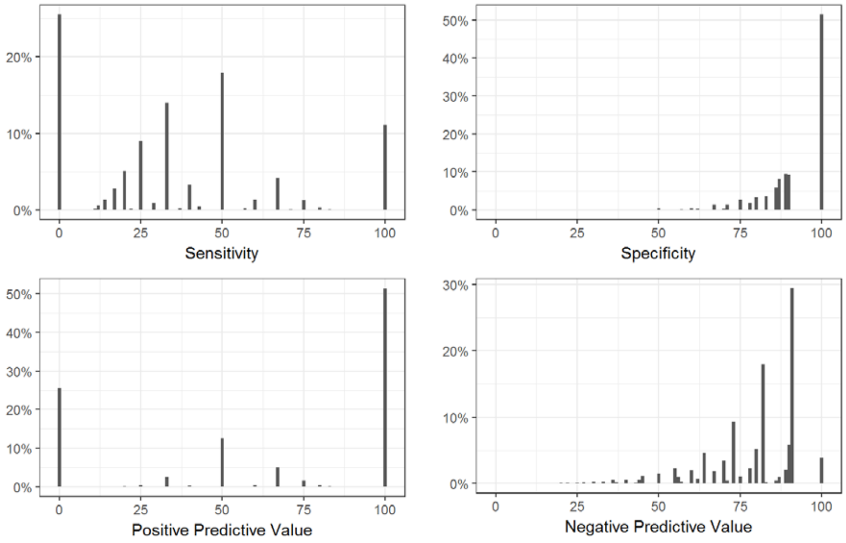

20%
10%
0%
0
25
50
75
100
Sensitivity
50%
40%
30%
20%
10%
0%
Specificity
Positive Predictive Value
30%
Negative Predictive Value

Supplemental Table 1. The targets represented by 54 test drugs used in the study

**Targets represented by 54 test drugs used in the study**			
3-hydroxy-3-methylglutaryl-coenzyme a reductase	4-aminobutyrate aminotransferase, mitochondrial	5-hydroxytryptamine receptor 1a	5-hydroxytryptamine receptor 1b
5-hydroxytryptamine receptor 2a	5-hydroxytryptamine receptor 3a	5-hydroxytryptamine receptor 6	5-hydroxytryptamine receptor 7
alpha-1a adrenergic receptor	alpha-1b adrenergic receptor	alpha-1d adrenergic receptor	alpha-2a adrenergic receptor
alpha-2b adrenergic receptor	alpha-2c adrenergic receptor	androgen receptor	angiopoietin-1 receptor
beta-1 adrenergic receptor	beta-3 adrenergic receptor	b-lymphocyte antigen cd20	breakpoint cluster region protein
cgmp-specific 3',5'-cyclic phosphodiesterase	coagulation factor x	d(1a) dopamine receptor	d(2) dopamine receptor
d(3) dopamine receptor	d(4) dopamine receptor	delta-type opioid receptor	dihydrofolate reductase
dihydroorotate dehydrogenase (quinone), mitochondrial	dipeptidyl peptidase 4	discoidin domain-containing receptor 2	endothelin b receptor
endothelin-1 receptor	ephrin type-a receptor 2	estrogen receptor alpha	fibroblast growth factor 1
fibroblast growth factor receptor 1	fibroblast growth factor receptor 2	fibroblast growth factor receptor 3	fibroblast growth factor receptor 4
gamma-aminobutyric acid type b receptor subunit 1	glucagon-like peptide 1 receptor	Glucocerebroside	glutamate receptor 1
guanylate cyclase soluble subunit alpha-2	hepatocyte growth factor receptor	high affinity nerve growth factor receptor	histamine h1 receptor
integrase	interleukin-1 beta	kappa-type opioid receptor	lim domain kinase 1
mast/stem cell growth factor receptor kit	mitogen-activated protein kinase 11	muscarinic acetylcholine receptor m1	muscarinic acetylcholine receptor m2
muscarinic acetylcholine receptor m3	muscarinic acetylcholine receptor m4	muscarinic acetylcholine receptor m5	mu-type opioid receptor
nmda receptor	non-receptor tyrosine-protein kinase tyk2	nuclear receptor subfamily 1 group i member 2	p2y purinoceptor 12
platelet-derived growth factor receptor alpha	platelet-derived growth factor receptor beta	potassium channel subfamily k member 2	potassium voltage-gated channel subfamily h member 2
prostaglandin g/h synthase 2	proteasome subunit beta type-1	proteasome subunit beta type-10	proteasome subunit beta type-2
proteasome subunit beta type-5	proteasome subunit beta type-8	proteasome subunit beta type-9	protein cereblon
prothrombin	proto-oncogene tyrosine-protein kinase receptor ret	proto-oncogene tyrosine-protein kinase src	raf proto-oncogene serine/threonine-protein kinase
receptor tyrosine-protein kinase erbb-2	receptor-type tyrosine-protein kinase flt3	reverse transcriptase/rnaseh	serine/threonine-protein kinase b-raf
serine/threonine-protein kinase mtor	serine/threonine-protein kinase nek11	serine/threonine-protein kinase sik1	sh2b adapter protein 3
sodium channel protein type 1 subunit alpha	sodium-dependent noradrenaline transporter	sodium-dependent serotonin transporter	solute carrier family 6 member 2
somatostatin receptor type 1	somatostatin receptor type 2	somatostatin receptor type 3	somatostatin receptor type 5
synaptic vesicular amine transporter	thrombopoietin receptor	thymidylate synthase	t-lymphocyte activation antigen cd80
t-lymphocyte activation antigen cd86	tubulin alpha-4a chain	tubulin beta-1 chain	tumor necrosis factor
type-1 angiotensin ii receptor	tyrosine-protein kinase abl1	tyrosine-protein kinase frk	tyrosine-protein kinase itk/tsk
tyrosine-protein kinase jak1	tyrosine-protein kinase jak2	tyrosine-protein kinase jak3	tyrosine-protein kinase lck
tyrosine-protein kinase lyn	uric acid	vascular endothelial growth factor receptor 1	vascular endothelial growth factor receptor 2
vascular endothelial growth factor receptor 3	voltage-dependent l-type calcium channel subunit alpha-1c	voltage-dependent l-type calcium channel subunit alpha-1d	voltage-dependent l-type calcium channel subunit alpha-1f
voltage-dependent l-type calcium channel subunit alpha-1s	voltage-dependent l-type calcium channel subunit beta-1	voltage-dependent l-type calcium channel subunit beta-2	voltage-dependent l-type calcium channel subunit beta-3
voltage-dependent l-type calcium channel subunit beta-4		xanthine dehydrogenase/oxidase	
